# *Orthosiphon stamineus* Proteins Alleviate Hydrogen Peroxide Stress in SH-SY5Y Cells

**DOI:** 10.3390/life11060585

**Published:** 2021-06-20

**Authors:** Yin-Sir Chung, Pervaiz Khalid Ahmed, Iekhsan Othman, Mohd. Farooq Shaikh

**Affiliations:** 1 Neuropharmacology Research Laboratory, Jeffrey Cheah School of Medicine and Health Sciences, Monash University Malaysia, Bandar Sunway 47500, Malaysia; mymycys@gmail.com (Y.-S.C.); Iekhsan.Othman@monash.edu (I.O.); 2 School of Business, Monash University Malaysia, Bandar Sunway 47500, Malaysia; pervaiz.ahmed@monash.edu; 3 Global Asia in the 21st Century (GA21), Monash University Malaysia, Bandar Sunway 47500, Malaysia; 4 Liquid Chromatography-Mass Spectrometry (LCMS) Platform, Jeffrey Cheah School of Medicine and Health Sciences, Monash University Malaysia, Bandar Sunway 47500, Malaysia

**Keywords:** *Orthosiphon stamineus*, plant-derived proteins, neuroprotective, SH-SY5Y cell model, hydrogen peroxide

## Abstract

The neuroprotective potential of *Orthosiphon stamineus* leaf proteins (OSLPs) has never been evaluated in SH-SY5Y cells challenged by hydrogen peroxide (H_2_O_2_). This work thus aims to elucidate OSLP neuroprotective potential in alleviating H_2_O_2_ stress. OSLPs at varying concentrations were evaluated for cytotoxicity (24 and 48 h) and neuroprotective potential in H_2_O_2_-induced SH-SY5Y cells (24 h). The protective mechanism of H_2_O_2_-induced SH-SY5Y cells was also explored via mass-spectrometry-based label-free quantitative proteomics (LFQ) and bioinformatics. OSLPs (25, 50, 125, 250, 500, and 1000 µg/mL; 24 and 48 h) were found to be safe. Pre-treatments with OSLP doses (250, 500, and 1000 µg/mL, 24 h) significantly increased the survival of SH-SY5Y cells in a concentration-dependent manner and improved cell architecture—pyramidal-shaped cells, reduced clumping and shrinkage, with apparent neurite formations. OSLP pre-treatment (1000 µg/mL, 24 h) lowered the expressions of two major heat shock proteins, HSPA8 (heat shock protein family A (Hsp70) member 8) and HSP90AA1 (heat shock protein 90), which promote cellular stress signaling under stress conditions. OSLP is, therefore, suggested to be anti-inflammatory by modulating the “signaling of interleukin-4 and interleukin-13” pathway as the predominant mechanism in addition to regulating the “attenuation phase” and “HSP90 chaperone cycle for steroid hormone receptors” pathways to counteract heat shock protein (HSP)-induced damage under stress conditions.

## 1. Introduction

Worldwide, central nervous system (CNS) disorders remain one of the greatest threats in public health, and they account for a significant proportion of the global disease burden [1,2]. These disorders may involve a wide variety of mechanisms but share some common themes, including abnormal protein behavior, oxidative stress, mitochondrial dysfunction, excitotoxicity, ion imbalance, cellular inflammation, cytotoxicity, necrosis, apoptosis, and others [3,4,5,6,7].

Neuroprotection has been explored as a possible treatment strategy [6,8] that aims to prevent neuronal injury and loss of various brain functions with the ultimate goal of better preserving brain function [9].

*Orthosiphon stamineus* (OS) or *Orthosiphon aristatus var. aristatus* (OAA) is a medicinal plant belonging to the Lamiaceae family. Often, it is referred to as “cat’s whiskers” or “misai kucing”. A plethora of studies on the crude extracts or secondary metabolites of OS has shown protective effects, including antioxidative, anti-inflammatory, antiproliferative, cytotoxic, and antiangiogenic effects [10,11]. Added to that, OS has recently been reported for its neuroprotective effects [12]. In another recent study, OS leaf proteins (OSLPs) alleviated pentylenetetrazol-induced seizures in adult zebrafish [13]. The protein compositions identified with important neuroprotective potential include rosmarinate synthase (transferase family), beta-myrcene synthase and R-linalool synthase (terpene synthase family), baicalein 7-O-glucuronosyltransferase (cytochrome P450 family), and baicalin-beta-D-glucuronidase (glycosyl hydrolase 79 family) [13].

Many biological processes are simultaneously active and coordinated in every living cell. Each of them contains synthesis, catalysis, and regulation functions, which are almost always performed by proteins organized in higher-order structures and networks. For decades, people have been using biochemical and biophysical methods to study the structure and function of selected proteins. However, the properties and behavior of the proteome as an integrated system remain largely elusive. Powerful technology based on mass spectrometry now allows the identification, quantification, and characterization of proteins in terms of the composition, structure, function, and control of the proteome, revealing complex biological processes and phenotypes. Proteomics has been described as an important method for obtaining biological information because most biological activities are attributed to proteins, thus improving our concept of biological systems. [14,15]. Proteomics allows us to visualize the highly dynamic cascades of events with peptide-level information, not limited to a static point, as we can see in the Reactome Database, wherein each reaction, interaction, and pathway that happens throughout a whole biological event is depicted with its proteomics details [16,17,18].

Human neuroblastoma cell line, SH-SY5Y, with a stable karyotype consisting of 47 chromosomes, is an in vitro model ideal for high-throughput studies on neurobiology [19]. The SH-SY5Y model provides an efficient platform that is essential for preliminary drug testing, protein functionality, and molecular mechanisms in neurological conditions [20]. Hydrogen peroxide (H_2_O_2_) insults have been prevalently reported in different neurological disorders, including neuroexcitation, neuroinflammation, and neurotoxicity, just to name a few [21,22,23]. This study was commenced to evaluate the neuroprotective potential of OSLPs in SH-SY5Y cells induced by H_2_O_2_.

## 2. Materials and Methods

### 2.1. Materials, Chemicals, and Apparatuses

Human SH-SY5Y neuroblastoma cells (ATCC^®^CRL-2266TM) were purchased from the American Type Culture Collection (ATCC, Manassas, VA, USA). Fetal bovine serum (FBS) and penicillin–streptomycin mixture (Pen/Strep) were purchased from PAA Laboratories (Austria). Hemocytometer BLAUBRAND^®^ Neubauer, Dulbecco′s modified Eagle′s medium (DMEM), 3-(4,5-methylthiazol-2-yl)-2,5-diphenyl-tetrazolium bromide (MTT), complete EDTA-free protease inhibitors, phosphatase inhibitors cocktail 2, hydrogen peroxide (H_2_O_2_), TRIS hydrochloride (TRIS-HCl), dithiothreitol (DTT), iodoacetamide (IAA), HPLC-grade methanol (MeOH), ammonium bicarbonate (ABC), trifluoroethanol (TFE), formic acid (FA), and 2,3,5-triphenyltetrazolium chloride (TTC) were purchased from Sigma-Aldrich (St. Louis, MO, USA). Trypsin/Lys-C Mix (Promega, USA), T-25 flasks (Corning Inc., Tewksbury, MA, USA), 15 mL Falcon tubes (BD Biosciences, Billerica, MA, USA), TrypLE^™^ Express (Life Technologies, Nærum, Denmark), and phosphate-buffered saline solution (10XPBS) (Abcam, Hangzhou, China) were also purchased. Pierce^®^ trypsin protease, mass spec grade Pierce^®^ radioimmunoprecipitation assay (RIPA) buffer as well as Pierce^®^C18 mini spin columns were purchased from Thermo Scientific Pierce (Waltham, MA, USA). Protein LoBind microcentrifuge tubes were purchased from Eppendorf (Framingham, MA, USA), a Quick Start^™^ Bradford Protein Assay Kit from Bio-Rad (Irvine, CA, USA), trifluoroacetic acid (TFA), acetonitrile (ACN), and mass-spec grade CHAPS (Nacailai Tesque, Kyoto, Japan) were all purchased from Sigma-Aldrich (St. Louis, MO, USA). Milli-Q ultrapure water (MQUP) was from Millipore GmbH (Germany); dimethylsulfoxide (DMSO) and 37% formaldehyde solution were purchased from Friendemann Schmidt Chemical (Parkwood, WA, Australia). Refrigerated centrifuge 5415R from Eppendorf AG (Hamburg, Germany), hydrochloric acid (36%) from Ajax Chemical (Australia), and acetic acid (glacial, 100%) from Merck (Darmstadt, Germany) were also purchased. Purified nitrogen gas (99.999%) was supplied by Merck (Darmstadt, Germany) Iwatani Malaysia S/B, and liquid nitrogen (LN_2_) was purchased from Linde Malaysia. An ultrasonic cell crusher (JY88-II N, Shanghai, China), an Eyela SpeedVac Vacuum Concentrator (Thermo Scientific Pierce, Waltham, MA, USA), a precision incubator (Memmert INB200, Schwabach, Germany), and a Cole-Parmer^™^ Stuart^™^ Orbital Shaker (Thermo Scientific Pierce, Waltham, MA, USA) were also purchased. All the other chemicals used were of analytical grade.

### 2.2. Software and Equipment

An Olympus CKX41 inverted trinocular microscope (Manila, Philippines) connected to an Olympus UIS2 optical system camera and AnalySIS 1.5 software were used for the microscopic examination of SH-SY5Y cells.

In the protein expression study, an Agilent 1200 series HPLC paired with an Agilent 6550 iFunnel quadrupole time of flight (Q-TOF) LC/MS, a C-18 300Ǻ large capacity chip, and Agilent MassHunter data acquisition software (all from Agilent Technologies, USA) were used to determine the differentially expressed proteins. Additionally, version 8.0 of PEAKS^®^Studio software (Bioinformatics Solution, Waterloo, ON, Canada) and the UniProtKB database (organism: *Homo sapiens*) were used to analyze the results of the mass-spectrometry-based label-free quantitative proteomics (LFQ). Cytoscape software, with version 3.7.2 of the BiNGO plugin, was used for Gene Ontology (GO)-annotated information (Cytoscape Consortium, California, USA). Reactome Pathway Browser version 3.7 and Reactome Database Release 72 (organism: *Homo sapiens*) were utilized for the investigation into protein–protein interactions, functional annotations, and systemic pathway enrichment analysis.

### 2.3. Experimental Design

#### 2.3.1. Extraction and Identification of Proteins by Nanoflow Liquid Chromatography Electrospray Ionization Coupled with Tandem Mass Spectrometry/Mass Spectrometry (Nanoflow-ESI-LCMS/MS)

The OS plants, aged about 12 months old (voucher specimen 11,009), were collected from Kampung Repuh, Batu Kurau (GPS coordinates: 4.52° N, 100.48° E; Perak, Malaysia). The fresh leaves were collected, cleaned, flash-frozen using liquid nitrogen, and ground into a fine powder using a pre-chilled grinder and ultrasonic cell crusher. The leaf powder was then weighed (50 mg) and kept in sterile 2.0 mL Eppendorf Protein LoBind^®^ microtubes. The one-tube method was modified from previous studies [24,25,26]. The supernatants produced were then harvested and subjected to vacuum concentration (300 rpm; 24 h; 40 °C). Next, in-solution protein digestion was carried out based on the manufacturer’s instructions (Mass Spec Grade Promega, USA). The digested peptides were loaded onto a C-18 300Ǻ large capacity chip (Agilent, USA) and separated using a binary buffer system. The column was equilibrated by Buffer 1 (0.1% FA in MQUP) and Buffer 2 (60% ACN containing 0.1% FA). The digested peptides were eluted with a linear gradient: 50 min in 0–40% Buffer 2 followed by 40–80% Buffer 2 for an additional 30 min. Quadrupole time of flight (Q-TOF) was set at positive polarity, capillary voltage at 2050 V, fragmentor voltage at 300 V, drying gas flow 5 L/min, and a gas temperature of 300 °C. The peptide spectrum was analyzed in auto MS mode, ranging from 110–3000 m/z for the MS scan and 50–3000 m/z for the MS/MS scan, followed by up to 15 data-dependent MS/MS scans (top 15 approaches), with higher-energy collisional dissociation (HCD) at a resolution of 17,500 at 200 m/z. Dynamic exclusion was set to 30 s. Agilent MassHunter data acquisition software (version B.07.00, Agilent Technologies, Santa Clara, CA, USA) and PEAKS^®^ Studio software (version 7.5, Bioinformatics Solutions Inc., Waterloo, ON, Canada) were used for the spectrum analysis. Next, the Lamiaceae protein databases of UniProtKB (http://www.uniprot.org/uniprot/ accessed on 10 January 2020) and NCBInr (https://www.ncbi.nlm.nih.gov/ accessed on 10 January 2020) were downloaded. Protein identification and homology search by comparing the de novo sequence tags were assisted by PEAKS^®^ Studio (version B.07.00). The settings applied were as follows: both parent mass and precursor mass tolerance were set at 0.1 Da with monoisotopic as the precursor mass search type; carbamidomethylation was set as a fixed modification, with maximum missed cleavage set at 3; maximum variable post-translational modification was set at 3, and trypsin/Lys-C was selected as the digestion enzyme. The other parameters were set as default by Agilent. The filtration parameters were set at a significant score (−10logP) of protein ≥20 and the number of peptides ≥20 to exclude inaccurate proteins. PEAKS^®^ indicated that a −10logP score of greater than 20 is relatively high in confidence as it targets very few decoy matches above the threshold [27] (see Supplementary Table S1).

#### 2.3.2. SH-SY5Y Cells—Initial Culture, Sub-Culture, and Seeding Conditions

The SH-SY5Y cells obtained were maintained in an initial culture medium (pre-warmed to 37 °C) consisting of DMEM supplemented with 10% FBS and 1% Pen/Strep and kept in an incubator at 37 °C with 5% CO_2_ and 95% air. The initial culture medium was refreshed every 4–7 days to remove non-adherent cells and to replenish nutrients and was monitored for cell confluence. When the cells reached ≥80% confluence, the sub-culture was performed. The old initial culture medium was aspirated, and the T-25 flask was rinsed with 1 mL of warm 1X PBS (5 s, twice). To lift the cells, 1 mL of TrypLE^™^ Express was added, and the flask was incubated (5–10 min, 37 °C, 5% CO_2_, and 95% air). The flask was removed and observed under a microscope to confirm the detachment of cells (SH-SY5Y cells were seen as “floating”). The cell suspension produced was very gently transferred to a sterile 15 mL Falcon tube containing 1 mL of 1X PBS (37 °C). The tube was centrifuged (1000 rpm, 3 min, r.t.). The supernatant produced was gently discarded without disturbing the soft, transparent cell pellet formed at the bottom. The cell pellet was re-suspended in 1 mL fresh growth medium consisting of DMEM supplemented with 1% FBS and 1% Pen/Strep (pre-warmed to 37 °C) and was ready for seeding into the plates. In this study, the cells used for each experiment were of less than 20 passages.

#### 2.3.3. Evaluation of Cytotoxic Effects of OSLPs on SH-SY5Y Cells (24 and 48 h)

SH-SY5Y cells (5 × 10^4^) were seeded in 96-well plates (*n* = 3). Vacuum-concentrated OSLP was diluted in the growth medium at a concentration range of 25, 50, 125, 250, 500, 1000, 2000, 4000, and 10,000 µg/mL. The cells were then treated with OSLP at varying concentrations and incubated for 24 and 48 h (37 °C, 5% CO_2_, 95% air). Upon complete incubation, both treatment groups were evaluated for cytotoxic effects using MTT assays. Absorbance was read at wavelength 570 nm with the reference filter set at 690 nm. All experiments were 3 independent biological replicates performed in triplicate, and the relative cell viability is expressed as a percentage (%) relative to the untreated control cells (normal control). Additionally, the maximal non-toxic dose (MNTD) and minimal toxic dose (MTD) of OSLP at 24 and 48 h were also determined [28].

(1) $$Cell\;viability\;\left(\%\right)=\frac{Absorbance\;of\;sample-Absorbance\;of\;blank}{Absorbance\;of\;control-Absorbance\;of\;blank}\times 100$$

#### 2.3.4. Hydrogen Peroxide (H_2_O_2_) Induction and Determination of Half-Maximal Inhibitory Concentration (IC_50_)

SH-SY5Y cells (5 × 10^4^) were seeded in 96-well plates (*n* = 3). SH-SY5Y cells were induced by H_2_O_2_ at concentrations of 0, 50, 100, 150, 200, 250, 300, and 350 µM. All concentrations of H_2_O_2_ were freshly prepared by diluting a 30.2% (*v*/*v*) stock solution with DMEM. Following that, the H_2_O_2_-induced cells were incubated at 37 °C with 5% CO_2_ and 95% air for 24 h. Upon completion of incubation, cell viability (%) of the SH-SY5Y cells, the half-maximal inhibitory concentration (IC_50_), and the maximal inhibitory concentration (IC_90_) were determined using an MTT assay. All experiments were 3 independent biological replicates performed in triplicate.

#### 2.3.5. Evaluation of OSLP Protective Effects on SH-SY5Y Cells

SH-SY5Y cells (5 × 10^4^) were seeded in 96-well plates (*n* = 6). Vacuum-concentrated OSLP was diluted in the growth medium at a concentration range of 25, 50, 125, 250, 500, and 1000 µg/mL. The cells were assigned to a total of 8 groups, namely, normal control (NC) without H_2_O_2_ induction and OSLP treatments; negative control (Neg C, H_2_O_2_), which was induced by 150 µM of H_2_O_2_; and six OSLP treatment groups that received six different concentrations (25–1000 μg/mL) (Table 1). All six treatment groups were pre-treated with OSLP and incubated for 24 h at 37 °C, with 5% CO_2_ and 95% air. Following that, all six groups were treated with Eppendorf Protein LoBind^®^. Upon completion of incubation, all 8 experiment groups were evaluated using MTT assays. All experiments were 6 independent biological replicates performed in triplicate.

#### 2.3.6. Microscopic Examination Using Bright-Field Imaging

Microscopic changes (10×) of the SH-SY5Y cells were studied using bright-field microscopy. The bright-field microscopic images of the normal control (NC), the negative control (H_2_O_2_ induced by 150 µM H_2_O_2_), and three OSLP treatment groups (250, 500, and 1000 µg/mL) were captured with an Olympus CKX41 inverted trinocular microscope connected to an Olympus UIS2 optical system camera and AnalySIS 1.5 software.

### 2.4. Protein Expression Study

#### 2.4.1. Protein Expression Profiling with Mass Spectrometry-Based Label-Free Quantitative Proteomics (LFQ)

OSLP was prepared in a concentration of 10 mg/mL (as mother stock) and was then twofold diluted to 250, 500, and 1000 µg/mL in fresh growth medium (DMEM with 1% FBS and 1% Pen/Strep). SH-SY5Y cells (1 × 10^6^) were seeded in 6-well plates. The cells were assigned to 5 groups (Table 2). Three treatment groups were pre-treated with freshly prepared OSLP and incubated for 24 h (37 °C, 5% CO_2_, 95% air). Following that, they were induced by 150 µM of H_2_O_2_ for another 24 h and returned to incubation (37 °C, 5% CO_2_, 95% air). Upon complete incubation, all five experiment groups were subject to cell lysis for protein extraction in order to conduct mass-spectrometry-based label-free quantitative proteomics (LFQ). For all experiments, 3 independent biological replicates were performed.

#### 2.4.2. Protein Extraction from SH-SY5Y Cells

After aspirating the media, the cells were treated with TrypLE™ Express, incubated, and rinsed with pre-cooled 1X PBS. The content was collected into individual sterile Eppendorf Protein LoBind^®^ microtubes and centrifuged (500× *g*, 4 °C; 10 min). The produced supernatant was discarded, but the soft, transparent pellet was collected and lysed with ice-cold lysis buffer (200 μL of RIPA, protease inhibitor 20% *v*/*v*, phosphatase inhibitor 1% *v*/*v*) and incubated (4 °C; 20 min). Following that, the cell suspension was homogenized using an ultrasonic cell crusher and then briefly centrifuged (2000× *g*, 4 °C; 10 min). The proteins extracted were collected into new, individual, sterile Eppendorf Protein LoBind^®^ microtubes and were concentrated using a speed-vacuum concentrator (300 rpm; 24 h; 60 °C) before storage at −152 °C for subsequent analysis.

#### 2.4.3. Protein Estimation by Bradford Protein Assay

Protein concentration was estimated using a Quick Start™ Bradford protein assay, following the instructions of the manufacturer. Briefly, 5μL of the sample or standard was loaded onto a 96-well plate in triplicate. This was followed by adding 250 μL of dye reagent into each well. The plate was incubated at room temperature (25–27 °C; 5 min). Absorbance was read at 595 nm with a Bio-Rad Benchmark Plus microplate reader with Microplate Manager 5.2.1 software. Protein concentrations were determined from the standard curve.

#### 2.4.4. In-Solution Digestion of Proteins

In-solution protein digestion was performed as instructed (Mass Spec Grade Promega, Madison, WI, USA). Protein samples were solubilized in 6 M urea/50 mM TRIS-HCl (pH 8.02), followed by the addition of 5 mM DTT (freshly prepared) and incubated in the dark (30 min; 37 °C). Next, 15 mM IAA (freshly prepared) was added and incubated in the dark (30 min; r.t.). The reduced and alkylated protein solutions were diluted sixfold with 50 mM TRIS-HCl (pH 8.02). Following that, 20 μg of crude protein was digested by trypsin/Lys-C mix (ratio 25 protein:1 protease; *w*/*w*) buffered in 50 mM TRIS-HCl (pH 8.02) and then incubated in the dark (overnight; 37 °C). Formic acid (1%) was added to halt the enzymatic reaction. Following that, all the samples were subjected to centrifugation (16,000× *g*; 4 °C; 10 min). The supernatant produced was collected and concentrated using a speed-vacuum concentrator (300 rpm; 24 h; 60 °C). Formic acid (10 μL of 0.1%) was added into all the sample tubes, followed by brief vortexing and centrifugation.

#### 2.4.5. De-Salting of Proteins

Each protein biological replicate was independently de-salted using modified instructions for the Pierce^®^C18 mini spin column. Every mini spin column was firstly activated using a 50% ACN solution (repeated thrice, r.t.) and equilibrated using a 0.5% solution of TFA in 5% ACN (repeated thrice, r.t.). A 90 μL volume of protein was individually added into a 30 μL solution of sample buffer (2% of TFA in 20% of ACN) and momentarily vortexed at a speed of 2200 rpm to ensure proper mixing. This step was repeated individually for each protein biological replicate. Next, each of them was loaded onto individual sterile mini spin columns for de-salting (repeated thrice, r.t.). Subsequently, each protein biological replicate was washed using a 0.5% solution of TFA in 5% ACN (repeated thrice, r.t.). Finally, each protein biological replicate was eluted using a 70% solution of ACN (repeated thrice, r.t.), and all the produced flow-through was collected, vacuum-concentrated (300 rpm; 24 h; 60 °C), and then stored at −20 °C for mass-spectrometry-based LFQ at a later date.

#### 2.4.6. Mass-Spectrometry-Based Label-Free Quantitative Proteomics (LFQ) Using Nanoflow-ESI-LCMS/MS

An Agilent C-18 300Ǻ large capacity chip was used to load the previously de-salted peptides. The column was equilibrated using 0.1% FA in water (Buffer 1), and the peptides were eluted using an increasing gradient of 90% ACN in 0.1% FA (Buffer 2) using the following gradient: 3–50% Buffer 2 from 0–30 min, 50–95% Buffer 2 from 30–32 min, 95% Buffer 2 from 32–39 min, and 95–3% Buffer 2 from 39–47 min. The Q-TOF settings were as follows: positive polarity, fragmentor voltage at 300 V, capillary voltage at 2050 V, drying gas at a flow rate of 5 L/min, and a 300 °C gas temperature. Auto MS/MS mode was used to analyze the intact protein, with a range of 110–3000 m/z for the MS scan and a 50–3000 m/z range for the MS/MS scan. Agilent MassHunter data acquisition software was used to perform the spectrum analysis.

#### 2.4.7. Peptide and Protein Identification by Automated De Novo Sequencing and LFQ Analysis

The UniProtKB database (Organism: *Homo sapiens*) (https://www.uniprot.org/proteomes/UP000005640, 163,191 proteins; accessed on 13 March 2020) was used to identify the peptides and proteins, as well as conduct homology searching via comparison of the de novo sequence tag, using the following settings: trypsin cleavage, a parent mass and a precursor mass tolerance of 0.1 Da, minimum ratio count of 2, maximum variable post-translational modification of 3, carbamidomethylation as a fixed modification with maximum missed cleavage of 3, mass error tolerance of 20.0 ppm, and other parameters as default settings of Agilent. The false discovery rate (FDR) threshold was set at 1%, and a protein score of −10lgP > 20 was used to filter out proteins that were inaccurate. PEAKS^®^ software indicated that a protein score of −10lgP >20 has relatively high confidence as it targets very few decoy matches above the threshold.

The differentially expressed proteins were identified using LFQ analysis using the following settings: significance score ≥13, protein fold change ≥1, number of unique peptides ≥1, and an FDR threshold of ≤1%. PEAKSQ indicated that a significance score of ≥13 is equal to a significance value of *p* < 0.05. All other parameters were kept at the default settings set by Agilent.

### 2.5. Bioinformatics Analysis

Using bioinformatics analysis (functional annotations, protein–protein interactions, and systemic pathway enrichment analysis) of the identified differentially expressed proteins, the proteins were analyzed and matched using the GO Consortium, Ensemble (http://www.ensembl.org/Homo_sapiens accessed on 13 December 2019), and Reactome Database (Release 72; organism: *Homo sapiens*) online databases.

### 2.6. Statistical Analysis

Statistical analysis was carried out using version 5.0 of GraphPad Prism. The data obtained from the in vitro assays were expressed using the notation of mean ± standard error of the mean (SEM). One-way ANOVA followed by Dunnett’s post hoc test was used to compare data between the control and treated groups using the significance levels of * *p* < 0.5, ** *p* < 0.01, and *** *p* < 0.001. The built-in statistical tool of PEAKS^®^ software (PEAKSQ statistical analysis) was used to analyze the identified differentially expressed proteins. A 13% significance score (which is equal to a significance level of 0.05) and an FDR of ≤1% are considered to be statistically significant. In the bioinformatics analysis, the hypergeometric test followed by Benjamini and Hochberg’s FDR correction at *p*-value <0.05 (built-in BiNGO statistical tool) was used to correlate the functional annotation of genes with their interacting proteins; overrepresentation analysis of pathways was tested with hypergeometric distribution, following the Benjamani-Hochberg method, corrected at *p*-value <0.05 (Reactome Pathway Browser version 3.7 built-in statistical tool). The overrepresentation analysis of Reactome Pathways was used to predict the possible associations of systemic pathways with their interacting proteins and genes.

## 3. Results

### 3.1. Evaluation of Cytotoxic Effects of OSLP on SH-SY5Y Cells (24 and 48 h)

**Figure 1:**
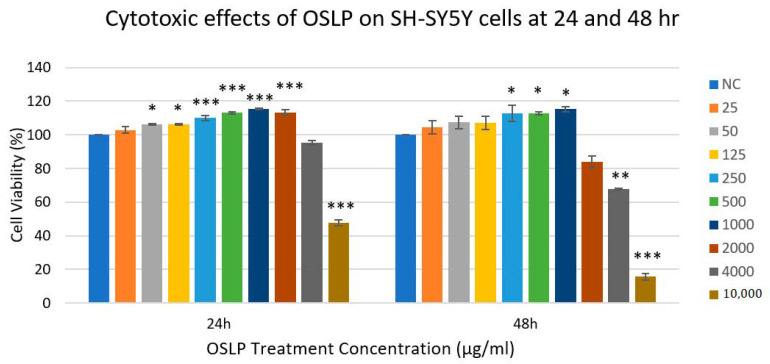
Cytotoxic effects of OSLP on SH-SY5Y cells at 24 and 48 h. Data shown are presented as mean ± SEM of 3 independent experiments performed in triplicate. * *p* < 0.05, ** *p* < 0.01 and *** *p* < 0.001 against the normal control group (NC). One-way ANOVA with Dunnett’s post hoc test.

After 24 h incubation, no significant cytotoxic effects of OSLP were observed at concentrations below 4000 µg/mL compared to the NC (F = 251.7; *p* > 0.05; Figure 1). Cytotoxic effects were apparent when the SH-SY5Y cells were treated with 4000 µg/mL of OSLP (95 ± 1%). This slight reduction, however, did not attain any statistical significance when compared to the NC (F = 251.7; *p* > 0.05; Figure 1). In contrast, treatment with 10 mg/mL of OSLP was found to result in a significant decrease, about 52 ± 2%, compared to the NC (F = 251.7; ^^^ *p* < 0.001; Figure 1).

After 48 h incubation, no significant cytotoxic effects of OSLP were observed at concentrations below 2000 µg/mL compared to the NC (F = 106.6; *p* > 0.05; Figure 1). Significant cytotoxic effects of OSLP were apparent at concentrations above 2000 µg/mL compared to the NC (F = 106.6; ** *p* < 0.01; Figure 1). At 2000 µg/mL of OSLP, cell viability significantly decreased to 84 ± 4% (F = 106.6; ** *p* < 0.01; Figure 1) and declined further to 68 ± 0.5% at 4000 µg/mL of OSLP (F = 106.6; *** *p* < 0.001; Figure 1). A significant plunge, about 84 ± 2%, in the SH-SY5Y cell population was observed at 10 mg/mL of OSLP treatment (F = 106.6; *** *p* < 0.001; Figure 1). This indicates that 10 mg/mL of OSLP exerted significant cytotoxic effects on the survival of SH-SY5Y cells.

From the graph plotted (Figure 1), the MNTD of OSLP at 24 h treatment was determined as approximately 2000 µg/mL, whilst the MTD of OSLP at 24 h treatment was approximately 4000 µg/mL. In contrast, the MNTD of OSLP at 48 h treatment was determined as approximately 1000 µg/mL, whereas the MTD of OSLP at 48 h treatment was approximately 2000 µg/mL.

### 3.2. Hydrogen Peroxide (H_2_O_2_) Induction and Determination of Half-Maximal Inhibitory Concentration (IC_50_)

**Figure 2:**
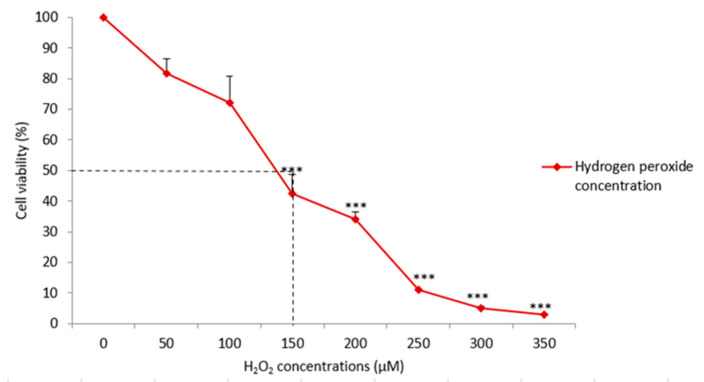
Cell viability of SH-SY5Y cells induced by H_2_O_2_. SH-SY5Y cells were treated with 0–350 µM H_2_O_2_. Data shown are presented as mean ± SEM of 3 independent experiments performed in triplicate. *** shows *p* < 0.001 against the untreated group (NC, 24 h). One-way ANOVA with Dunnett’s post hoc test.

As depicted in Figure 2, exposure from 50 to 350 µM of H_2_O_2_ decreased the cell population in a concentration-dependent manner. Cell viability (%) decreased when H_2_O_2_ concentrations increased. When compared to the NC, 50–100 µM of H_2_O_2_ did not significantly inhibit SH-SY5Y cell growth (F = 105.6; *p* > 0.5; Figure 2) but 150–350 µM of H_2_O_2_ significantly inhibited SH-SY5Y cell growth (F = 105.6; *** *p* < 0.001; Figure 2). At about 150 µM of H_2_O_2_, cell viability was reduced significantly to 42 ± 6% (F = 105.6; *** *p* < 0.001) and further declined significantly to 34 ± 3% (F = 105.6; *** *p* < 0.001) when the concentration increased to 200 µM growth. Following that, cell viability tumbled steeply to 11 ± 0.4%, 3 ± 0.5%, and 5 ± 0.2% when H_2_O_2_ induction increased to 250, 300, and 350 µM, respectively (F = 105.6; *** *p* < 0.001). From the graph plotted (Figure 2), the IC_50_ of H_2_O_2_ was determined as approximately 150 μM whilst the IC_90_ of H_2_O_2_ was determined as 250 μM and above.

### 3.3. Evaluation of OSLP Protective Effects on SH-SY5Y Cells

**Figure 3:**
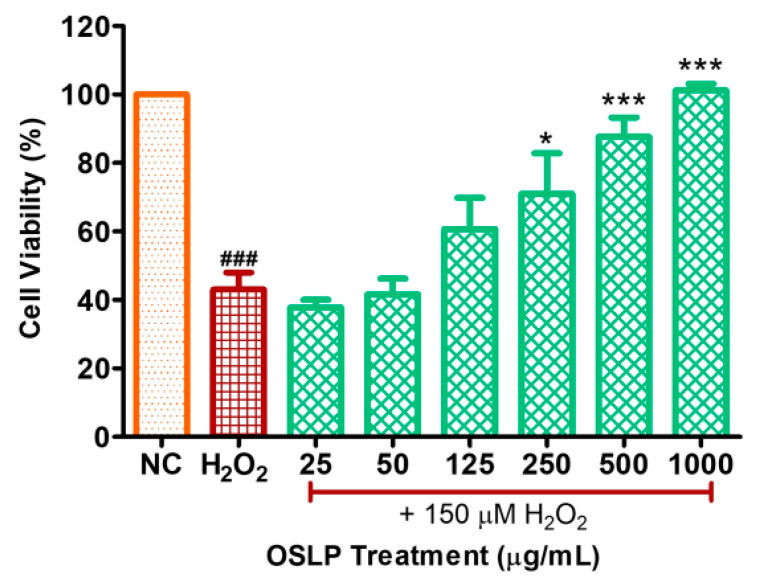
OSLP protective effects on H_2_O_2_-induced SH-SY5Y cells. Data shown are presented as mean ± SEM of 3 independent experiments performed in triplicate. * *p* < 0.05 and *** *p* < 0.001 against against the negative control group (H_2_O_2_, 150 µM), whereas ^###^ shows *p* < 0.001 against the normal control group (NC, no OSLP treatment, and H_2_O_2_ induction). One-way ANOVA with Dunnett’s post hoc test.

From the graph plotted (Figure 3), H_2_O_2_ induction (negative control, 150 μM) significantly decreased SH-SY5Y cell viability (43 ± 5%; F = 17.9; *** *p* < 0.001) compared to the NC. OSLP at these two concentrations, 25 µg/mL (38 ± 2%; F = 17.9; *p* > 0.5) and 50 µg/mL (42 ± 5%; F = 17.9; *p* > 0.5), did not show significant protection against H_2_O_2_ induction. At 125 µg/mL, OSLP increased cell viability by about 30% compared to the H_2_O_2_ group (61 ± 9%; F = 17.9; *p* > 0.5). OSLP at 250 µg/mL significantly increased SH-SY5Y cell viability (71 ± 12%; F = 17.9; * *p* < 0.01) compared to the H_2_O_2_ group. An increase of 39% in cell viability was recorded. OSLP at these two concentrations, 500 µg/mL (88 ± 6%; F = 17.9; *** *p* < 0.001) and 1000 µg/mL (101 ± 2%; F = 17.9; *** *p* < 0.001), significantly increased SH-SY5Y cell viability compared to the H_2_O_2_ group. OSLP at 500 µg/mL increased by about 51% whilst OSLP at 1000 µg/mL increased by about 57% in cell viability.

#### Microscopic Examination Using Bright-Field Imaging

**Figure 4:**
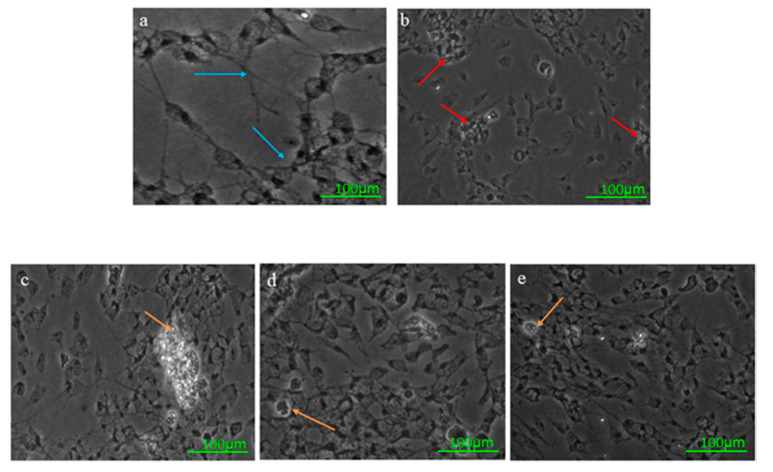
Representative bright-field microscopic images of SH-SY5Y cells. Upper row: (**a**) normal control (NC, without OSLP treatment and H_2_O_2_ induction) displays pyramidal-shaped cells, showing clear neurites (blue arrows), and did not cluster; (**b**) H_2_O_2_ (induced by 150 µM of H_2_O_2_) shows disrupted neuronal cell shapes, with many clumping cells (red arrows) and reduced neurites in addition to a declined population. Lower row: (**c**–**e**) OSLP treatment groups, 250, 500 and 1000 µg/mL, respectively. OSLP treatments reduced clumping cells and restored the neuronal cell shapes, with clear neurites seen (orange arrows). Scale bar = 100 µm.

Figure 4 displays the representative bright-field microscopic images of the SH-SY5Y cells. The NC displayed normal cell architecture, with pyramidal-shaped cells having apparent neurites (panel a, blue arrows). SH-SY5Y cells induced by 150 µM of H_2_O_2_ showed disrupted cell architecture, with clusters of clumping cells and reduced neurites (panel b, red arrows) compared to the normal control (NC), which received no OSLP treatment and no H_2_O_2_ induction (panel a, blue arrows). Pre-treatment with OSLP at 250, 500, and 1000 µg/mL improved the cell architecture, with reduced clumping cells and restored neuronal cell shapes with clear neurites (panels c–e, orange arrows) compared to the negative control (H_2_O_2_,150 µM). The cell population was also markedly declined in the negative control, but pre-treatments with OSLP increased cell growth.

### 3.4. Protein Expression Study

Proteins were extracted from the normal control (NC, SH-SY5Y cells without OSLP treatment and H_2_O_2_ induction), the negative control, (H_2_O_2_, 150 µM H_2_O_2_ only) and three OSLP treatment groups (250 µg/mL + 150 µM H_2_O_2_, 500 µg/mL + 150 µM H_2_O_2_, and 1000 µg/mL + 150 µM H_2_O_2_). The protein samples were subjected to mass-spectrometry-based label-free quantitative proteomics (LFQ) using nanoflow-ESI-LCMS/MS and subsequent bioinformatics analysis. As to the final results and discussion, only these two pairs were used: Pair A, H_2_O_2_ (150 µM H_2_O_2_ only) versus normal control (without OSLP treatment and H_2_O_2_ induction) and Pair B, H_2_O_2_ (150 µM H_2_O_2_ only) versus OSLP treatment (OSLP 1000 µg/mL + 150 µM H_2_O_2_). The highest dose of OSLP was chosen to elucidate its maximal protective effects on SH-SY5Y cells induced by H_2_O_2_.

#### 3.4.1. Protein Expression Analysis with Mass-Spectrometry-Based Quantitative Label-Free Proteomics (LFQ)

**Figure 5:**
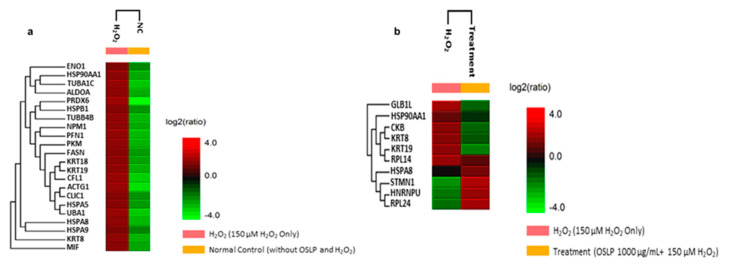
Heat map displays the differentially expressed proteins identified from (**a**) Pair A: H_2_O_2_ (150 µM H_2_O_2_ only) versus NC (normal control without OSLP treatment and H_2_O_2_ induction) and (**b**) Pair B: H_2_O_2_ (150 µM H_2_O_2_ only) versus OSLP treatment (OSLP 1000 µg/mL + 150 µM H_2_O_2_), *n* = 3, significance ≥13, FDR ≤ 1%, fold change ≥1, number of unique peptide ≥1. Protein names are listed on the left, while experimental groups are indicated on top. The color key on the bottom right indicates the log2 (ratio) expression levels (green = low, red = high).

LFQ has profiled 32 differentially expressed proteins, of which 22 were identified in Pair A (H_2_O_2_ vs. NC) and 10 were identified in Pair B (H_2_O_2_ vs. Treatment) (Figure 5, Table 3 and Table 4).

**Figure 6:**
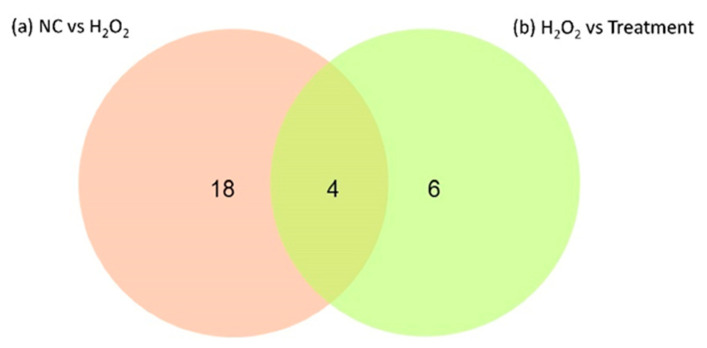
A two-way Venn diagram depicts the differentially expressed proteins identified from (**a**) Pair A: H_2_O_2_ (150 µM H_2_O_2_ only) versus NC (normal control without OSLP treatment and H_2_O_2_ induction) and (**b**) Pair B: H_2_O_2_ (150 µM H_2_O_2_ only) versus OSLP treatment (OSLP 1000 µg/mL + 150 µM H_2_O_2_), *n* = 3. As shown, a total of 32 differentially expressed proteins were identified; 4 are overlaps between the two pairs, 18 are identified in Pair A, and 6 are in Pair B.

In Pair A (H_2_O_2_ vs. NC), all the proteins were found expressed at higher levels in the H_2_O_2_-treated samples than in the NC. In contrast, in Pair B (H_2_O_2_ vs. Treatment), seven proteins were expressed at lower levels in the OSLP-treated group than in the H_2_O_2_-treated group. They were keratin, type II cytoskeletal 8 (KRT8, P05787), heat shock cognate 71 kDa protein (HSPA8, P11142), 60S ribosomal protein L14 (RPL14, P50914), beta-galactosidase-1-like protein (GLB1L, Q6UWU2), keratin, type I cytoskeletal 19 (KRT19, P08727), creatine kinase B-type (CKB, P12277), and heat shock protein HSP 90-alpha (HSP90AA1, P07900). The others, namely, heterogeneous nuclear ribonucleoprotein U (HNRNPU, Q00839), 60S ribosomal protein L24 (RPL24, P83731), and stathmin (STMN1, P16949), were expressed at higher levels in the OSLP-treated group than in the H_2_O_2_-treated group (Figure 5, Table 3 and Table 4). Additionally, four proteins were found expressed in both pairs (Figure 6). They were heat shock cognate 71 kDa protein (HSPA8, P11142), keratin, type II cytoskeletal 8 (KRT8, P05787), keratin, type I cytoskeletal 19 (KRT19, P08727), and heat shock protein HSP 90-alpha (HSP90AA1, P07900). Interestingly, these proteins were found expressed at lower levels in both the NC and the OSLP-treated groups (Figure 5).

#### 3.4.2. Bioinformatics Analysis

**Figure 7:**
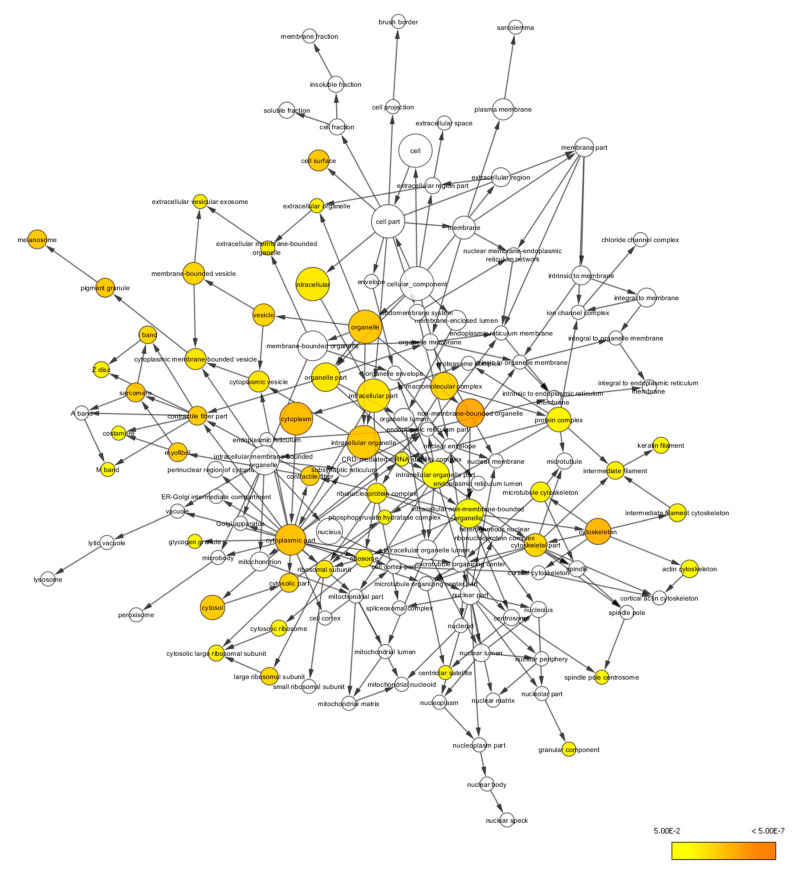
BiNGO result for cellular components, as visualized in Cytoscape (organism: *Homo sapiens*). Colored nodes indicate significant overrepresention. White nodes indicate insignificant overrepresention; they are included to show the colored nodes in the context of the GO hierarchy. The color key on the bottom right indicates the significance level of overrepresentation.

The differentially expressed proteins were also studied using functional annotation analysis to identify and visualize the cellular components, molecular functions, and biological processes of the differentially expressed proteins. The differentially expressed proteins were found to localize at cellular components, including non-membrane-bound organelle (GO:43228), intracellular non-membrane-bound organelle (GO:43232), cytoskeleton (GO:5856), cytoplasm (GO:5737), cytoplasmic part (GO:44444), intracellular organelle (GO:43229), organelle (GO:43226), cell surface (GO:9986), pigment granule (GO:48770), and melanosome (GO:42470) (Figure 7).

**Figure 8:**
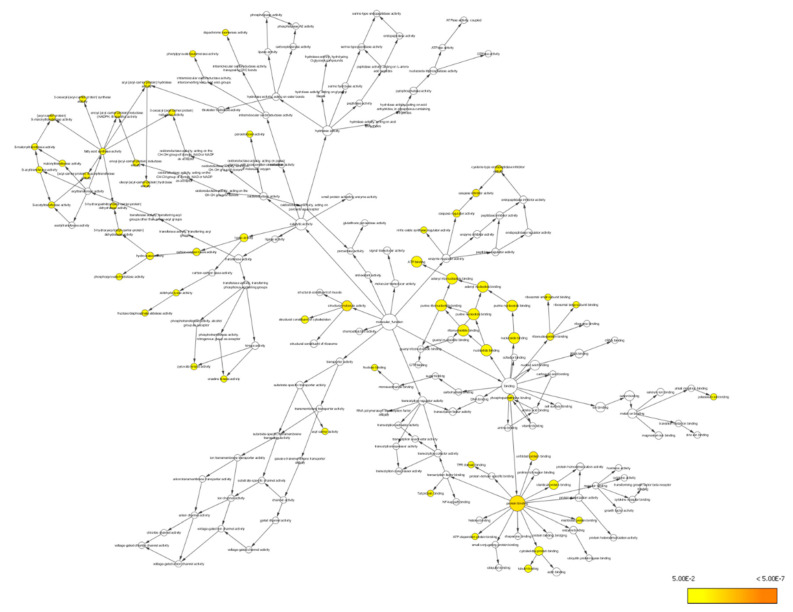
BiNGO results for molecular function, as visualized in Cytoscape (organism: *Homo sapiens*). Colored nodes indicate significant overrepresention. White nodes indicate insignificant overrepresention; they are included to show the colored nodes in the context of the GO hierarchy. The color key on the bottom right indicates the significance level of overrepresentation.

At these cellular localizations, the interactions of the differentially expressed proteins have been networked to an array of molecular functions involved in protein binding (GO:5515), unfolded protein binding (GO:51082), structural molecule activity (GO:5198), caspase inhibitor activity (GO:43027), ATP binding (GO:5524), adenyl ribonucleotide binding (GO:32559), ribonucleotide binding (GO:32553), purine ribonucleotide binding (GO:32555), adenyl nucleotide binding (GO:30554), and purine nucleotide binding (GO:17076) (Figure 8).

**Figure 9:**
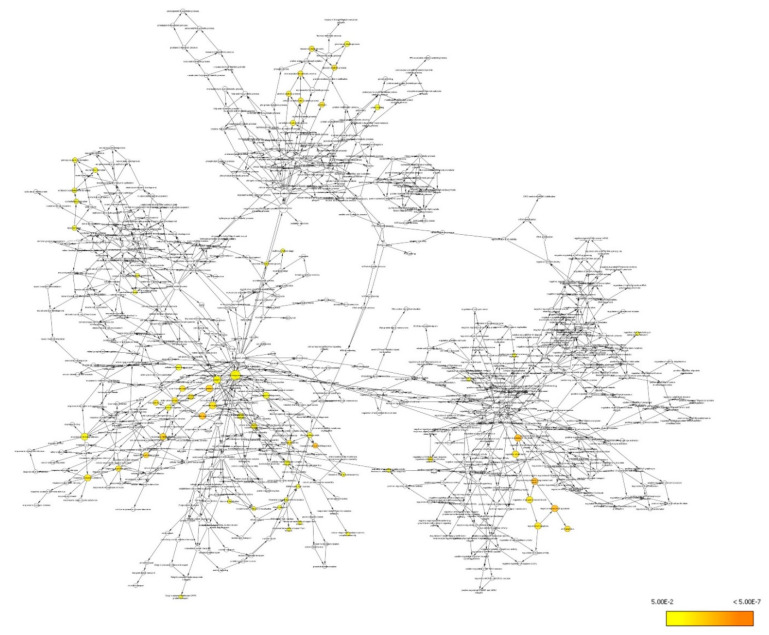
BiNGO results for biological process, as visualized in Cytoscape (organism: *Homo sapiens*). Colored nodes indicate significant overrepresention. White nodes indicate insignificant overrepresention; they are included to show the colored nodes in the context of the GO hierarchy. The color key on the bottom right indicates the significance level of overrepresentation.

These molecular functions were found to involve a myriad of biological processes encompassing negative regulation of apoptosis (GO:43066), negative regulation of programmed cell death (GO:43069), negative regulation of cell death (GO:60548), ribosomal large subunit biogenesis (GO:42273), cytoskeleton organization (GO:7010), response to unfolded protein (GO:6986), multi-organism process (GO:51704), response to biotic stimulus (GO:9607), antiapoptosis (GO:6916), and response to protein stimulus (GO:51789) (Figure 9).

**Figure 10:**
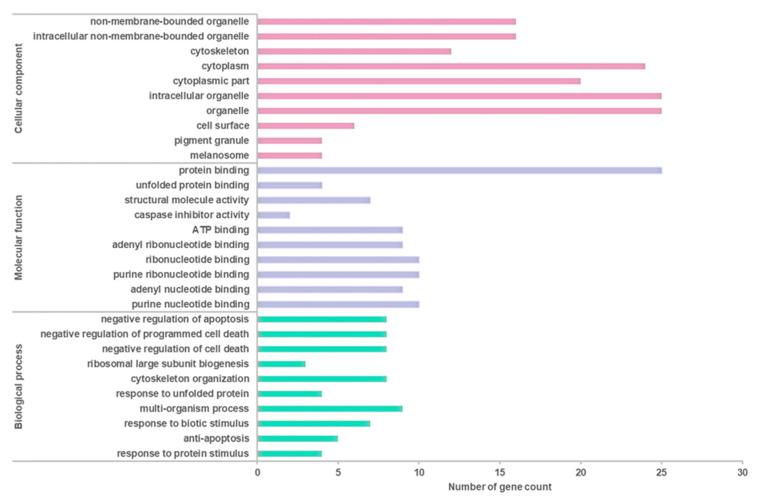
Classification of the top 10 enriched terms in cellular component, molecular function, and biological process annotated by BiNGO (organism: *Homo sapiens*; Pair B, H_2_O_2_ vs. Treatment). Hypergeometric test with Benjamini and Hochberg’s false discovery rate (FDR) correction at *p* < 0.05.

The top ten enriched terms in all three categories were selected to elucidate the association between OSLP protection and H_2_O_2_ stress (Figure 10).

#### 3.4.3. Systematic Pathway Enrichment Analysis

**Figure 11:**
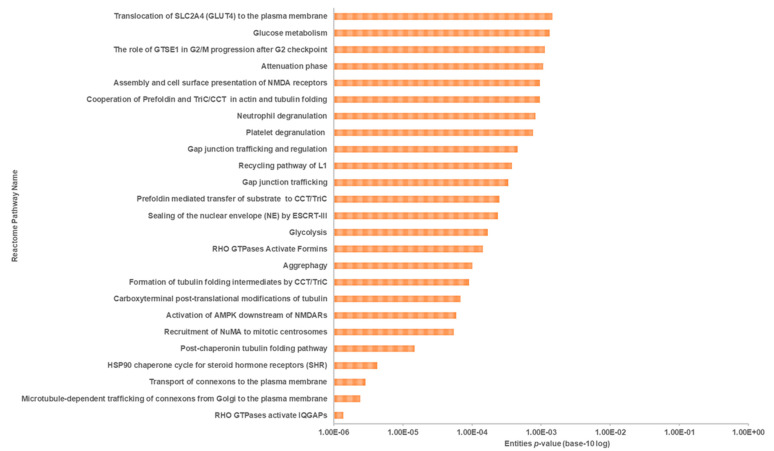
Classification of the 25 most relevant pathways sorted by false discovery rate (FDR) correction at *p* < 0.05 in the logarithmic scale (base 10) generated by the Reactome Pathway Browser (organism: *Homo sapiens*; Pair A, H_2_O_2_ vs. NC).

Reactome Pathways found that the differentially expressed proteins were significantly associated with the 25 pathways with the highest relevance (*p* < 0.05, Figure 11) out of the 80 identified pathways (see supplementary data, Pair A). These pathways were associated with 11 top-level pathway hierarchies, namely, signal transduction, vesicle-mediated transport, cellular responses to external stimuli, metabolism of proteins, cell cycle, neuronal system, autophagy, metabolism, developmental biology, hemostasis, and immune system (Table 5). At sub-level pathway hierarchy, they were seen to be involved in the signaling by Rho GTPases membrane trafficking, cellular responses to stress and HSF1-dependent transactivation, protein folding and post-translational protein modification, mitotic cell cycle, post-NMDA receptor activation events, activation of NMDA receptors, postsynaptic events, macroautophagy, metabolism of glucose and carbohydrates, nervous system development, response to elevated platelet cytosolic Ca^2+^, and the innate immune system (Table 5).

**Figure 12:**
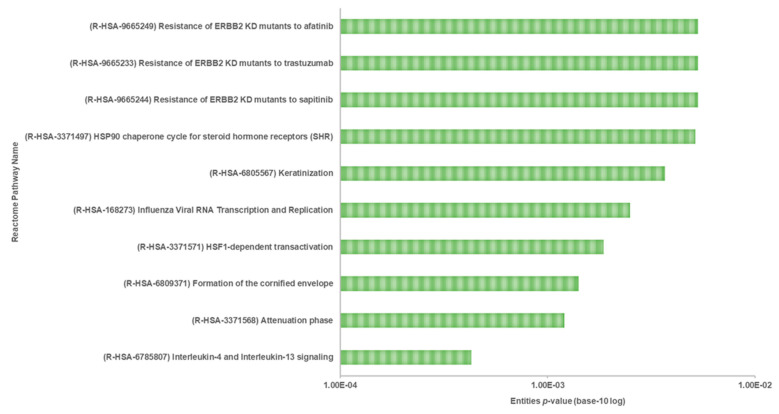
Classification of the 10 most relevant pathways sorted by false discovery rate (FDR) correction at *p* < 0.05 on the logarithmic scale (base 10) generated by the Reactome Pathway Browser (organism: *Homo sapiens*; Pair B, H_2_O_2_ vs. Treatment).

In particular, to predict the protective mechanism of OSLP against H_2_O_2_ stress, the differentially expressed proteins in Pair B (H_2_O_2_ vs. OSLP treatment) were analyzed exclusively by Reactome Pathways. As per the analysis, the protein expression had a significant association with the 10 most relevant pathways (*p* < 0.05, Figure 12) out of the 56 pathways identified (see supplementary data, Pair B). They were interleukin-4 and interleukin-13 signaling (R-HSA-6785807), attenuation phase (R-HSA-3371568), formation of the cornified envelope (R-HSA-6809371), HSF1-dependent transactivation (R-HSA-3371571), HSP90 chaperone cycle for steroid hormone receptors (R-HSA-3371497), keratinization (R-HSA-6805567), influenza viral RNA transcription and replication (R-HSA-168273), resistance of ERBB2 KD mutants to sapitinib (R-HSA-9665244), resistance of ERBB2 KD mutants to trastuzumab (R-HSA-9665233), and resistance of ERBB2 KD mutants to afatinib (R-HSA-9665249). These pathways were associated with three top-level pathway hierarchies, encompassing the immune system, cellular responses to external stimuli, and developmental biology, and two disease pathways, namely, influenza infection and diseases of signal transduction by growth factor receptors and second messengers (Table 6).

**Figure 13:**
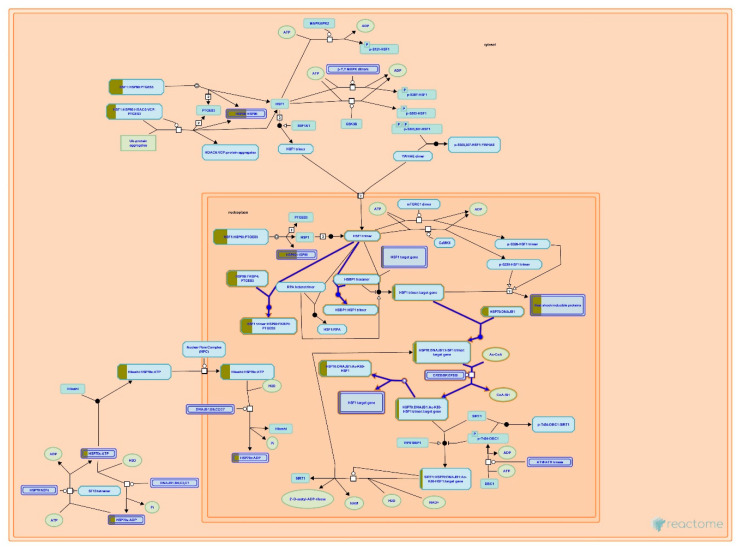
HSP90AA1 (also known as heat shock protein 90) and HSPA8 (also known as heat shock protein family A (Hsp70) member 8 or HSP70), highlighted in yellow, were mapped onto the attenuation phase pathway sorted by false discovery rate (FDR) correction at *p* < 0.05 on the logarithmic scale (base 10) generated by the Reactome Pathway Browser (organism: *Homo sapiens*).

**Figure 14:**
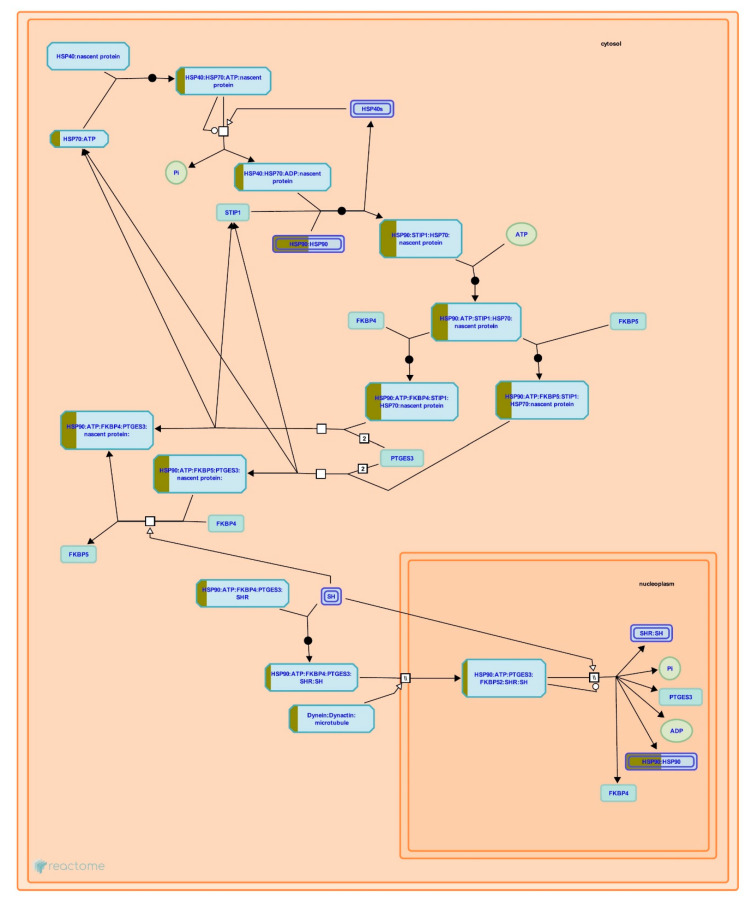
HSP90AA1 (also known as heat shock protein 90) and HSPA8 (also known as heat shock protein family A (Hsp70) member 8 or HSP70), highlighted in yellow, were mapped onto the HSP90 chaperone cycle for steroid hormone receptors (SHRs) pathway, sorted by false discovery rate (FDR) correction at *p* < 0.05 on the logarithmic scale (base 10) generated by the Reactome Pathway Browser (organism: *Homo sapiens*).

**Figure 15:**
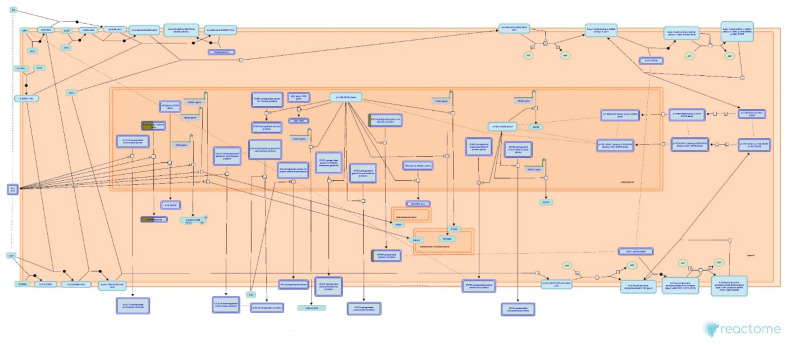
HSPA8 (also known as heat shock protein family A (Hsp70) member 8 or HSP70) and HSP90AA1 (also known as heat shock protein 90, as part of STAT3-upregulated genes for cytosolic proteins and STAT3-upregulated cytosolic proteins), highlighted in yellow, were mapped onto the signaling of interleukin-4 and interleukin-13 pathway, sorted by false discovery rate (FDR) correction at *p* < 0.05 on the logarithmic scale (base 10) generated by the Reactome Pathway Browser (organism: *Homo sapiens*).

Reactome is a database of reactions, pathways, and biological processes. It provides a graphical map showing signaling and metabolic molecules and their relationships. It is also an interactive interface that gives detailed information on components and their relationships to support data visualization, interpretation, and analysis (https://reactome.org/what-is-reactome dated 13th March 2020). Figure 13 and Figure 14 show the two pathways, namely, attenuation phase (R-HSA-3371568) and HSP90 chaperone cycle for steroid hormone receptors (R-HSA-3371497), acting on cellular responses to stress. They were found in both Pairs A and B. Exclusively, Reactome Pathways has predicted interleukin-4 and interleukin-13 signaling (R-HSA-6785807) as the most relevant pathway in Pair B (Figure 15).

## 4. Discussion

Evaluation of the cytotoxic effects of OSLPs on SH-SY5Y cells (24 and 48 h) in this study found that OSLP at concentrations of 25, 50, 125, 250, 500, and 1000 µg/mL did not challenge the survival of SH-SY5Y cells. Therefore, OSLP (25, 50, 125, 250, 500, and 1000) is considered safe for SH-SY5Y cells. In addition, the MNTD and MTD of OSLP at 24 h treatment were determined as 2000 and 4000 µg/mL, respectively. In contrast, the MNTD and MTD of OSLP at 48 h treatment were determined as 1000 and 2000 µg/mL, respectively. MNTD (the maximal non-toxic dose) represents the highest concentration that does not cause cytotoxic effects in a treated cell population, whilst the MTD (the minimal toxic dose) represents the lowest concentration that causes cytotoxic effects in a treated cell population [28]. On top of that, OSLP at 10 mg/mL has been found in this study to be potentially cytotoxic to SH-SY5Y cells. Based on these findings, OSLP (25, 50, 125, 250, 500, and 1000) is used in the evaluation of OSLP-protective effects on SH-SY5Y cells.

Hydrogen peroxide (H_2_O_2_) induction challenged the survival of SH-SY5Y cells. SH-SY5Y cell survival decreased when H_2_O_2_ concentrations increased. H_2_O_2_ at about 150 μM sufficiently inhibited the cell population by half. Concentrations higher than 250 μM were found to sufficiently inhibit the cell population by close to 90%. Based on these findings, the IC_50_ in this study was determined at 150 μM, whereas the IC_90_ was 250 μM and above. The half-maximal inhibitory concentration (IC_50_) represents the dose that inhibits a cell population by half, while the maximal inhibitory concentration (IC_90_) represents the dose that inhibits a cell population by 90% [29]. Therefore, 150 μM of H_2_O_2_ is used in the following evaluation of the protective effects of OSLP on SH-SY5Y cells.

The protective effects of OSLP were evaluated in H_2_O_2_-induced SH-SY5Y cells. H_2_O_2_ induction (150 µM) challenged the survival of SH-SY5Y cells. OSLP treatments exhibited protection against H_2_O_2_ induction in a concentration-dependent manner. OSLP at 125 µg/mL was found to be the lowest treatment dose showing protection against H_2_O_2_ stress. Pre-treatment with 125 µg/mL of OSLP increased the survival of SH-SY5Y cells (by about 30%) compared to the H_2_O_2_ group, although it did not attain statistical significance. Pre-treatments with OSLP at these three concentrations, 250, 500, and 1000 µg/mL, significantly increased the survival of SH-SY5Y cells, with an increase of 39%, 51%, and 57%, respectively, compared to the H_2_O_2_ group. In particular, pre-treatments with 500 and 1000 µg/mL of OSLP demonstrated apparent inhibitions of H_2_O_2_. Such observations suggest that OSLP at these concentrations (250 µg/mL or higher) could potentially inhibit the actions of H_2_O_2_ and, additionally, could promote the growth of SH-SY5Y cells. In line with the bright-field microscopic images obtained, OSLP pre-treatments at 250, 500, and 1000 µg/mL have seen improvements in cell architecture. OSLP-treated H_2_O_2_-induced SH-SY5Y cells showed reduced clumping and shrinkage (i.e., round up), with apparent neurite formations and pyramidal-shaped cells. In contrast, H_2_O_2_-treated cells showed shrinkage, round up, and clumping, all of which are indicative of unhealthy cell appearance, loss of cell viability, and progression towards death [20,30,31,32,33]. Additionally, H_2_O_2_-treated cells showed a decline in the population; in contrast, OSLP pre-treatments showed an increase in the cell population.

Taken together, the outcomes of in vitro assays collectively suggest that OSLP (250, 500, and 1000 µg/mL) could have neuroprotective potential with considerably low cytotoxic effects.

Proteomic analysis has identified a distinct protein expression pattern, where all the proteins are highly expressed in H_2_O_2_ (SH-SY5Y cells induced by 150 µM H_2_O_2_) compared to NC (SH-SY5Y cells without H_2_O_2_ induction and OSLP treatment). This observation is not seen in the OSLP-treated SH-SY5Y cells, with the majority of proteins expressed at lower levels compared to the H_2_O_2_-treated samples. Using functional annotation analysis, the top ten enriched terms in cellular components, molecular functions, and biological processes were identified (Figure 10). The ten selected enriched terms were significant associated with 25 cellular signaling pathways, as suggested by a Reactome Pathways analysis (Figure 11 and Table 5). Additionally, the Reactome Pathways analysis predicted the top ten cellular signaling pathways most likely modulated by OSLP treatment (Figure 12 and Table 6).

In the SH-SY5Y cells, H_2_O_2_ induction could have triggered cellular stress signaling via two main pathways: “attenuation phase” and “HSP90 chaperone cycle for steroid hormone receptors” (Figure 13 and Figure 14). The modulations of these pathways are particularly related to two major heat shock proteins, HSPA8 (also known as heat shock protein family A (Hsp70) member 8 or HSP70) and HSP90AA1 (also known as heat shock protein 90), act together as machinery to modulate the folding of proteins. Studies have shown that most cellular proteins do not activate the HSP90/HSP70-based chaperone machinery for folding, stabilization, and trafficking under normal physiological conditions; following stress, the function of HSP90/HSP70-based chaperone machinery is disrupted [34,35,36]. The HSP90/HSP70-based chaperone machinery can influence a wide variety of client proteins and, thus, affect numerous important cellular pathways, such as protein conformational cycles, co-chaperone interactions, inter-domain communications, protein conformational stability, trafficking and turnover; signal transduction, intracellular transport [34,35,37,38], synaptic transmissions [39,40,41,42], and inflammation [36,43]. Additionally, studies have shown that activations of HSP70 and HSPB1 (also known as HSP27), following exposure to stress, manipulate the heat shock transcriptional response and its client proteins; under normal physiological conditions, these ATP-independent chaperones (HSP70 and HSPB1) provide a wide variety of protections. To name a few, these chaperones prevent the accumulation of improperly folded proteins, participate in the regulated degradation of misfolded proteins, protect the cytoskeleton, are involved in cellular metabolism, and decrease stress-induced apoptosis [44,45,46] in addition to preventing synaptic loss and neuronal death [47].

In this study, HSP90, HSP70, and HSPB1 had higher expressions in the H_2_O_2_ control (induced by H_2_O_2_ alone) compared to the normal control (without H_2_O_2_ induction). Therefore, it is suggested that both impaired the HSP90/HSP70-based chaperone machinery and that HSPB1 activation could have altered, direct or indirectly, a variety of cellular processes in the neuronal cells. In particular, these alterations include neuronal regulation in terms of growth, development, and death; neuronal architecture of cytoskeletons, cytoskeletal dynamics, and cytoskeletal protein expressions [34,35,36]; excitatory postsynaptic transmission activated by NMDA receptors; cellular metabolism, especially glucose and proteins; protein conformations; stabilization and post-translational modifications, as well as inflammatory responses (Figure 11 and Table 5) [44,45,46]. Alterations, as such, are some common themes found in neurodegenerative diseases and neurological disorders.

In the SH-SY5Y cells, OSLP treatment might help buffer against cellular stress signaling chiefly via the “signaling of interleukin-4 and interleukin-13” (IL-4/-13 signaling, R-HSA-6785807) pathway (Figure 15). Within the CNS, HSPs are released from stressed or damaged cells, and they act as local “danger signals” that trigger inflammatory responses. OSLP might modulate the expression of IL-4/IL-13 by affecting the interaction of HSP90, with downstream targets such as HSP8 and the cytoplasmic protein arachidonate 15-lipoxygenase (ALOX15). In the expression of IL-4/-13, HSP90 is one of the genes for cytoplasmic proteins upregulated by signal transducer and activator of transcription 3 (STAT3). Via phosphorylation of STAT3 and signal transducer and activator of transcription 6 (STAT6), HSP8 participates in the downregulation of extracellular proinflammatory signal transducers, including ALOX15. Most likely, by modulating the “IL-4/-13 signaling” pathway, OSLP promotes the neuroprotective effects of IL-4 and IL-13, acting as anti-inflammatory cytokines [48,49], or IL-4 alone acts directly as a cytoprotective cytokine [50]. For instance, IL-4 and IL-13 induce the alternative activation of microglia (also known as the M2 state) to protect against neuronal damage in the hippocampus and the cortex in experimental models of ischemic stress [51,52]. Specifically, IL-13 alone has shown anti-inflammatory ability in a mouse model of cerebral ischemia [53]. In contrast, a study on humans with multiple sclerosis found high levels of IL-13-enhanced gamma-aminobutyric acid (GABA, the dominant inhibitory neurotransmitter) over glutamate transmission [54]. Otherwise, low levels of IL-4 in epileptic patients have been shown to decrease inflammation-related epilepsy [55,56].

Additionally, OSLP treatment might also protect against cellular-stress-mediated pathways, including “attenuation phase” (R-HSA-3371568) and “HSP90 chaperone cycle for steroid hormone receptors” (R-HSA-3371497) pathways (Figure 13 and Figure 14). Via the “attenuation phase” pathway, OSLP might modulate the downstream interaction of HSP70 and its co-chaperone HSP40 with CoREST (transcriptional corepressor for repressor element 1-silencing transcription factor) at the negative-feedback loop. This negative feedback loop provides an important mechanism by which cells can regulate the activation and attenuation of heat shock factor 1 (HSF1) via the presence and concentration of HSPs in the cell. OSLP might also regulate SHR–protein interactions via the “HSP90 chaperone cycle for steroid hormone receptors” pathway. Upon the upstream activations of HSP40, HSP70, and stress-induced-phosphoprotein 1 (STIP1), respectively, HSP90 binds to the downstream co-chaperones FK506 binding protein 5 (FKBP51 and FKBP52) and prostaglandin E synthase 3 (PTGES3). The HSP90 and chaperone-mediated conformational changes are required to keep SHRs in a ligand-binding-competent state. In this regard, OSLP could have promoted the cytoprotective functions of HSPs as an alternative to neuroprotection [57]. For instance, HSPs and their respective co-chaperones facilitate native protein stabilization, translocation, re-folding, and degradation in response to stressful stimuli. HSP-based chaperone machinery not only ensures protein quality control but also prevents protein aggregation that would otherwise overwhelm the cell and lead to programmed cell death (apoptosis) or necrosis in unfavorable conditions [58,59]. In recent times, HSPs have demonstrated their ability to fine-tune inflammation in the CNS [43]. For instance, HSPs have been shown to assist in the protection of motor neurons and to prevent chronic inflammation after spinal cord injuries in animal models [60,61].

Last but not least, the changes in both KRT8 and KRT19 are also worthy of mention. They are keratins; KRT8 is a member of the type II keratin family, and KRT19 belongs to the type I family. The intermediate filament (IF) cytoskeleton of all epithelia is built from type I and type II keratins. Keratins not only maintain structural rigidity and stability, they also provide resistance to environmental stress [62]. In the presence of H_2_O_2_ stress, the keratin network organization in the cytoskeleton can be altered. The altered expression of keratins has an impact on the keratin network organization and has been associated with inflammation, cellular stress, epithelial barrier defects, and higher sensitivity to tumor necrosis factor (TNF)-induced cell death [63,64,65].

Taken together, the protein expression study and bioinformatics analysis collectively suggest that OSLP could protect neuronal cells against inflammation and cellular stress. The neuroprotective potential of OSLP can be attributed to an assortment of proteins present in the crude. For instance, baicalein 7-O-glucuronosyltransferase and its glucoronosylated baicalein have been reported to possess anti-inflammatory, antioxidative, and neuroprotective [66] as well as anticonvulsive activities [67]; baicalin biosynthesized by baicalin-beta-D-glucuronidase has shown antioxidant activity [68,69]; rosmarinic acid biosynthesized by rosmarinate synthase have attracted interest for being anti-inflammatory, antioxidant, antiangiogenic, antitumor, antimicrobial [70] and antiseizure [71].

## 5. Concluding Remarks

The study suggests that OSLP could be a potential neuroprotective agent. Its neuroprotective potential is attributed to the ability of OSLP to modulate the “signaling of interleukin-4 and interleukin-13” pathway as the predominant mode of action and, thereby, activate anti-inflammatory cytokines to protect against proinflammatory responses under stress conditions. OSLP also modulates the “attenuation phase” and “HSP90 chaperone cycle for steroid hormone receptors” pathways to counteract HSP-induced damage under stress conditions. OSLP is, therefore, worthy of detailed investigations.

## Figures and Tables

**Table 1:** Experiment groups in the evaluation of OSLP protective effects on SH-SY5Y cells.

Group	Treatment
NC	Normal control (untreated cells)
H_2_O_2_	H_2_O_2_ induction (150 µM H_2_O_2_)
25	OSLP 25 µg/mL + 150 µM H_2_O_2_
50	OSLP 50 µg/mL + 150 µM H_2_O_2_
125	OSLP 125 µg/mL + 150 µM H_2_O_2_
250	OSLP 250 µg/mL + 150 µM H_2_O_2_
500	OSLP 500 µg/mL + 150 µM H_2_O_2_
1000	OSLP 1000 µg/mL + 150 µM H_2_O_2_

Remark: H_2_O_2_, hydrogen peroxide; OSLP, *Orthosiphon stamineus* leaf protein.

**Table 2:** Experiment groups in the protein expression study.

Group	Treatment
NC	Normal control (untreated cells)
H_2_O_2_	H_2_O_2_ induction (150 µM H_2_O_2_)
250	OSLP 250 µg/mL + 150 µM H_2_O_2_
500	OSLP 500 µg/mL + 150 µM H_2_O_2_
1000	OSLP 1000 µg/mL + 150 µM H_2_O_2_

Remark: H_2_O_2_, hydrogen peroxide; OSLP, *Orthosiphon stamineus* leaf protein.

**Table 3:** Differentially expressed proteins identified from Pair A (H_2_O_2_ vs. NC).

Uniprot Accession ID	Uniprot Protein Name	Significance (≥13)	Coverage (%)	#Peptides	#Unique	Avg. Mass	Group Profile (Ratio of NC/H_2_O_2_)	Ensembl Protein
P11142	Heat shock cognate 71 kDa protein	34.31	16	7	5	67,980	0.34:1.00	HSPA8
P04075	Fructose-bisphosphate aldolase A	24.61	25	5	5	39,818	0.20:1.00	ALDOA
P68371	Tubulin beta-4B chain	24.00	15	5	1	49,831	0.12:1.00	TUBB4B
P05787	Keratin, type II cytoskeletal 8	23.26	63	31	15	53,704	0.20:1.00	KRT8
O00299	Chloride intracellular channel protein 1	23.2	8	1	1	26,794	0.02:1.00	CLIC1
P06733	Alpha-enolase	22.22	23	7	7	47,169	0.28:1.00	ENO1
P05783	Keratin, type I cytoskeletal 18	20.41	63	19	17	48,030	0.16:1.00	KRT18
P38646	Stress-70 protein, mitochondrial	20.12	13	6	6	72,401	0.20:1.00	HSPA9
P04792	Heat shock protein beta-1	19.50	40	6	6	22,783	0.15:1.00	HSPB1
P23528	Cofilin-1	18.80	28	4	4	22,728	0.17:1.00	CFL1
P07737	Profilin-1	18.10	46	5	5	15,054	0.25:1.00	PFN1
P14618	Pyruvate kinase PKM	17.35	18	6	3	57,937	0.23:1.00	PKM/PK3
P30041	Peroxiredoxin-6	16.95	21	1	1	11,161	0.18:1.00	PRDX6
P22314	Ubiquitin-like modifier-activating enzyme 1	16.61	3	2	2	117,849	0.10:1.00	UBA1
P63261	Actin, cytoplasmic 2	16.55	33	11	1	41,793	0.07:1.00	ACTG1
P49327	Fatty acid synthase	16.33	3	4	4	273,424	0.29:1.00	FASN
Q9BQE3	Tubulin alpha-1C chain	15.94	18	7	7	57,730	0.26:1.00	TUBA1C
P14174	Macrophage migration inhibitory factor	14.39	10	1	1	12,476	0.11:1.00	MIF
P08727	Keratin, type I cytoskeletal 19	13.91	34	10	8	44,106	0.15:1.00	KRT19
P07900	Heat shock protein HSP 90-alpha	13.79	12	6	3	68,372	0.36:1.00	HSP90AA1
P11021	Endoplasmic reticulum chaperone BiP	13.35	7	3	2	66,914	0.17:1.00	HSPA5
P06748	Nucleophosmin	13.27	15	2	2	28,400	0.21:1.00	NPM1

Remark: The Ensembl Human Database (https://asia.ensembl.org/Homo_sapiens/Info/Index, accessed on 9 November 2019) was used to search for the Ensembl protein nomenclatures.

**Table 4:** Differentially expressed proteins identified from Pair B (H_2_O_2_ vs. Treatment).

Uniprot Accession ID	Uniprot Protein Name	Significance (≥13)	Coverage (%)	#Peptides	#Unique	Avg. Mass	Group Profile (Ratio of H_2_O_2_/Treatment)	Ensembl Protein
Q,	Heterogeneous nuclear ribonucleoprotein U	31.91	2	1	1	67,980	1.00:1.72	HNRNPU
P05787	Keratin, type II cytoskeletal 8	25.07	69	41	20	39,818	1.00:0.44	KRT8
P11142	Heat shock cognate 71 kDa protein	18.72	21	11	2	49,831	1.00:0.94	HSPA8
P83731	60S ribosomal protein L24	16.92	11	1	1	53,704	1.00:2.79	RPL24
P50914	60S ribosomal protein L14	16.33	6	1	1	26,794	1.00:0.58	RPL14
P16949	Stathmin	15.88	15	2	2	47,169	1.00:2.24	STMN1
Q6UWU2	Beta-galactosidase-1-like protein	15.63	2	1	1	48,030	1.00:0.38	GLB1L
P08727	Keratin, type I cytoskeletal 19	14.96	43	14	12	72,401	1.00:0.27	KRT19
P12277	Creatine kinase B-type	14.25	12	2	2	22,783	1.00:0.41	CKB
P07900	Heat shock protein HSP 90-alpha	13.00	20	11	3	22,728	1.00:0.49	HSP90AA1

Remark: The Ensembl Human Database (https://asia.ensembl.org/Homo_sapiens/Info/Index, accessed on 9 November 2018) was used to search for the Ensembl protein nomenclatures.

**Table 5:** Pathway hierarchy of the 25 most relevant pathways. Bold font indicates the top-level pathway hierarchy; bold and italic font indicates the sub-pathway hierarchy.

Reactome Pathway Name	Reactome Pathway Identifier	Entities *p*-Value
**Signal transduction**		
**Signaling by Rho GTPases**		
RHO GTPases activate IQGAPs	R-HSA-5626467	1.38 × 10^−6^
RHO GTPases activate formins	R-HSA-5663220	1.45 × 10^−4^
**Vesicle-mediated transport**		
**Membrane trafficking**		
Microtubule-dependent trafficking of connexons from Golgi to the plasma membrane	R-HSA-190840	2.41 × 10^−6^
Transport of connexons to the plasma membrane	R-HSA-190872	2.87 × 10^−6^
Gap junction trafficking	R-HSA-190828	3.36 × 10^−4^
Gap junction trafficking and regulation	R-HSA-157858	4.56 × 10^−4^
Translocation of SLC2A4 (GLUT4) to the plasma membrane	R-HSA-1445148	0.001455
**Cellular responses to external stimuli**		
**Cellular responses to stress**		
HSP90 chaperone cycle for steroid hormone receptors (SHRs)	R-HSA-3371497	4.24 × 10^−6^
Attenuation phase	R-HSA-3371568	0.001068
**Metabolism of proteins**		
**Protein folding**		
Post-chaperonin tubulin folding pathway	R-HSA-389977	1.48 × 10^−5^
Formation of tubulin folding intermediates by CCT/TriC	R-HSA-389960	9.10 × 10^−5^
Prefoldin mediated transfer of substrate to CCT/TriC	R-HSA-389957	2.49 × 10^−4^
Cooperation of Prefoldin and TriC/CCT in actin and tubulin folding	R-HSA-389958	9.66 × 10^−4^
***Post-translational protein modification***		
Carboxyterminal post-translational modifications of tubulin	R-HSA-8955332	6.85 × 10^−5^
**Cell cycle**		
***Cell cycle, mitotic***		
Recruitment of NuMA to mitotic centrosomes	R-HSA-380320	5.42 × 10^−5^
Sealing of the nuclear envelope (NE) by ESCRT-III	R-HSA-9668328	2.37 × 10^−4^
The role of GTSE1 in G2/M progression after G2 checkpoint	R-HSA-8852276	0.001135
**Neuronal system**		
***Post N-methyl-D-aspartate (NMDA) receptor activation events***		
Activation of AMPK downstream of NMDARs	R-HSA-9619483	5.89 × 10^−5^
***Activation of NMDA receptors and postsynaptic events***		
Assembly and cell surface presentation of NMDA receptors	R-HSA-9609736	9.66 × 10^−4^
**Autophagy**		
***Macroautophagy***		
Aggrephagy	R-HSA-9646399	1.02 × 10^−4^
**Metabolism**		
***Glucose metabolism***		
Glycolysis	R-HSA-70171	1.70 × 10^−4^
***Metabolism of Carbohydrates***	R-HSA-70326	0.001332
Glucose metabolism		
**Developmental biology**		
***Nervous system development***		
Recycling pathway of L1	R-HSA-437239	3.81 × 10^−4^
**Haemostasis**		
***Response to elevated platelet cytosolic Ca^2+^***		
Platelet degranulation	R-HSA-114608	7.68 × 10^−4^
**Immune system**		
***Innate immune system***		
Neutrophil degranulation	R-HSA-6798695	8.23 × 10^−4^

**Table 6:** Pathway hierarchy of the 10 most relevant pathways. Bold font indicates the top-level pathway hierarchy; bold and italic font indicates the sub-pathway hierarchy.

Reactome Pathway Name	Reactome Pathway Identifier	Entities *p*-Value
**Immune system**		
**Cytokine signaling in immune system**		
Interleukin-4 and Interleukin-13 signaling	R-HSA-6785807	4.29 × 10^−4^
**Cellular responses to external stimuli**		
**Cellular responses to stress**		
HSF1-dependent transactivation	R-HSA-3371571	0.001867
Attenuation phase	R-HSA-3371568	0.001204
HSP90 chaperone cycle for steroid hormone receptors (SHRs)	R-HSA-3371497	0.005152
**Developmental biology**		
**Keratinisation**		
Formation of the cornified envelope	R-HSA-6809371	0.001410
Keratinisation	R-HSA-6805567	0.003672
**Disease**		
**Influenza infection**		
Influenza Viral RNA Transcription and Replication	R-HSA-168273	0.002496
**Diseases of signal transduction by growth factor receptors & second messengers**		
Resistance of ERBB2 KD mutants to sapitinib	R-HSA-9665244	0.0053
Resistance of ERBB2 KD mutants to trastuzumab	R-HSA-9665233	0.0053
Resistance of ERBB2 KD mutants to afatinib	R-HSA-9665249	0.0053

## Data Availability

Data is contained within the article or supplementary material The data presented in this study are available in S1.

## Supplementary Materials

The following are available online at https://www.mdpi.com/article/10.3390/life11060585/s1, Table S1: List of the total identified protein compositions of OSLP (both in-solution and in-gel digestions) using shotgun-ESI-LC-MS/MS approach. The annotations were retrieved from the databases of UniProtKB (http://www.uniprot.org/uniprot/, accessed on 19 June 2021) and NCBInr (https://www.ncbi.nlm.nih.gov/, accessed on 19 June 2021).
