# Supplementary material for: Orthosiphon stamineus Proteins Alleviate Hydrogen Peroxide Stress in SH-SY5Y Cells
Source: Life (Basel). 2021 Jun 20;11(6):585. doi: 10.3390/life11060585 (PMC8235403; doi:10.3390/life11060585)
Supplement: Supplementary file 1 [file life-11-00585-s001.zip › life-1234951-supplementary.pdf]

SUPPLEMENTARY DATA

Table SD-1. List of the total identified protein compositions of OSLP (both in-solution and in-gel digestions) using shotgun-ESI-LC-MS/MS approach. The annotations were retrieved from the databases of UniProtKB (http://www.uniprot.org/uniprot/) and NCBInr (https://www.ncbi.nlm.nih.gov/).

No.	Accession Number	Average Mass	Max-10logP	Max Coverage (%)	Max# Peptides	Max# Unique	Description
1	gi|403399409|sp|E2E2P0.1|GTPS_ORIVU	69063	95.94	7	6	6	Gamma-terpinene synthase, chloroplastic; Short=Ovtps2; AltName: Full=Alpha-terpinene synthase; Flags: Precursor
2	gi|75306222|sp|Q947B7.1|MFS_MENPI	55360	85.61	4	2	2	(+)-menthofuran synthase; AltName: Full=(+)-pulegone 9-hydroxylase
3	gi|75251483|sp|Q5SBP6.1|GDS_OCIBA	63395	77.82	2	1	1	Germacrene-D synthase; AltName: Full=(-)-germacrene D synthase
4	gi|29839421|sp|Q9XGW0.1|COMT1_OCIBA	39529	73.20	2	1	1	Caffeic acid 3-O-methyltransferase 1; Short=CAOMT-1; Short=COMT-1; AltName: Full=S-adenosysl-L-methionine:caffeic acid 3-O-methyltransferase 1
5	gi|29839420|sp|Q9XGV9.1|COMT2_OCIBA	39613	71.77	2	1	1	Caffeic acid 3-O-methyltransferase 2; Short=CAOMT-2; Short=COMT-2; AltName: Full=S-adenosysl-L-methionine:caffeic acid 3-O-methyltransferase 2
6	gi|75315260|sp|Q9XHE7.1|C71DD_MENPI	56601	72.69	2	1	1	Cytochrome P450 71D13; AltName: Full=(-)-(4S)-Limonene-3-hydroxylase; AltName: Full=Cytochrome P450 isoform PM17
7	gi|75129878|sp|Q6WAU0.1|PULR_MENPI	37915	68.99	6	3	3	(+)-pulegone reductase
8	gi|75251484|sp|Q5SBP7.1|SELS_OCIBA	63125	68.51	3	2	2	Selinene synthase
9	gi|75251481|sp|Q5SBP4.1|AZIS_OCIBA	62858	67.78	4	2	2	Alpha-zingiberene synthase
10	gi|5915814|sp|O04164.1|C71A6_NEPRA	57955	66.16	2	1	1	Cytochrome P450 71A6
11	gi|62899675|sp|O81192.1|BPPS_SALOF	69292	49.27	2	1	1	(+)-bornyl diphosphate synthase, chloroplastic; Short=BPPS; AltName: Full=(+)-alpha-pinene synthase; AltName: Full=(+)-camphene synthase; AltName: Full=SBS; Flags: Precursor

No.	Accession Number	Average Mass	Max-10logP	Max Coverage (%)	Max# Peptides	Max# Unique	Description
12	gi|122210943|sp|Q2XSC5.1|LALIN_LAVAN	65654	47.80	2	1	1	R-linalool synthase; Short=LaLINS
13	gi|75180331|sp|Q9LRC8.1|BAGLU_SCUBA	58772	46.95	1	1	1	Baicalin-beta-D-glucuronidase; AltName: Full=Baicalinase; Flags: Precursor
14	gi|122233627|sp|Q4JF75.1|RBR_SCUBA	111795	39.51	1	1	1	Retinoblastoma-related protein
15	gi|75251477|sp|Q5SBP0.1|TPSD_OCIBA	70000	39.42	1	1	1	Terpinolene synthase, chloroplastic; Flags: Precursor
16	gi|5921781|sp|O04111.1|CHSY_PERFR	42686	36.23	3	1	1	Chalcone synthase; AltName: Full=Naringenin-chalcone synthase
17	gi|75251482|sp|Q5SBP5.1|GCS1_OCIBA	63566	26.10	1	1	1	Gamma-cadinene synthase; AltName: Full=(+)-gamma-cadinene synthase
18	gi|910312590|ref|YP_009162251.1|	158800	37.85	1	1	1	DNA-directed RNA polymerase beta subunit-2 (chloroplast) [Scutellaria baicalensis]
19	gi|827345829|gb|AKJ77130.1|	158800	37.85	1	1	1	DNA-directed RNA polymerase beta subunit-2 (chloroplast) [Scutellaria baicalensis]
20	gi|916442749|gb|AKZ23931.1|	157974	34.34	1	1	1	RNA polymerase beta'' subunit (plastid) [Salvia nemorosa]
21	gi|836643373|ref|YP_009144505.1|	158291	34.34	1	1	1	DNA-directed RNA polymerase beta'' subunit (chloroplast) [Rosmarinus officinalis]
22	gi|827345133|gb|AKJ76717.1|	158291	34.34	1	1	1	DNA-directed RNA polymerase beta'' subunit (chloroplast) [Rosmarinus officinalis]
23	gi|401879732|gb|AFQ30919.1|	158403	34.34	1	1	1	DNA-directed RNA polymerase beta subunit-2 (chloroplast) [Salvia miltiorrhiza]
24	gi|573461941|emb|CCQ71610.1|	158403	34.34	1	1	1	DNA-directed RNA polymerase beta'' subunit (chloroplast) [Salvia miltiorrhiza]
25	gi|827345865|gb|AKJ77166.1|	25335	24.97	4	1	1	NAD(P)H-quinone oxidoreductase subunit K, chloroplastic [Scutellaria baicalensis]

No.	Accession Number	Average Mass	Max-10logP	Max Coverage (%)	Max# Peptides	Max# Unique	Description
26	gi|910312604|ref|YP_009162265.1|	25335	24.97	4	1	1	NAD(P)H-quinone oxidoreductase subunit K, chloroplastic [Scutellaria baicalensis]
27	gi|75129878|sp|Q6WAU0.1|PULR_MENPI	37915	20.85	2	1	1	(+)-pulegone reductase
28	gi|5915814|sp|O04164.1|C71A6_NEPRA	57955	20.22	2	1	1	Cytochrome P450 71A6
29	gi|122219295|sp|Q49SP7.1|TPSCS_POGCB	63586	136.21	11	7	7	Gamma-curcumene synthase; AltName: Full=PatTpsA
30	gi|5915814|sp|O04164.1|C71A6_NEPRA	57955	113.24	3	4	4	Cytochrome P450 71A6
31	gi|75129878|sp|Q6WAU0.1|PULR_MENPI	37915	91.51	3	2	2	(+)-pulegone reductase
32	gi|403399409|sp|E2E2P0.1|GTPS_ORIVU	69063	86.99	2	1	1	Gamma-terpinene synthase, chloroplastic; Short=Ovtps2; AltName: Full=Alpha-terpinene synthase; Flags: Precursor
33	gi|8134569|sp|Q42662.2|METE_PLESU	84590	83.05	1	1	1	5-methyltetrahydropteroyltriglutamate--homocysteine methyltransferase; AltName: Full=Cobalamin-independent methionine synthase isozyme; AltName: Full=Vitamin-B12-independent methionine synthase isozyme
34	gi|122237148|sp|Q15GI4.1|EGS1_OCIBA	35607	65.94	3	1	1	Eugenol synthase 1 [Ocimum basilicum] (Sweet basil)
35	gi|75227033|sp|Q76MR7.1|UBGAT_SCUBA	48654	63.01	2	1	1	Baicalein 7-O-glucuronosyltransferase; AltName: Full=UDP-glucuronate:baicalein 7-O-glucuronosyltransferase
36	gi|75251483|sp|Q5SBP6.1|GDS_OCIBA	63395	59.78	1	1	1	Germacrene-D synthase; AltName: Full=(-)-germacrene D synthase
37	gi|75161989|sp|Q8W1W9.1|5MAT1_SALSN	50724	56.73	3	1	1	Malonyl-coenzyme:anthocyanin 5-O-glucoside-6'''-O-malonyltransferase; Short=Malonyl CoA:anthocyanin 5-O-glucoside-6'''-O-malonyltransferase; Short=Ss5MaT1
38	gi|84027871|sp|Q93WU2.1|EOMT1_OCIBA	40237	48.94	2	1	1	Eugenol O-methyltransferase; AltName: Full=(Iso)eugenol O-methyltransferase EOMT1;

No.	Accession Number	Average Mass	Max-10logP	Max Coverage (%)	Max# Peptides	Max# Unique	Description
							AltName: Full=S-adenosysl-L-methionine:(Iso)eugenol O-methyltransferase EOMT1
39	gi|75251482|sp|Q5SBP5.1|GCS1_OCIBA	63566	48.14	1	1	1	Gamma-cadinene synthase; AltName: Full=(+)-gamma-cadinene synthase
40	gi|403399735|sp|E2E2N7.1|BCGS_ORIVU	64443	45.36	2	1	1	Bicyclogermacrene synthase; Short=Ovtps4
41	gi|75338882|sp|Q9ZR27.1|5GT1_PERFR	50974	44.00	2	1	1	Anthocyanidin 3-O-glucoside 5-O-glucosyltransferase 1; AltName: Full=UDP-glucose:anthocyanin 5-O-glucosyltransferase 3R4; Short=p3R4; Flags: Precursor
42	gi|75338881|sp|Q9ZR26.1|5GT2_PERFR	49110	37.36	2	1	1	Anthocyanidin 3-O-glucoside 5-O-glucosyltransferase 2; AltName: Full=UDP-glucose:anthocyanin 5-O-glucosyltransferase 3R6; Short=p3R6; Flags: Precursor
43	gi|75180331|sp|Q9LRC8.1|BAGLU_SCUBA	58772	34.79	1	1	1	Baicalin-beta-D-glucuronidase; AltName: Full=Baicalinase; Flags: Precursor
44	gi|75288825|sp|Q65CJ7.2|HPPR_PLESU	34128	29.75	3	1	1	Hydroxyphenylpyruvate reductase; Short=HPPR
45	gi|748013964|gb|AJE28434.1|	225117	108.02	6	15	11	Protein TIC 214 [Premna microphylla]
46	gi|752789846|ref|YP_009117280.1|	225117	108.02	6	15	11	Protein TIC 214 [Premna microphylla]
47	gi|827346602|gb|AKJ77788.1|	181164	97.80	5	7	4	Protein TIC 214 [Perilla frutescens]
48	gi|916442749|gb|AKZ23931.1|	157974	43.03	2	2	2	RNA polymerase beta'' subunit (plastid) [Salvia nemorosa]
49	gi|401879732|gb|AFQ30919.1|	158403	43.03	2	2	2	DNA-directed RNA polymerase subunit beta'' [Salvia miltiorrhiza]
50	gi|836643373|ref|YP_009144505.1|	158291	43.03	2	2	2	DNA-directed RNA polymerase subunit beta'' [Rosmarinus officinalis]
51	gi|827345133|gb|AKJ76717.1|	158291	43.03	2	2	2	DNA-directed RNA polymerase subunit beta'' [Rosmarinus officinalis]

No.	Accession Number	Average Mass	Max-10logP	Max Coverage (%)	Max# Peptides	Max# Unique	Description
52	gi|827345132|gb|AKJ76716.1|	218366	30.45	1	1	1	Protein TIC 214 [Rosmarinus officinalis]
53	gi|836643372|ref|YP_009144573.1|	218366	30.45	1	1	1	Protein TIC 214 [Rosmarinus officinalis]
54	gi|5915814|sp|O04164.1|C71A6_NEPRA	57955	29.66	2	1	1	Cytochrome P450 71A6
55	gi|75129878|sp|Q6WAU0.1|PULR_MENPI	37915	25.41	2	1	1	(+)-pulegone reductase
56	gi|510794432|gb|AGN52182.1|	27098	22.41	3	1	1	MYB-related transcription factor [Salvia miltiorrhiza]
57	gi|510794526|gb|AGN52229.1|	28066	22.41	3	1	1	MYB-related transcription factor [Salvia miltiorrhiza]
58	gi|510794500|gb|AGN52216.1|	35378	22.41	3	1	1	MYB-related transcription factor [Salvia miltiorrhiza]
59	gi|510794478|gb|AGN52205.1|	38440	22.41	2	1	1	MYB-related transcription factor [Salvia miltiorrhiza]
60	gi|844572791|gb|AKN09590.1|	34611	22.35	4	1	1	basic helix-loop-helix transcription factor [Salvia miltiorrhiza]
61	gi|844572722|gb|AKN09568.1|	41656	20.96	2	1	1	basic helix-loop-helix transcription factor [Salvia miltiorrhiza]
62	gi|908373664|gb|AKT44364.1|	81096	21.38	1	1	1	heat shock protein 2 [Tectona grandis]
63	gi|5921781|sp|O04111.1|CHSY_PERFR	42686	131.05	46	35	35	Chalcone synthase; AltName: Full=Naringenin-chalcone synthase
64	gi|75306222|sp|Q947B7.1|MFS_MENPI	55360	96.60	19	10	10	(+)-menthofuran synthase; AltName: Full=(+)-pulegone 9-hydroxylase
65	gi|56749087|sp|Q85XY6.1|MATK_OCIBA	60282	91.89	20	12	12	Maturase K; AltName: Full=Intron maturase
66	gi|122219295|sp|Q49SP7.1|TPSCS_POGCB	63586	91.44	13	6	6	Gamma-curcumene synthase; AltName: Full=PatTpsA
67	gi|75227033|sp|Q76MR7.1|UBGAT_SCUBA	48654	82.13	15	8	8	Baicalein 7-O-glucuronosyltransferase; AltName: Full=UDP-glucuronate:baicalein 7-O-glucuronosyltransferase

No.	Accession Number	Average Mass	Max-10logP	Max Coverage (%)	Max# Peptides	Max# Unique	Description
68	gi|75290511|sp|Q6IV13.1|C7D95_MENSP	56322	73.91	15	7	7	Cytochrome P450 71D95; AltName: Full=(-)-(4S)-Limonene-3-hydroxylase
69	gi|75293242|sp|Q6WKY9.1|C7D95_MENGR	56365	73.91	15	7	7	Cytochrome P450 71D95; AltName: Full=(-)-(4S)-Limonene-3-hydroxylase
70	gi|75251482|sp|Q5SBP5.1|GCS1_OCIBA	63566	64.20	5	3	3	Gamma-cadinene synthase; AltName: Full=(+)-gamma-cadinene synthase
71	gi|62899675|sp|O81192.1|BPPS_SALOF	69292	45.92	2	1	1	(+)-bornyl diphosphate synthase, chloroplastic; Short=BPPS; AltName: Full=(+)-alpha-pinene synthase; AltName: Full=(+)-camphene synthase; AltName: Full=SBS; Flags: Precursor
72	gi|75219538|sp|O48935.1|TPSBF_MENPI	63830	45.64	4	2	2	Beta-farnesene synthase
73	gi|75338881|sp|Q9ZR26.1|5GT2_PERFR	49110	43.65	2	1	1	Anthocyanidin 3-O-glucoside 5-O-glucosyltransferase 2; AltName: Full=UDP-glucose:anthocyanin 5-O-glucosyltransferase 3R6; Short=p3R6; Flags: Precursor
74	gi|75293243|sp|Q6WKZ0.1|C7D94_MENGR	56308	41.18	2	1	1	Cytochrome P450 71D94
75	gi|62900763|sp|O81191.1|SCS_SALOF	69369	40.37	2	1	1	1,8-cineole synthase, chloroplastic; Short=SCS; Flags: Precursor
76	gi|403399409|sp|E2E2P0.1|GTPS_ORIVU	69063	39.33	3	2	2	Gamma-terpinene synthase, chloroplastic; Short=Ovtps2; AltName: Full=Alpha-terpinene synthase; Flags: Precursor
77	gi|403399735|sp|E2E2N7.1|BCGS_ORIVU	64443	37.93	1	1	1	Bicyclogermacrene synthase; Short=Ovtps4
78	gi|75338882|sp|Q9ZR27.1|5GT1_PERFR	50974	37.58	2	1	1	Anthocyanidin 3-O-glucoside 5-O-glucosyltransferase 1; AltName: Full=UDP-glucose:anthocyanin 5-O-glucosyltransferase 3R4; Short=p3R4; Flags: Precursor
79	gi|75251477|sp|Q5SBP0.1|TPSD_OCIBA	70000	37.03	2	1	1	Terpinolene synthase, chloroplastic; Flags: Precursor
80	gi|75251478|sp|Q5SBP1.1|MYRS_OCIBA	69964	37.38	2	1	1	Beta-myrcene synthase, chloroplastic; Flags: Precursor

No.	Accession Number	Average Mass	Max-10logP	Max Coverage (%)	Max# Peptides	Max# Unique	Description
81	gi|75251479|sp|Q5SBP2.1|FES_OCIBA	69866	37.38	2	1	1	(-)-endo-fenchol synthase, chloroplastic; Flags: Precursor
82	gi|75224312|sp|Q6USK1.1|GERS_OCIBA	64933	32.96	2	1	1	Geraniol synthase, chloroplastic; Short=ObGES; Flags: Precursor
83	gi|3914545|sp|Q31655.1|RBL_AJUCH	52455	29.59	2	1	1	Ribulose bisphosphate carboxylase large chain; Short=RuBisCO large subunit; Flags: Precursor
84	gi|548687|sp|P36483.1|RBL_CALDI	49209	29.59	2	1	1	Ribulose bisphosphate carboxylase large chain; Short=RuBisCO large subunit; Flags: Precursor
85	gi|132044|sp|P28453.1|RBL_SCUBO	51794	21.37	2	1	1	Ribulose bisphosphate carboxylase large chain; Short=RuBisCO large subunit
86	gi|122219292|sp|Q49SP4.1|TPGD1_POGCB	64197	24.21	1	1	1	Germacrene D synthase 1; AltName: Full=PatTpsB15
87	gi|510785777|sp|G0LD36.1|RAS_MELOI	47161	21.06	2	1	1	Rosmarinate synthase; Short=MoRAS; AltName: Full=Hydroxycinnamoyl-CoA:hydroxyphenyllactate hydroxycinnamoyltransferase
88	gi|17366672|sp|Q9ARF9.1|HPPD_PLESU	47736	20.60	2	1	1	4-hydroxyphenylpyruvate dioxygenase; AltName: Full=4-hydroxyphenylpyruvic acid oxidase; Short=4HPPD; Short=HPD; Short=HPPDase
89	gi|753709941|gb|AJI44435.1|	41648	38.44	2	1	1	Oxoglutarate-dependent flavone 7-O-demethylase [Ocimum basilicum]
90	gi|827345132|gb|AKJ76716.1|	218366	38.05	1	1	1	Protein TIC 214 [Rosmarinus officinalis]
91	gi|836643372|ref|YP_009144573.1|	218366	38.05	1	1	1	Protein TIC 214 [Rosmarinus officinalis]
92	gi|827346602|gb|AKJ77788.1|	181164	21.27	1	1	1	Protein TIC 214 [Perilla frutescens]
93	gi|745791067|gb|AJD25242.1|	53008	22.18	3	1	1	cytochrome P450 CYP707A102 [Salvia miltiorrhiza]
94	gi|745790971|gb|AJD25194.1|	55908	22.03	2	1	1	cytochrome P450 CYP81B62 [Salvia miltiorrhiza]

No.	Accession Number	Average Mass	Max-10logP	Max Coverage (%)	Max# Peptides	Max# Unique	Description
95	gi|410176144|gb|AFV61803.1|	26773	21.57	5	1	1	30S ribosomal protein S2 (chloroplast) [Origanum vulgare subsp. vulgare]
96	gi|916442902|gb|AKZ23975.1|	26773	21.57	5	1	1	ribosomal protein S2 (plastid) [Monarda fistulosa var. mollis]
97	gi|916442908|gb|AKZ23977.1|	26686	21.57	5	1	1	ribosomal protein S2 (plastid) [Nepeta cataria]
98	gi|827346574|gb|AKJ77760.1|	26722	21.57	5	1	1	30S ribosomal protein S2 (chloroplast) [Perilla frutescens]
99	gi|827345166|gb|AKJ76750.1|	26770	21.57	5	1	1	30S ribosomal protein S2 (chloroplast) [Rosmarinus officinalis]
100	gi|836643406|ref|YP_009144504.1|	26770	21.57	5	1	1	30S ribosomal protein S2 (chloroplast) [Rosmarinus officinalis]
101	gi|401879731|gb|AFQ30918.1|	26744	21.57	5	1	1	30S ribosomal protein S2 (chloroplast) [Salvia miltiorrhiza]
102	gi|573461940|emb|CCQ71609.1|	26744	21.57	5	1	1	30S ribosomal protein S2 (chloroplast) [Salvia miltiorrhiza]
103	gi|916442905|gb|AKZ23976.1|	26758	21.57	5	1	1	ribosomal protein S2 (plastid) [Salvia nemorosa]
104	gi|442775714|gb|AGC73980.1|	19816	20.22	6	1	1	jasmonate ZIM-domain protein 1 [Salvia miltiorrhiza]
105	gi|745791091|gb|AJD25254.1|	55430	20.07	1	1	1	cytochrome P450 CYP728D17 [Salvia miltiorrhiza]
106	gi|8134569|sp|Q42662.2|METE_PLESU	84590	20.07	1	1	1	5-methyltetrahydropteroyltriglutamate--homocysteine methyltransferase; AltName: Full=Cobalamin-independent methionine synthase isozyme; AltName: Full=Vitamin-B12-independent methionine synthase isozyme
107	gi|29839421|sp|Q9XGW0.1|COMT1_OCIBA	39529	20.07	1	1	1	Caffeic acid 3-O-methyltransferase 1; Short=CAOMT-1; Short=COMT-1; AltName: Full=S-adenosysl-L-methionine:caffeic acid 3-O-methyltransferase 1

No.	Accession Number	Average Mass	Max-10logP	Max Coverage (%)	Max# Peptides	Max# Unique	Description
108	gi|29839420|sp|Q9XGV9.1|COMT2_OCIBA	39613	20.07	1	1	1	Caffeic acid 3-O-methyltransferase 2; Short=CAOMT-2; Short=COMT-2; AltName: Full=S-adenosysl-L-methionine:caffeic acid 3-O-methyltransferase 2
109	gi|735679295|gb|AJA39985.1|	82378	20.07	1	1	1	(E)-4-hydroxy-3-methylbut-2-enyl diphosphate synthase [Salvia miltiorrhiza f. alba]
110	gi|75219538|sp|O48935.1|TPSBF_MENPI	63830	109.4	23	14	13	Beta-farnesene synthase
111	gi|122249145|sp|Q49SP3.1|TPSPS_POGCB	64199	102.97	22	14	13	Patchoulol synthase; Short=PatTps177; AltName: Full=Alpha-guaiene synthase; AltName: Full=Delta-guaiene synthase
112	gi|116256299|sp|Q9XES0.2|DXR_MENPI	51034	80.68	11	7	7	1-deoxy-D-xylulose 5-phosphate reductoisomerase, chloroplastic; Short=1-deoxyxylulose-5-phosphate reductoisomerase; Short=DXP reductoisomerase; AltName: Full=2-C-methyl-D-erythritol 4-phosphate synthase; F...
113	gi|75192856|sp|Q9MBC1.1|3AT_PERFR	50675	78.82	8	4	4	Anthocyanidin 3-O-glucoside 6''-O-acyltransferase; Short=3AT
114	gi|29839420|sp|Q9XGV9.1|COMT2_OCIBA	39613	77.93	6	3	1	Caffeic acid 3-O-methyltransferase 2; Short=CAOMT-2; Short=COMT-2; AltName: Full=S-adenosysl-L-methionine:caffeic acid 3-O-methyltransferase 2
115	gi|75293243|sp|Q6WKZ0.1|C7D94_MENGR	56308	76.56	7	4	4	Cytochrome P450 71D94
116	gi|403399409|sp|E2E2P0.1|GTPS_ORIVU	69063	74.1	5	4	4	Gamma-terpinene synthase, chloroplastic; Short=Ovtps2; AltName: Full=Alpha-terpinene synthase; Flags: Precursor
117	gi|29839421|sp|Q9XGW0.1|COMT1_OCIBA	39529	72.15	6	3	1	Caffeic acid 3-O-methyltransferase 1; Short=CAOMT-1; Short=COMT-1; AltName: Full=S-adenosysl-L-methionine:caffeic acid 3-O-methyltransferase 1
118	gi|132044|sp|P28453.1|RBL_SCUBO	51794	68.35	12	5	5	Ribulose bisphosphate carboxylase large chain; Short=RuBisCO large subunit

No.	Accession Number	Average Mass	Max-10logP	Max Coverage (%)	Max# Peptides	Max# Unique	Description
119	gi|8134569|sp|Q42662.2|METE_PLESU	84590	56.89	4	3	3	5-methyltetrahydropteroyltriglutamate--homocysteine methyltransferase; AltName: Full=Cobalamin-independent methionine synthase isozyme; AltName: Full=Vitamin-B12-independent methionine synthase isozyme
120	gi|75129654|sp|Q6VMW0.1|Q8OMT_MENPI	40849	43.32	6	2	2	8-hydroxyquercetin 8-O-methyltransferase; AltName: Full=Flavonol 8-O-methyltransferase
121	gi|75293242|sp|Q6WKY9.1|C7D95_MENGR	56365	42.08	2	1	1	Cytochrome P450 71D95; AltName: Full=(-)-(4S)-Limonene-3-hydroxylase
122	gi|75290511|sp|Q6IV13.1|C7D95_MENSP	56322	42.08	2	1	1	Cytochrome P450 71D95; AltName: Full=Limonene-3-hydroxylase
123	gi|75306222|sp|Q947B7.1|MFS_MENPI	55360	30.62	2	1	1	(+)-menthofuran synthase; AltName: Full=(+)-pulegone 9-hydroxylase
124	gi|17366672|sp|Q9ARF9.1|HPPD_PLESU	47736	28.03	2	1	1	4-hydroxyphenylpyruvate dioxygenase; AltName: Full=4-hydroxyphenylpyruvic acid oxidase; Short=4HPPD; Short=HPD; Short=HPPDase
125	gi|510785777|sp|G0LD36.1|RAS_MELOI	47161	26.9	2	1	1	Rosmarinate synthase; Short=MoRAS; AltName: Full=Hydroxycinnamoyl-CoA:hydroxyphenyllactate hydroxycinnamoyltransferase
126	gi|75129878|sp|Q6WAU0.1|PULR_MENPI	37915	26.37	2	1	1	(+)-pulegone reductase
127	gi|122237148|sp|Q15GI4.1|EGS1_OCIBA	35607	22.15	3	1	1	Eugenol synthase 1 [Ocimum basilicum] (Sweet basil)
128	gi|56749087|sp|Q85XY6.1|MATK_OCIBA	60282	20.19	2	1	1	Maturase K; AltName: Full=Intron maturase
129	gi|29839421|sp|Q9XGW0.1|COMT1_OCIBA	39529	28.48	1	1	1	Caffeic acid 3-O-methyltransferase 1; Short=CAOMT-1; Short=COMT-1; AltName: Full=S-adenosysl-L-methionine:caffeic acid 3-O-methyltransferase 1
130	gi|8134569|sp|Q42662.2|METE_PLESU	84590	37.07	1	1	1	5-methyltetrahydropteroyltriglutamate--homocysteine methyltransferase; AltName: Full=Cobalamin-independent methionine synthase isozyme; AltName:

No.	Accession Number	Average Mass	Max-10logP	Max Coverage (%)	Max# Peptides	Max# Unique	Description
							Full=Vitamin-B12-independent methionine synthase isozyme
131	gi|29839420|sp|Q9XGV9.1|COMT2_OCIBA	39613	28.48	1	1	1	Caffeic acid 3-O-methyltransferase 2; Short=CAOMT-2; Short=COMT-2; AltName: Full=S-adenosysl-L-methionine:caffeic acid 3-O-methyltransferase 2
132	gi|735679295|gb|AJA39985.1|	82378	28.48	1	1	1	(E)-4-hydroxy-3-methylbut-2-enyl diphosphate synthase [Salvia miltiorrhiza f. alba]
133	gi|745791091|gb|AJD25254.1|	55430	28.48	1	1	1	cytochrome P450 CYP728D17 [Salvia miltiorrhiza]
134	gi|916438881|gb|AKZ22145.1|	84354	39.87	1	1	1	NADH dehydrogenase subunit 5 (plastid) [Monarda fistulosa var. mollis]
135	gi|410176201|gb|AFV61860.1|	84295	39.87	1	1	1	NADH dehydrogenase subunit 5 (chloroplast) [Origanum vulgare subsp. vulgare]
136	gi|669254287|gb|AII20583.1|	77022	34.71	2	1	1	NADH dehydrogenase subunit F, partial (chloroplast) [Holocheila longipedunculata]
137	gi|669254291|gb|AII20585.1|	79070	34.71	2	1	1	NADH dehydrogenase subunit F, partial (chloroplast) [Petraeovitex multiflora]
138	gi|669254285|gb|AII20582.1|	74815	34.71	2	1	1	NADH dehydrogenase subunit F, partial (chloroplast) [Holocheila longipedunculata]
139	gi|669254283|gb|AII20581.1|	77991	34.71	2	1	1	NADH dehydrogenase subunit F, partial (chloroplast) [Holocheila longipedunculata]
140	gi|395484522|gb|AFN66518.1|	76105	34.71	2	1	1	NADH dehydrogenase subunit F, partial (plastid) [Teucrium subspinosum]
141	gi|669254293|gb|AII20586.1|	79081	34.71	2	1	1	NADH dehydrogenase subunit F, partial (chloroplast) [Hymenopyramis cana]
142	gi|401879790|gb|AFQ30977.1|	83587	34.70	1	1	1	NADH dehydrogenase subunit 5 (chloroplast) [Salvia miltiorrhiza]

No.	Accession Number	Average Mass	Max-10logP	Max Coverage (%)	Max# Peptides	Max# Unique	Description
143	gi|573462000|emb|CCQ71669.1|	83587	34.70	1	1	1	NADH dehydrogenase subunit 5 (chloroplast) [Salvia miltiorrhiza]
144	gi|395484502|gb|AFN66509.1|	75956	34.71	2	1	1	NADH dehydrogenase subunit F, partial (plastid) [Teucrium divaricatum]
145	gi|395484481|gb|AFN66500.1|	76061	34.71	2	1	1	NADH dehydrogenase subunit F, partial (plastid) [Teucrium flavum subsp. glaucum]
146	gi|395484475|gb|AFN66497.1|	78510	34.71	2	1	1	NADH dehydrogenase subunit F, partial (plastid) [Rubiteucris palmata]
147	gi|836643377|ref|YP_009144562.1|	84218	34.71	1	1	1	NADH dehydrogenase subunit 5 (chloroplast) [Rosmarinus officinalis]
148	gi|827345137|gb|AKJ76721.1|	84218	34.71	1	1	1	NADH dehydrogenase subunit 5 (chloroplast) [Rosmarinus officinalis]
149	gi|916438885|gb|AKZ22147.1|	84443	34.71	1	1	1	NADH dehydrogenase subunit 5 (plastid) [Salvia nemorosa]
150	gi|395484513|gb|AFN66514.1|	76814	26.97	2	1	1	NADH dehydrogenase subunit F, partial (plastid) [Teucrium pyrenaicum]
151	gi|827346594|gb|AKJ77780.1|	84248	34.71	1	1	1	NADH dehydrogenase subunit 5 (chloroplast) [Perilla frutescens]
152	gi|827345833|gb|AKJ77134.1|	85675	26.97	1	1	1	NADH dehydrogenase subunit 5 (chloroplast) [Scutellaria baicalensis]
153	gi|910312648|ref|YP_009162309.1|	85675	26.97	1	1	1	NADH dehydrogenase subunit 5 (chloroplast) [Scutellaria baicalensis]
154	gi|395484511|gb|AFN66513.1|	76860	26.97	2	1	1	NADH dehydrogenase subunit F, partial (plastid) [Teucrium oxylepis]
155	gi|752789835|ref|YP_009117269.1|	85385	34.71	1	1	1	NADH-plastoquinone oxidoreductase subunit 5 (chloroplast) [Premna microphylla]

No.	Accession Number	Average Mass	Max-10logP	Max Coverage (%)	Max# Peptides	Max# Unique	Description
156	gi|748013953|gb|AJE28423.1|	85385	34.71	1	1	1	NADH-plastoquinone oxidoreductase subunit 5 (chloroplast) [Premna microphylla]
157	gi|395484489|gb|AFN66504.1|	76910	26.97	2	1	1	NADH dehydrogenase subunit F, partial (plastid) [Teucrium betonicum]
158	gi|916438887|gb|AKZ22148.1|	83254	26.97	1	1	1	NADH dehydrogenase subunit 5 (plastid) [Teucrium canadense]
159	gi|395484495|gb|AFN66506.1|	76993	26.97	2	1	1	NADH dehydrogenase subunit F, partial (plastid) [Teucrium eriocephalum subsp. almeriense]
160	gi|395484500|gb|AFN66508.1|	77065	26.97	2	1	1	NADH dehydrogenase subunit F, partial (plastid) [Teucrium stocksianum subsp. incanum]
161	gi|395484526|gb|AFN66520.1|	76596	26.97	2	1	1	NADH dehydrogenase subunit F, partial (plastid) [Teucrium bicolor]
162	gi|395484515|gb|AFN66515.1|	76501	26.97	2	1	1	NADH dehydrogenase subunit F, partial (plastid) [Teucrium racemosum]
163	gi|395484473|gb|AFN66496.1|	78531	26.97	2	1	1	NADH dehydrogenase subunit F, partial (plastid) [Teucridium parvifolium]
164	gi|395484524|gb|AFN66519.1|	75723	26.97	2	1	1	NADH dehydrogenase subunit F, partial (plastid) [Teucrium kotschyanum]
165	gi|395484477|gb|AFN66498.1|	76293	26.97	2	1	1	NADH dehydrogenase subunit F, partial (plastid) [Spartothamnella puberula]
166	gi|395484517|gb|AFN66516.1|	76688	26.97	2	1	1	NADH dehydrogenase subunit F, partial (plastid) [Teucrium salviastrum]
167	gi|395484520|gb|AFN66517.1|	76892	26.97	2	1	1	NADH dehydrogenase subunit F, partial (plastid) [Teucrium spinosum]
168	gi|395484483|gb|AFN66501.1|	77431	26.97	2	1	1	NADH dehydrogenase subunit F, partial (plastid) [Teucrium albicaule]

No.	Accession Number	Average Mass	Max-10logP	Max Coverage (%)	Max# Peptides	Max# Unique	Description
169	gi|395484504|gb|AFN66510.1|	77454	26.97	2	1	1	NADH dehydrogenase subunit F, partial (plastid) [Teucrium laciniatum]
170	gi|395484530|gb|AFN66522.1|	76725	26.97	2	1	1	NADH dehydrogenase subunit F, partial (plastid) [Teucrium viscidum var. miquelianum]
171	gi|395484528|gb|AFN66521.1|	76705	26.97	2	1	1	NADH dehydrogenase subunit F, partial (plastid) [Teucrium japonicum]
172	gi|395484537|gb|AFN66525.1|	78210	26.97	2	1	1	NADH dehydrogenase subunit F, partial (plastid) [Teucrium decipiens]
173	gi|395484533|gb|AFN66523.1|	75763	26.97	2	1	1	NADH dehydrogenase subunit F, partial (plastid) [Teucrium montbretii subsp. heliotropiifolium]
174	gi|395484507|gb|AFN66511.1|	77434	26.97	2	1	1	NADH dehydrogenase subunit F, partial (plastid) [Teucrium nudicaule]
175	gi|395484479|gb|AFN66499.1|	34714	26.97	4	1	1	NADH dehydrogenase subunit F, partial (plastid) [Oncinocalyx betchei]
176	gi|395484535|gb|AFN66524.1|	76413	26.97	2	1	1	NADH dehydrogenase subunit F, partial (plastid) [Teucrium antitauricum]
177	gi|395484493|gb|AFN66505.1|	77203	26.97	2	1	1	NADH dehydrogenase subunit F, partial (plastid) [Teucrium pseudochamaepitys]
178	gi|395484487|gb|AFN66503.1|	77172	26.97	2	1	1	NADH dehydrogenase subunit F, partial (plastid) [Teucrium aroanium]
179	gi|395484509|gb|AFN66512.1|	77251	26.97	2	1	1	NADH dehydrogenase subunit F, partial (plastid) [Teucrium orientale subsp. gloeotrichum]
180	gi|395484485|gb|AFN66502.1|	77595	26.97	2	1	1	NADH dehydrogenase subunit F, partial (plastid) [Teucrium aristatum]
181	gi|395484497|gb|AFN66507.1|	77873	26.97	2	1	1	NADH dehydrogenase subunit F, partial (plastid) [Teucrium fruticans]

No.	Accession Number	Average Mass	Max-10logP	Max Coverage (%)	Max# Peptides	Max# Unique	Description
182	gi|827345131|gb|AKJ76715.1|	266537	38.65	0	1	1	Protein Ycf2 [Rosmarinus officinalis]
183	gi|827345130|gb|AKJ76714.1|	266537	38.65	0	1	1	Protein Ycf2 [Rosmarinus officinalis]
184	gi|836643371|ref|YP_009144577.1|	266537	38.65	0	1	1	Protein Ycf2 [Rosmarinus officinalis]
185	gi|836643370|ref|YP_009144558.1|	266537	38.65	0	1	1	Protein Ycf2 [Rosmarinus officinalis]
186	gi|573462016|emb|CCQ71685.1|	267080	38.65	0	1	1	Protein Ycf2 [Salvia miltiorrhiza]
187	gi|573461995|emb|CCQ71664.1|	267080	38.65	0	1	1	Protein Ycf2 [Salvia miltiorrhiza]
188	gi|401879785|gb|AFQ30972.1|	267080	38.65	0	1	1	Protein Ycf2 [Salvia miltiorrhiza]
189	gi|401879806|gb|AFQ30993.1|	267080	38.65	0	1	1	Protein Ycf2 [Salvia miltiorrhiza]
190	gi|748013968|gb|AJE28438.1|	268103	38.65	0	1	1	Protein Ycf2 [Premna microphylla]
191	gi|752789850|ref|YP_009117284.1|	268103	38.65	0	1	1	Protein Ycf2 [Premna microphylla]
192	gi|752789831|ref|YP_009117265.1|	268103	38.65	0	1	1	Protein Ycf2 [Premna microphylla]
193	gi|748013949|gb|AJE28419.1|	268103	38.65	0	1	1	Protein Ycf2 [Premna microphylla]
194	gi|910312663|ref|YP_009162324.1|	268069	38.65	0	1	1	Protein Ycf2 [Scutellaria baicalensis]
195	gi|827345827|gb|AKJ77128.1|	268069	38.65	0	1	1	Protein Ycf2 [Scutellaria baicalensis]
196	gi|910312644|ref|YP_009162305.1|	268069	38.65	0	1	1	Protein Ycf2 [Scutellaria baicalensis]
197	gi|827345826|gb|AKJ77127.1|	268069	38.65	0	1	1	Protein Ycf2 [Scutellaria baicalensis]
198	gi|827346597|gb|AKJ77783.1|	266476	38.65	0	1	1	Protein Ycf2 [Perilla frutescens]
199	gi|827346579|gb|AKJ77765.1|	266476	38.65	0	1	1	Protein Ycf2 [Perilla frutescens]
200	gi|827346602|gb|AKJ77788.1|	181164	30.65	1	1	1	Protein TIC 214 [Perilla frutescens]
201	gi|827345132|gb|AKJ76716.1|	218366	21.51	1	1	1	Protein TIC 214 [Rosmarinus officinalis]

No.	Accession Number	Average Mass	Max-10logP	Max Coverage (%)	Max# Peptides	Max# Unique	Description
202	gi|836643372|ref|YP_009144573.1|	218366	21.51	1	1	1	Protein TIC 214 [Rosmarinus officinalis]
203	gi|748013964|gb|AJE28434.1|	225117	29.24	1	1	1	Protein TIC 214 [Premna microphylla]
204	gi|752789846|ref|YP_009117280.1|	225117	29.24	1	1	1	Protein TIC 214 [Premna microphylla]
205	gi|75192856|sp|Q9MBC1.1|3AT_PERFR	50675	28.93	2	1	1	Anthocyanidin 3-O-glucoside 6''-O-acyltransferase; Short=3AT
206	gi|749489569|emb|CEO43479.1|	50534	28.93	2	1	1	unnamed protein product [Lavandula angustifolia] - patented sequence
207	gi|745791067|gb|AJD25242.1|	53008	26.07	3	1	1	cytochrome P450 CYP707A102 [Salvia miltiorrhiza]
208	gi|410176144|gb|AFV61803.1|	26773	25.72	5	1	1	ribosomal protein S2 (chloroplast) [Origanum vulgare subsp. vulgare]
209	gi|916442902|gb|AKZ23975.1|	26773	25.72	5	1	1	ribosomal protein S2 (plastid) [Monarda fistulosa var. mollis]
210	gi|916442908|gb|AKZ23977.1|	26686	25.72	5	1	1	ribosomal protein S2 (plastid) [Nepeta cataria]
211	gi|827346574|gb|AKJ77760.1|	26722	25.72	5	1	1	ribosomal protein S2 (chloroplast) [Perilla frutescens]
212	gi|827345166|gb|AKJ76750.1|	26770	25.72	5	1	1	ribosomal protein S2 (chloroplast) [Rosmarinus officinalis]
213	gi|836643406|ref|YP_009144504.1|	26770	25.72	5	1	1	ribosomal protein S2 (chloroplast) [Rosmarinus officinalis]
214	gi|401879731|gb|AFQ30918.1|	26744	25.72	5	1	1	ribosomal protein S2 (chloroplast) [Salvia miltiorrhiza]
215	gi|573461940|emb|CCQ71609.1|	26744	25.72	5	1	1	ribosomal protein S2 (chloroplast) [Salvia miltiorrhiza]
216	gi|916442905|gb|AKZ23976.1|	26758	25.72	5	1	1	ribosomal protein S2 (plastid) [Salvia nemorosa]
217	gi|787592954|gb|AKA27904.1|	30582	23.75	4	1	1	WRKY protein [Salvia miltiorrhiza]

No.	Accession Number	Average Mass	Max-10logP	Max Coverage (%)	Max# Peptides	Max# Unique	Description
218	gi|659902912|gb|AID69536.1|	58940	22.81	2	1	1	phenylalanine ammonia-lyase, partial [Phlomoides rotata]
219	gi|735665579|gb|AJA38250.1|	90993	20.04	2	1	1	copalyl diphosphate synthase, partial [Salvia fruticosa]
220	gi|763711578|gb|AJQ30184.1|	90993	20.04	2	1	1	copalyl diphosphate synthase, partial [Salvia fruticosa]
221	gi|122233627|sp|Q4JF75.1|RBR_SCUBA	111795	107.9	14	16	16	Retinoblastoma-related protein
222	gi|62900766|sp|O81193.1|SSS_SALOF	68942	92.38	9	5	4	(+)-sabinene synthase, chloroplastic; Short=SSS; Flags: Precursor
223	gi|29839420|sp|Q9XGV9.1|COMT2_OCIBA	39613	89.09	15	7	4	Caffeic acid 3-O-methyltransferase 2; Short=CAOMT-2; Short=COMT-2; AltName: Full=S-adenosysl-L-methionine:caffeic acid 3-O-methyltransferase 2
224	gi|122249145|sp|Q49SP3.1|TPSPS_POGCB	64199	88.62	15	8	7	Patchoulol synthase; Short=PatTps177; AltName: Full=Alpha-guaiene synthase; AltName: Full=Delta-guaiene synthase
225	gi|62900763|sp|O81191.1|SCS_SALOF	69369	84.53	10	6	6	1,8-cineole synthase, chloroplastic; Short=SCS; Flags: Precursor
226	gi|510785778|sp|A0PDV5.1|RAS_PLESU	47902	81.71	8	5	5	Rosmarinate synthase; Short=CbRAS; AltName: Full=Hydroxycinnamoyl transferase; Short=CbHCT1
227	gi|29839421|sp|Q9XGW0.1|COMT1_OCIBA	39529	78.79	11	5	2	Caffeic acid 3-O-methyltransferase 1; Short=CAOMT-1; Short=COMT-1; AltName: Full=S-adenosysl-L-methionine:caffeic acid 3-O-methyltransferase 1
228	gi|75251477|sp|Q5SBP0.1|TPSD_OCIBA	70000	72.11	9	5	4	Terpinolene synthase, chloroplastic; Flags: Precursor
229	gi|8134569|sp|Q42662.2|METE_PLESU	84590	71.27	6	5	5	5-methyltetrahydropteroyltriglutamate--homocysteine methyltransferase; AltName: Full=Cobalamin-independent methionine synthase isozyme; AltName: Full=Vitamin-B12-independent methionine synthase isozyme

No.	Accession Number	Average Mass	Max-10logP	Max Coverage (%)	Max# Peptides	Max# Unique	Description
230	gi|75251484|sp|Q5SBP7.1|SELS_OCIBA	63125	71.11	7	4	4	Selinene synthase
231	gi|122219294|sp|Q49SP6.1|TPGD2_POGCB	64149	67.75	4	2	1	Germacrene D synthase 2; AltName: Full=PatTpsBF2
232	gi|62899675|sp|O81192.1|BPPS_SALOF	69292	65.17	3	2	1	(+)-bornyl diphosphate synthase, chloroplastic; Short=BPPS; AltName: Full=(+)-alpha-pinene synthase; AltName: Full=(+)-camphene synthase; AltName: Full=SBS; Flags: Precursor
233	gi|122210943|sp|Q2XSC5.1|LALIN_LAVAN	65654	56.70	2	1	1	R-linalool synthase; Short=LaLINS
234	gi|75251482|sp|Q5SBP5.1|GCS1_OCIBA	63566	48.42	1	1	1	Gamma-cadinene synthase; AltName: Full=(+)-gamma-cadinene synthase
235	gi|75129878|sp|Q6WAU0.1|PULR_MENPI	37915	46.22	2	1	1	(+)-pulegone reductase
236	gi|75315261|sp|Q9XHE8.1|C71DI_MENSP	56149	37.31	4	2	1	Cytochrome P450 71D18; AltName: Full=(-)-(4S)-Limonene-6-hydroxylase
237	gi|75293244|sp|Q6WKZ1.1|C71DI_MENGR	56149	37.31	4	2	1	Cytochrome P450 71D18; AltName: Full=(-)-(4S)-Limonene-6-hydroxylase
238	gi|75180331|sp|Q9LRC8.1|BAGLU_SCUBA	58772	33.32	6	2	2	Baicalin-beta-D-glucuronidase; AltName: Full=Baicalinase; Flags: Precursor
239	gi|75315259|sp|Q9XHE6.1|C71DF_MENPI	56532	32.17	4	2	2	Cytochrome P450 71D15; AltName: Full=(-)-(4S)-Limonene-3-hydroxylase; AltName: Full=Cytochrome P450 isoform PM2
240	gi|75161989|sp|Q8W1W9.1|5MAT1_SALSN	50724	31.33	2	1	1	Malonyl-coenzyme:anthocyanin 5-O-glucoside-6'''-O-malonyltransferase; Short=Malonyl CoA:anthocyanin 5-O-glucoside-6'''-O-malonyltransferase; Short=Ss5MaT1
241	gi|75293243|sp|Q6WKZ0.1|C7D94_MENGR	56308	31.28	4	2	1	Cytochrome P450 71D94
242	gi|704000326|sp|S4UX02.1|CYPH1_SALMI	55520	31.06	1	1	1	Ferruginol synthase; AltName: Full=Cytochrome P450 76AH1

No.	Accession Number	Average Mass	Max-10logP	Max Coverage (%)	Max# Peptides	Max# Unique	Description
243	gi|75338882|sp|Q9ZR27.1|5GT1_PERFR	50974	31.00	2	1	1	Anthocyanidin 3-O-glucoside 5-O-glucosyltransferase 1; AltName: Full=UDP-glucose:anthocyanin 5-O-glucosyltransferase 3R4; Short=p3R4; Flags: Precursor
244	gi|75338881|sp|Q9ZR26.1|5GT2_PERFR	49110	22.61	2	1	1	Anthocyanidin 3-O-glucoside 5-O-glucosyltransferase 2; AltName: Full=UDP-glucose:anthocyanin 5-O-glucosyltransferase 3R6; Short=p3R6; Flags: Precursor
245	gi|122200954|sp|Q2KNL5.1|CADH1_OCIBA	38769	23.05	4	1	1	Cinnamyl alcohol dehydrogenase 1; Short=CAD 1; Short=ObaCAD1
246	gi|75227033|sp|Q76MR7.1|UBGAT_SCUBA	48654	22.49	5	1	1	Baicalein 7-O-glucuronosyltransferase [Scutellaria baicalensis](Baical skullcap)
247	gi|84029472|sp|Q93WU3.1|CVMT1_OCIBA	39916	20.44	3	1	1	Chavicol O-methyltransferase [Ocimum basilicum] (Sweet basil)<br>Status
248	gi|84027871|sp|Q93WU2.1|EOMT1_OCIBA	40237	20.44	3	1	1	Eugenol O-methyltransferase; AltName: Full=(Iso)eugenol O-methyltransferase EOMT1; AltName: Full=S-adenosysl-L-methionine:(Iso)eugenol O-methyltransferase EOMT1
249	gi|401879801|gb|AFQ30988.1|	218699	41.13	1	2	2	Photosystem I assembly protein Ycf1 (chloroplast) [Salvia miltiorrhiza]
250	gi|573462011|emb|CCQ71680.1|	218685	41.13	1	2	2	Photosystem I assembly protein Ycf1 (chloroplast) [Salvia miltiorrhiza]
251	gi|836643389|ref|YP_009144556.1|	29997	28.27	3	1	1	ribosomal protein L2 (chloroplast) [Rosmarinus officinalis]
252	gi|827345149|gb|AKJ76733.1|	29997	28.27	3	1	1	ribosomal protein L2 (chloroplast) [Rosmarinus officinalis]
253	gi|827345150|gb|AKJ76734.1|	29997	28.27	3	1	1	ribosomal protein L2 (chloroplast) [Rosmarinus officinalis]

No.	Accession Number	Average Mass	Max-10logP	Max Coverage (%)	Max# Peptides	Max# Unique	Description
254	gi|836643390|ref|YP_009144579.1|	29997	28.27	3	1	1	ribosomal protein L2 (chloroplast) [Rosmarinus officinalis]
255	gi|401879808|gb|AFQ30995.1|	29997	28.27	3	1	1	ribosomal protein L2 (chloroplast) [Salvia miltiorrhiza]
256	gi|401879783|gb|AFQ30970.1|	29997	28.27	3	1	1	ribosomal protein L2 (chloroplast) [Salvia miltiorrhiza]
257	gi|916441525|gb|AKZ23467.1|	29921	28.27	3	1	1	ribosomal protein L2 (plastid) [Nepeta cataria]
258	gi|410176218|gb|AFV61877.1|	29997	28.27	3	1	1	ribosomal protein L2 (chloroplast) [Origanum vulgare subsp. vulgare]
259	gi|410176195|gb|AFV61854.1|	29997	28.27	3	1	1	ribosomal protein L2 (chloroplast) [Origanum vulgare subsp. vulgare]
260	gi|916441521|gb|AKZ23465.1|	29997	28.27	3	1	1	ribosomal protein L2 (plastid) [Monarda fistulosa var. mollis]
261	gi|916441523|gb|AKZ23466.1|	29997	28.27	3	1	1	ribosomal protein L2 (plastid) [Salvia nemorosa]
262	gi|752789852|ref|YP_009117286.1|	30084	28.27	3	1	1	ribosomal protein L2 (chloroplast) [Premna microphylla]
263	gi|752789829|ref|YP_009117263.1|	30084	28.27	3	1	1	ribosomal protein L2 (chloroplast) [Premna microphylla]
264	gi|827346587|gb|AKJ77773.1|	31403	28.27	3	1	1	ribosomal protein L2 (chloroplast) [Perilla frutescens]
265	gi|827346537|gb|AKJ77723.1|	31391	28.27	3	1	1	ribosomal protein L2 (chloroplast) [Perilla frutescens]
266	gi|910312642|ref|YP_009162303.1|	30114	28.27	3	1	1	ribosomal protein L2 (chloroplast) [Scutellaria baicalensis]
267	gi|910312665|ref|YP_009162326.1|	30114	28.27	3	1	1	ribosomal protein L2 (chloroplast) [Scutellaria baicalensis]
268	gi|827345846|gb|AKJ77147.1|	30114	28.27	3	1	1	ribosomal protein L2 (chloroplast) [Scutellaria baicalensis]

No.	Accession Number	Average Mass	Max-10logP	Max Coverage (%)	Max# Peptides	Max# Unique	Description
269	gi|827345845|gb|AKJ77146.1|	30114	28.27	3	1	1	ribosomal protein L2 (chloroplast) [Scutellaria baicalensis]
270	gi|916441533|gb|AKZ23471.1|	30010	28.27	3	1	1	ribosomal protein L2 (plastid) [Teucrium canadense]
271	gi|836643406|ref|YP_009144504.1|	26770	27.53	5	1	1	ribosomal protein S2 (chloroplast) [Rosmarinus officinalis]
272	gi|827345166|gb|AKJ76750.1|	26770	27.53	5	1	1	ribosomal protein S2 (chloroplast) [Rosmarinus officinalis]
273	gi|401879731|gb|AFQ30918.1|	26744	27.53	5	1	1	ribosomal protein S2 (chloroplast) [Salvia miltiorrhiza]
274	gi|573461940|emb|CCQ71609.1|	26744	27.53	5	1	1	ribosomal protein S2 (chloroplast) [Salvia miltiorrhiza]
275	gi|827346574|gb|AKJ77760.1|	26722	27.53	5	1	1	ribosomal protein S2 (chloroplast) [Perilla frutescens]
276	gi|410176144|gb|AFV61803.1|	26773	27.53	5	1	1	ribosomal protein S2 (chloroplast) [Origanum vulgare subsp. vulgare]
277	gi|916442905|gb|AKZ23976.1|	26758	27.53	5	1	1	ribosomal protein S2 (plastid) [Salvia nemorosa]
278	gi|916442902|gb|AKZ23975.1|	26773	27.53	5	1	1	ribosomal protein S2 (plastid) [Monarda fistulosa var. mollis]
279	gi|916442908|gb|AKZ23977.1|	26686	27.53	5	1	1	ribosomal protein S2 (plastid) [Nepeta cataria]
280	gi|916441821|gb|AKZ23615.1|	17837	25.53	7	1	1	ribosomal protein L22 (plastid) [Salvia nemorosa]
281	gi|913341377|gb|AKU77131.1|	121334	23.61	1	1	1	Structural maintenance of chromosomes protein 1, partial [Callicarpa bodinieri]
282	gi|735679295|gb|AJA39985.1|	82378	22.90	1	1	1	(E)-4-hydroxy-3-methylbut-2-enyl diphosphate synthase [Salvia miltiorrhiza f. alba]
283	gi|762060299|gb|AJQ20621.1|	41314	21.58	2	1	1	Enoyl-ACP Reductase [Salvia miltiorrhiza]
284	gi|661525312|gb|AIE15763.1|	216665	20.56	1	1	1	Dicer-like protein 1 [Salvia miltiorrhiza]

No.	Accession Number	Average Mass	Max-10logP	Max Coverage (%)	Max# Peptides	Max# Unique	Description
285	gi|908373664|gb|AKT44364.1|	81096	20.41	1	1	1	Heat shock protein 2 [Tectona grandis]
286	gi|8134569|sp|Q42662.2|METE_PLESU	84590	115.32	8	7	7	5-methyltetrahydropteroyltriglutamate--homocysteine methyltransferase; AltName: Full=Cobalamin-independent methionine synthase isozyme; AltName: Full=Vitamin-B12-independent methionine synthase isozyme
287	gi|5915814|sp|O04164.1|C71A6_NEPRA	57955	92.61	5	3	3	Cytochrome P450 71A6
288	gi|29839421|sp|Q9XGW0.1|COMT1_OCIBA	39529	90.64	6	3	1	Caffeic acid 3-O-methyltransferase 1; Short=CAOMT-1; Short=COMT-1; AltName: Full=S-adenosysl-L-methionine:caffeic acid 3-O-methyltransferase 1
289	gi|5915815|sp|Q42716.1|C71A8_MENPI	57213	90.02	5	3	3	Cytochrome P450 71A8
290	gi|122219292|sp|Q49SP4.1|TPGD1_POGCB	64197	86.02	3	2	2	Germacrene D synthase 1; AltName: Full=PatTpsB15
291	gi|5921781|sp|O04111.1|CHSY_PERFR	42686	85.72	5	2	2	Chalcone synthase; AltName: Full=Naringenin-chalcone synthase
292	gi|122233627|sp|Q4JF75.1|RBR_SCUBA	111795	73.59	3	2	2	Retinoblastoma-related protein
293	gi|84027871|sp|Q93WU2.1|EOMT1_OCIBA	40237	59.55	3	1	1	Eugenol O-methyltransferase; AltName: Full=(Iso)eugenol O-methyltransferase EOMT1; AltName: Full=S-adenosysl-L-methionine:(Iso)eugenol O-methyltransferase EOMT1
294	gi|122219294|sp|Q49SP6.1|TPGD2_POGCB	64149	59.19	3	1	1	Germacrene D synthase 2; AltName: Full=PatTpsBF2
295	gi|75161989|sp|Q8W1W9.1|5MAT1_SALSN	50724	46.86	2	1	1	Malonyl-coenzyme:anthocyanin 5-O-glucoside-6'''-O-malonyltransferase; Short=Malonyl CoA:anthocyanin 5-O-glucoside-6'''-O-malonyltransferase; Short=Ss5MaT1
296	gi|75251484|sp|Q5SBP7.1|SELS_OCIBA	63125	43.61	2	1	1	Selinene synthase

No.	Accession Number	Average Mass	Max-10logP	Max Coverage (%)	Max# Peptides	Max# Unique	Description
297	gi|122249145|sp|Q49SP3.1|TPSPS_POGCB	64199	42.70	4	1	1	Patchoulol synthase; Short=PatTps177; AltName: Full=Alpha-guaiene synthase; AltName: Full=Delta-guaiene synthase
298	gi|75293243|sp|Q6WKZ0.1|C7D94_MENGR	56308	40.60	3	1	1	Cytochrome P450 71D94
299	gi|75338882|sp|Q9ZR27.1|5GT1_PERFR	50974	37.18	2	1	1	Anthocyanidin 3-O-glucoside 5-O-glucosyltransferase 1; AltName: Full=UDP-glucose:anthocyanin 5-O-glucosyltransferase 3R4; Short=p3R4; Flags: Precursor
300	gi|75180331|sp|Q9LRC8.1|BAGLU_SCUBA	58772	37.03	5	1	1	Baicalin-beta-D-glucuronidase; AltName: Full=Baicalinase; Flags: Precursor
301	gi|704000326|sp|S4UX02.1|CYPH1_SALMI	55520	35.26	1	1	1	Ferruginol synthase; AltName: Full=Cytochrome P450 76AH1
302	gi|75129878|sp|Q6WAU0.1|PULR_MENPI	37915	33.26	2	1	1	(+)-pulegone reductase
303	gi|75227033|sp|Q76MR7.1|UBGAT_SCUBA	48654	27.59	5	1	1	Baicalein 7-O-glucuronosyltransferase; AltName: Full=UDP-glucuronate:baicalein 7-O-glucuronosyltransferase
304	gi|661525316|gb|AIE15765.1|	184302	48.48	1	1	1	Dicer-like protein 3 [Salvia miltiorrhiza]
305	gi|836643406|ref|YP_009144504.1|	26770	34.08	5	1	1	Ribosomal protein S2 (chloroplast) [Rosmarinus officinalis]
306	gi|827345166|gb|AKJ76750.1|	26770	34.08	5	1	1	Ribosomal protein S2 (chloroplast) [Rosmarinus officinalis]
307	gi|401879731|gb|AFQ30918.1|	26744	34.08	5	1	1	Ribosomal protein S2 (chloroplast) [Salvia miltiorrhiza]
308	gi|573461940|emb|CCQ71609.1|	26744	34.08	5	1	1	Ribosomal protein S2 (chloroplast) [Salvia miltiorrhiza]
309	gi|410176144|gb|AFV61803.1|	26773	34.08	5	1	1	Ribosomal protein S2 (chloroplast) [Origanum vulgare subsp. vulgare]

No.	Accession Number	Average Mass	Max-10logP	Max Coverage (%)	Max# Peptides	Max# Unique	Description
310	gi|916442902|gb|AKZ23975.1|	26773	34.08	5	1	1	Ribosomal protein S2 (plastid) [Monarda fistulosa var. mollis]
311	gi|916442905|gb|AKZ23976.1|	26758	34.08	5	1	1	Ribosomal protein S2 (plastid) [Salvia nemorosa]
312	gi|916442908|gb|AKZ23977.1|	26686	34.08	5	1	1	Ribosomal protein S2 (plastid) [Nepeta cataria]
313	gi|827346574|gb|AKJ77760.1|	26722	34.08	5	1	1	Ribosomal protein S2 (chloroplast) [Perilla frutescens]
314	gi|827345132|gb|AKJ76716.1|	218366	31.19	1	1	1	Ycf1 (chloroplast) [Rosmarinus officinalis]
315	gi|836643372|ref|YP_009144573.1|	218366	31.19	1	1	1	Ycf1 (chloroplast) [Rosmarinus officinalis]
316	gi|401879801|gb|AFQ30988.1|	218699	30.62	1	1	1	Photosystem I assembly protein Ycf1 (chloroplast) [Salvia miltiorrhiza]
317	gi|573462011|emb|CCQ71680.1|	218685	30.62	1	1	1	Ycf1 (chloroplast) [Salvia miltiorrhiza]
318	gi|787592932|gb|AKA27893.1|	37310	25.11	4	1	1	WRKY protein [Salvia miltiorrhiza]
319	gi|662170387|gb|AIE45494.1|	44939	24.14	3	1	1	CONSTANS-like protein 9 [Tectona grandis]
320	gi|745790929|gb|AJD25173.1|	57680	22.85	1	1	1	Cytochrome P450 CYP73A120 [Salvia miltiorrhiza]
321	gi|630057998|gb|AHY94893.1|	57803	22.85	1	1	1	Cinnamate-4-hydroxylase [Prunella vulgaris]
322	gi|725812545|gb|AIY32618.1|	57979	22.85	1	1	1	Cinnamate-4-hydroxylase [Perilla frutescens]
323	gi|745791023|gb|AJD25220.1|	56140	22.76	2	1	1	Cytochrome P450 CYP94B50 [Salvia miltiorrhiza]
324	gi|762060309|gb|AJQ20626.1|	51963	21.88	4	1	1	Ketoacyl-ACP Synthase I [Salvia miltiorrhiza]
325	gi|844572842|gb|AKN09607.1|	76725	21.06	1	1	1	Basic helix-loop-helix transcription factor [Salvia miltiorrhiza]
326	gi|762060281|gb|AJQ20612.1|	13344	20.65	12	1	1	Acyl Carrier Protein [Salvia miltiorrhiza]
327	gi|822603217|emb|CQR79430.1|	35399	20.39	3	1	1	Maturase K, partial (chloroplast) [Clerodendrum bracteatum]

No.	Accession Number	Average Mass	Max-10logP	Max Coverage (%)	Max# Peptides	Max# Unique	Description
328	gi|630058023|gb|AHY94894.1|	51993	20.11	2	1	1	4-coumarate:CoA ligase, partial [Prunella vulgaris]
329	gi|29839421|sp|Q9XGW0.1|COMT1_OCIBA	39529	20.10	2	1	1	Caffeic acid 3-O-methyltransferase 1; Short=CAOMT-1; Short=COMT-1; AltName: Full=S-adenosysl-L-methionine:caffeic acid 3-O-methyltransferase 1
330	gi|29839420|sp|Q9XGV9.1|COMT2_OCIBA	39613	20.10	2	1	1	Caffeic acid 3-O-methyltransferase 2; Short=CAOMT-2; Short=COMT-2; AltName: Full=S-adenosysl-L-methionine:caffeic acid 3-O-methyltransferase 2
331	gi|122219293|sp|Q49SP5.1|TPGAS_POGCB	64232	129.73	17	10	10	Germacrene A synthase; AltName: Full=PatTpsCF2
332	gi|75283876|sp|Q5C9I9.1|ISPD_MENPI	27191	102.75	18	5	5	(-)-isopiperitenol/(-)-carveol dehydrogenase, mitochondrial; Flags: Precursor
333	gi|8134569|sp|Q42662.2|METE_PLESU	84590	91.09	3	2	2	5-methyltetrahydropteroyltriglutamate--homocysteine methyltransferase; AltName: Full=Cobalamin-independent methionine synthase isozyme; AltName: Full=Vitamin-B12-independent methionine synthase isozyme
334	gi|75219538|sp|O48935.1|TPSBF_MENPI	63830	90.41	4	2	2	Beta-farnesene synthase
335	gi|75252096|sp|Q5W283.1|TPSCM_MENPI	63839	85.44	4	2	2	Cis-muuroladiene synthase; Short=MxpSS1
336	gi|75129878|sp|Q6WAU0.1|PULR_MENPI	37915	89.97	6	3	3	(+)-pulegone reductase
337	gi|75224312|sp|Q6USK1.1|GERS_OCIBA	64933	84.74	2	1	1	Geraniol synthase, chloroplastic; Short=ObGES; Flags: Precursor
338	gi|75293243|sp|Q6WKZ0.1|C7D94_MENGR	56308	75.65	4	2	2	Cytochrome P450 71D94
339	gi|403399409|sp|E2E2P0.1|GTPS_ORIVU	69063	74.54	2	1	1	Gamma-terpinene synthase, chloroplastic; Short=Ovtps2; AltName: Full=Alpha-terpinene synthase; Flags: Precursor

No.	Accession Number	Average Mass	Max-10logP	Max Coverage (%)	Max# Peptides	Max# Unique	Description
340	gi|75161989|sp|Q8W1W9.1|5MAT1_SALSN	50724	49.68	2	1	1	Malonyl-coenzyme:anthocyanin 5-O-glucoside-6'''-O-malonyltransferase; Short=Malonyl CoA:anthocyanin 5-O-glucoside-6'''-O-malonyltransferase; Short=Ss5MaT1
341	gi|122210943|sp|Q2XSC5.1|LALIN_LAVAN	65654	48.77	2	1	1	R-linalool synthase; Short=LaLINS
342	gi|75251481|sp|Q5SBP4.1|AZIS_OCIBA	62858	25.63	2	1	1	Alpha-zingiberene synthase
343	gi|748013964|gb|AJE28434.1|	225117	38.72	1	2	1	hypothetical chloroplast RF19 (chloroplast) [Premna microphylla]
344	gi|752789846|ref|YP_009117280.1|	225117	38.72	1	2	1	hypothetical chloroplast RF19 (chloroplast) [Premna microphylla]
345	gi|916443350|gb|AKZ24105.1|	17446	33.46	5	1	1	ribosomal protein S7 (plastid) [Teucrium canadense]
346	gi|827345872|gb|AKJ77173.1|	17389	33.46	5	1	1	ribosomal protein S7 (chloroplast) [Scutellaria baicalensis]
347	gi|910312647|ref|YP_009162308.1|	17389	33.46	5	1	1	ribosomal protein S7 (chloroplast) [Scutellaria baicalensis]
348	gi|827345873|gb|AKJ77174.1|	17389	33.46	5	1	1	ribosomal protein S7 (chloroplast) [Scutellaria baicalensis]
349	gi|827345177|gb|AKJ76761.1|	17379	33.46	5	1	1	ribosomal protein S7 (chloroplast) [Rosmarinus officinalis]
350	gi|827345176|gb|AKJ76760.1|	17379	33.46	5	1	1	ribosomal protein S7 (chloroplast) [Rosmarinus officinalis]
351	gi|836643417|ref|YP_009144574.1|	17379	33.46	5	1	1	ribosomal protein S7 (chloroplast) [Rosmarinus officinalis]
352	gi|836643416|ref|YP_009144561.1|	17379	33.46	5	1	1	ribosomal protein S7 (chloroplast) [Rosmarinus officinalis]

No.	Accession Number	Average Mass	Max-10logP	Max Coverage (%)	Max# Peptides	Max# Unique	Description
353	gi|916443332|gb|AKZ24099.1|	17391	33.46	5	1	1	ribosomal protein S7 (plastid) [Monarda fistulosa var. mollis]
354	gi|573462013|emb|CCQ71682.1|	17361	33.46	5	1	1	ribosomal protein S7 (chloroplast) [Salvia miltiorrhiza]
355	gi|401879803|gb|AFQ30990.1|	17361	33.46	5	1	1	ribosomal protein S7 (chloroplast) [Salvia miltiorrhiza]
356	gi|573461998|emb|CCQ71667.1|	17361	33.46	5	1	1	ribosomal protein S7 (chloroplast) [Salvia miltiorrhiza]
357	gi|401879788|gb|AFQ30975.1|	17361	33.46	5	1	1	ribosomal protein S7 (chloroplast) [Salvia miltiorrhiza]
358	gi|410176200|gb|AFV61859.1|	17361	33.46	5	1	1	ribosomal protein S7 (chloroplast) [Origanum vulgare subsp. vulgare]
359	gi|410176213|gb|AFV61872.1|	17361	33.46	5	1	1	ribosomal protein S7 (chloroplast) [Origanum vulgare subsp. vulgare]
360	gi|752789834|ref|YP_009117268.1|	17361	33.46	5	1	1	ribosomal protein S7 (chloroplast) [Premna microphylla]
361	gi|748013965|gb|AJE28435.1|,	17361	33.46	5	1	1	ribosomal protein S7 (chloroplast) [Premna microphylla]
362	gi|752789847|ref|YP_009117281.1|	17361	33.46	5	1	1	ribosomal protein S7 (chloroplast) [Premna microphylla]
363	gi|748013952|gb|AJE28422.1|	17361	33.46	5	1	1	ribosomal protein S7 (chloroplast) [Premna microphylla]
364	gi|827346599|gb|AKJ77785.1|	17361	33.46	5	1	1	ribosomal protein S7 (chloroplast) [Perilla frutescens]
365	gi|827346590|gb|AKJ77776.1|	17361	33.46	5	1	1	ribosomal protein S7 (chloroplast) [Perilla frutescens]
366	gi|916443335|gb|AKZ24100.1|	17379	33.46	5	1	1	ribosomal protein S7 (plastid) [Salvia nemorosa]
367	gi|910312660|ref|YP_009162321.1|	17389	33.46	5	1	1	ribosomal protein S7 (chloroplast) [Scutellaria baicalensis]
368	gi|916443338|gb|AKZ24101.1|	17361	33.46	5	1	1	ribosomal protein S7 (plastid) [Nepeta cataria]
369	gi|661525316|gb|AIE15765.1|	184302	22.76	1	1	1	Dicer-like protein 3 [Salvia miltiorrhiza]

No.	Accession Number	Average Mass	Max-10logP	Max Coverage (%)	Max# Peptides	Max# Unique	Description
370	gi|75290511|sp|Q6IV13.1|C7D95_MENSP	56322	168.40	9	6	1	Cytochrome P450 71D95; AltName: Full=Limonene-3-hydroxylase
371	gi|122233627|sp|Q4JF75.1|RBR_SCUBA	111795	136.20	1	1	1	Retinoblastoma-related protein
372	gi|75129878|sp|Q6WAU0.1|PULR_MENPI	37915	114.35	6	3	3	(+)-pulegone reductase
373	gi|122249145|sp|Q49SP3.1|TPSPS_POGCB	64199	106.65	3	2	2	Patchoulol synthase; Short=PatTps177; AltName: Full=Alpha-guaiene synthase; AltName: Full=Delta-guaiene synthase
374	gi|403399735|sp|E2E2N7.1|BCGS_ORIVU	64443	54.15	2	1	1	Bicyclogermacrene synthase; Short=Ovtps4
375	gi|661525316|gb|AIE15765.1|	184302	47.70	1	1	1	Dicer-like protein 3 [Salvia miltiorrhiza]
376	gi|910312663|ref|YP_009162324.1|	268069	41.48	0	1	1	Hypothetical chloroplast RF2 (chloroplast) [Scutellaria baicalensis]
377	gi|827345827|gb|AKJ77128.1|	268069	41.48	0	1	1	Hypothetical chloroplast RF2 (chloroplast) [Scutellaria baicalensis]
378	gi|910312644|ref|YP_009162305.1|	268069	41.48	0	1	1	Hypothetical chloroplast RF2 (chloroplast) [Scutellaria baicalensis]
379	gi|827345826|gb|AKJ77127.1|	268069	41.48	0	1	1	Hypothetical chloroplast RF2 (chloroplast) [Scutellaria baicalensis]
380	gi|748013968|gb|AJE28438.1|	268103	36.17	0	1	1	Hypothetical chloroplast RF21 (chloroplast) [Premna microphylla]
381	gi|752789850|ref|YP_009117284.1|	268103	36.17	0	1	1	Hypothetical chloroplast RF21 (chloroplast) [Premna microphylla]
382	gi|752789831|ref|YP_009117265.1|	268103	36.17	0	1	1	Hypothetical chloroplast RF21 (chloroplast) [Premna microphylla]
383	gi|748013949|gb|AJE28419.1|	268103	36.17	0	1	1	Hypothetical chloroplast RF21 (chloroplast) [Premna microphylla]

No.	Accession Number	Average Mass	Max-10logP	Max Coverage (%)	Max# Peptides	Max# Unique	Description
384	gi|827346597|gb|AKJ77783.1|	266476	36.17	0	1	1	Ycf2 (chloroplast) [Perilla frutescens]
385	gi|827346579|gb|AKJ77765.1|	266476	36.17	0	1	1	Ycf2 (chloroplast) [Perilla frutescens]
386	gi|836643370|ref|YP_009144558.1|	266537	36.17	0	1	1	Ycf2 (chloroplast) [Rosmarinus officinalis]
387	gi|827345131|gb|AKJ76715.1|	266537	36.17	0	1	1	Ycf2 (chloroplast) [Rosmarinus officinalis]
388	gi|827345130|gb|AKJ76714.1|	266537	36.17	0	1	1	Ycf2 (chloroplast) [Rosmarinus officinalis]
389	gi|836643371|ref|YP_009144577.1|	266537	36.17	0	1	1	Ycf2 (chloroplast) [Rosmarinus officinalis]
390	gi|573462016|emb|CCQ71685.1|	267080	36.17	0	1	1	Ycf2 (chloroplast) [Salvia miltiorrhiza]
391	gi|573461995|emb|CCQ71664.1|	267080	36.17	0	1	1	Ycf2 (chloroplast) [Salvia miltiorrhiza]
392	gi|401879785|gb|AFQ30972.1|	267080	36.17	0	1	1	Hypothetical chloroplast RF2 (chloroplast) [Salvia miltiorrhiza]
393	gi|401879806|gb|AFQ30993.1|	267080	36.17	0	1	1	Hypothetical chloroplast RF2 (chloroplast) [Salvia miltiorrhiza]
394	gi|410176216|gb|AFV61875.1|	264781	36.17	0	1	1	Ycf2 (chloroplast) [Origanum vulgare subsp. vulgare]
395	gi|410176197|gb|AFV61856.1|	264781	36.17	0	1	1	Ycf2 (chloroplast) [Origanum vulgare subsp. vulgare]
396	gi|661525312|gb|AIE15763.1|	216665	28.26	1	1	1	Dicer-like protein 1 [Salvia miltiorrhiza]
397	gi|585636485|gb|AHJ59322.1|	90900	24.77	1	1	1	Copalyl diphosphate synthase [Salvia miltiorrhiza f. alba]
398	gi|751414476|gb|AJF93403.1|	58880	24.19	2	1	1	Ent-kaurene oxidase [Salvia miltiorrhiza]
399	gi|745791049|gb|AJD25233.1|	58838	24.19	2	1	1	Cytochrome P450 CYP701A40 [Salvia miltiorrhiza]
400	gi|410176144|gb|AFV61803.1|	26773	23.20	5	1	1	ribosomal protein S2 (chloroplast) [Origanum vulgare subsp. vulgare]
401	gi|916442902|gb|AKZ23975.1|	26773	23.20	5	1	1	ribosomal protein S2 (plastid) [Monarda fistulosa var. mollis]

No.	Accession Number	Average Mass	Max-10logP	Max Coverage (%)	Max# Peptides	Max# Unique	Description
402	gi|916442908|gb|AKZ23977.1|	26686	23.20	5	1	1	ribosomal protein S2 (plastid) [Nepeta cataria]
403	gi|827346574|gb|AKJ77760.1|	26722	23.20	5	1	1	ribosomal protein S2 (chloroplast) [Perilla frutescens]
404	gi|836643406|ref|YP_009144504.1|	26770	23.20	5	1	1	ribosomal protein S2 (chloroplast) [Rosmarinus officinalis]
405	gi|827345166|gb|AKJ76750.1|	26770	23.20	5	1	1	ribosomal protein S2 (chloroplast) [Rosmarinus officinalis]
406	gi|401879731|gb|AFQ30918.1|	26744	23.20	5	1	1	ribosomal protein S2 (chloroplast) [Salvia miltiorrhiza]
407	gi|573461940|emb|CCQ71609.1|	26744	23.20	5	1	1	ribosomal protein S2 (chloroplast) [Salvia miltiorrhiza]
408	gi|916442905|gb|AKZ23976.1|	26758	23.20	5	1	1	ribosomal protein S2 (plastid) [Salvia nemorosa]
409	gi|75129878|sp|Q6WAU0.1|PULR_MENPI	37915	21.98	2	1	1	(+)-pulegone reductase
410	gi|787592958|gb|AKA27906.1|	38850	20.03	3	1	1	WRKY protein [Salvia miltiorrhiza]
411	gi|122219294|sp|Q49SP6.1|TPGD2_POGCB	64149	107.08	8	5	5	Germacrene D synthase 2; AltName: Full=PatTpsBF2
412	gi|122233627|sp|Q4JF75.1|RBR_SCUBA	111795	101.83	4	4	4	Retinoblastoma-related protein
413	gi|56749087|sp|Q85XY6.1|MATK_OCIBA	60282	86.78	4	2	2	Maturase K; AltName: Full=Intron maturase
414	gi|75219538|sp|O48935.1|TPSBF_MENPI	63830	84.66	5	3	3	Beta-farnesene synthase
415	gi|75252096|sp|Q5W283.1|TPSCM_MENPI	63839	81.24	5	3	3	Cis-muuroladiene synthase; Short=MxpSS1
416	gi|122219293|sp|Q49SP5.1|TPGAS_POGCB	64232	80.48	1	1	1	Germacrene A synthase; AltName: Full=PatTpsCF2
417	gi|510785777|sp|G0LD36.1|RAS_MELOI	47161	71.10	7	3	3	Rosmarinate synthase; Short=MoRAS; AltName: Full=Hydroxycinnamoyl-CoA:hydroxyphenyllactate hydroxycinnamoyltransferase
418	gi|75251480|sp|Q5SBP3.1|LLOS_OCIBA	65822	67.77	3	2	2	R-linalool synthase, chloroplastic; Flags: Precursor

No.	Accession Number	Average Mass	Max-10logP	Max Coverage (%)	Max# Peptides	Max# Unique	Description
419	gi|6919914|sp|P56848.1|ISPE_MENPI	44603	63.19	5	2	2	4-diphosphocytidyl-2-C-methyl-D-erythritol kinase, chloroplastic; AltName: Full=4-(cytidine-5'-diphospho)-2-C-methyl-D-erythritol kinase; Short=CMK; Flags: Precursor
420	gi|75251484|sp|Q5SBP7.1|SELS_OCIBA	63125	53.85	2	1	1	Selinene synthase
421	gi|122219295|sp|Q49SP7.1|TPSCS_POGCB	63586	46.67	2	1	1	Gamma-curcumene synthase; AltName: Full=PatTpsA
422	gi|75129878|sp|Q6WAU0.1|PULR_MENPI	37915	29.75	2	1	1	(+)-pulegone reductase
423	gi|75293243|sp|Q6WKZ0.1|C7D94_MENGR	56308	26.13	2	1	1	Cytochrome P450 71D94
424	gi|75251480|sp|Q5SBP3.1|LLOS_OCIBA	65822	44.02	5	3	3	R-linalool synthase, chloroplastic; Flags: Precursor
425	gi|844572842|gb|AKN09607.1|	76725	22.88	1	1	1	Basic helix-loop-helix transcription factor [Salvia miltiorrhiza]
426	gi|916443332|gb|AKZ24099.1|	17391	22.37	5	1	1	ribosomal protein S7 (plastid) [Monarda fistulosa var. mollis]
427	gi|916443350|gb|AKZ24105.1|	17446	22.37	5	1	1	ribosomal protein S7 (plastid) [Teucrium canadense]
428	gi|827345872|gb|AKJ77173.1|	17389	22.37	5	1	1	ribosomal protein S7 (chloroplast) [Scutellaria baicalensis]
429	gi|910312647|ref|YP_009162308.1|	17389	22.37	5	1	1	ribosomal protein S7 (chloroplast) [Scutellaria baicalensis]
430	gi|827345177|gb|AKJ76761.1|	17379	22.37	5	1	1	ribosomal protein S7 (chloroplast) [Rosmarinus officinalis]
431	gi|827345176|gb|AKJ76760.1|	17379	22.37	5	1	1	ribosomal protein S7 (chloroplast) [Rosmarinus officinalis]
432	gi|836643417|ref|YP_009144574.1|	17379	22.37	5	1	1	ribosomal protein S7 (chloroplast) [Rosmarinus officinalis]

No.	Accession Number	Average Mass	Max-10logP	Max Coverage (%)	Max# Peptides	Max# Unique	Description
433	gi|836643416|ref|YP_009144561.1|	17379	22.37	5	1	1	ribosomal protein S7 (chloroplast) [Rosmarinus officinalis]
434	gi|573462013|emb|CCQ71682.1|	17361	22.37	5	1	1	ribosomal protein S7 (chloroplast) [Salvia miltiorrhiza]
435	gi|401879803|gb|AFQ30990.1|	17361	22.37	5	1	1	ribosomal protein S7 (chloroplast) [Salvia miltiorrhiza]
436	gi|573461998|emb|CCQ71667.1|	17361	22.37	5	1	1	ribosomal protein S7 (chloroplast) [Salvia miltiorrhiza]
437	gi|401879788|gb|AFQ30975.1|	17361	22.37	5	1	1	ribosomal protein S7 (chloroplast) [Salvia miltiorrhiza]
438	gi|410176200|gb|AFV61859.1|	17361	22.37	5	1	1	ribosomal protein S7 (chloroplast) [Origanum vulgare subsp. vulgare]
439	gi|752789834|ref|YP_009117268.1|	17361	22.37	5	1	1	ribosomal protein S7 (chloroplast) [Premna microphylla]
440	gi|748013965|gb|AJE28435.1|	17361	22.37	5	1	1	ribosomal protein S7 (chloroplast) [Premna microphylla]
441	gi|752789847|ref|YP_009117281.1|	17361	22.37	5	1	1	ribosomal protein S7 (chloroplast) [Premna microphylla]
442	gi|748013952|gb|AJE28422.1|	17361	22.37	5	1	1	ribosomal protein S7 (chloroplast) [Premna microphylla]
443	gi|827345873|gb|AKJ77174.1|	17389	22.37	5	1	1	ribosomal protein S7 (chloroplast) [Scutellaria baicalensis]
444	gi|910312660|ref|YP_009162321.1|	17389	22.37	5	1	1	ribosomal protein S7 (chloroplast) [Scutellaria baicalensis]
445	gi|827346599|gb|AKJ77785.1|	17361	22.37	5	1	1	ribosomal protein S7 (chloroplast) [Perilla frutescens]
446	gi|827346590|gb|AKJ77776.1|	17361	22.37	5	1	1	ribosomal protein S7 (chloroplast) [Perilla frutescens]
447	gi|410176213|gb|AFV61872.1|	17361	22.37	5	1	1	ribosomal protein S7 (chloroplast) [Origanum vulgare subsp. vulgare]
448	gi|916443335|gb|AKZ24100.1|	17379	22.37	5	1	1	ribosomal protein S7 (plastid) [Salvia nemorosa]
449	gi|916443338|gb|AKZ24101.1|	17361	22.37	5	1	1	ribosomal protein S7 (plastid) [Nepeta cataria]

No.	Accession Number	Average Mass	Max-10logP	Max Coverage (%)	Max# Peptides	Max# Unique	Description
450	gi|916439925|gb|AKZ22667.1|	82427	21.45	1	1	1	photosystem I P700 chlorophyll a apoprotein A2 (plastid) [Nepeta cataria]
451	gi|410176154|gb|AFV61813.1|	82441	21.45	1	1	1	photosystem I P700 chlorophyll a apoprotein A2 (chloroplast) [Origanum vulgare subsp. vulgare]
452	gi|916439921|gb|AKZ22665.1|	82459	21.45	1	1	1	photosystem I P700 chlorophyll a apoprotein A2 (plastid) [Monarda fistulosa var. mollis]
453	gi|827345836|gb|AKJ77137.1|	82407	21.45	1	1	1	photosystem I P700 apoprotein A2 (chloroplast) [Scutellaria baicalensis]
454	gi|910312599|ref|YP_009162260.1|	82407	21.45	1	1	1	photosystem I P700 apoprotein A2 (chloroplast) [Scutellaria baicalensis]
455	gi|836643380|ref|YP_009144514.1|	82458	21.45	1	1	1	photosystem I P700 chlorophyll a apoprotein A2 (chloroplast) [Rosmarinus officinalis]
456	gi|827345140|gb|AKJ76724.1|	82458	21.45	1	1	1	photosystem I P700 chlorophyll a apoprotein A2 (chloroplast) [Rosmarinus officinalis]
457	gi|916439923|gb|AKZ22666.1|	82440	21.45	1	1	1	photosystem I P700 chlorophyll a apoprotein A2 (plastid) [Salvia nemorosa]
458	gi|752789785|ref|YP_009117220.1|	82392	21.45	1	1	1	photosystem I P700 apoprotein A2 (chloroplast) [Premna microphylla]
459	gi|573461950|emb|CCQ71619.1|	82459	21.45	1	1	1	photosystem I P700 chlorophyll a apoprotein A2 (chloroplast) [Salvia miltiorrhiza]
460	gi|916439933|gb|AKZ22671.1|	82411	21.45	1	1	1	photosystem I P700 chlorophyll a apoprotein A2 (plastid) [Teucrium canadense]
461	gi|401879741|gb|AFQ30928.1|	82459	21.45	1	1	1	photosystem I P700 apoprotein A2 (chloroplast) [Salvia miltiorrhiza]
462	gi|748013903|gb|AJE28373.1|	82392	21.45	1	1	1	photosystem I P700 apoprotein A2 (chloroplast) [Premna microphylla]

No.	Accession Number	Average Mass	Max-10logP	Max Coverage (%)	Max# Peptides	Max# Unique	Description
463	gi|827346564|gb|AKJ77750.1|	82379	21.45	1	1	1	photosystem I P700 chlorophyll a apoprotein A2 (chloroplast) [Perilla frutescens]
464	gi|122219294|sp|Q49SP6.1|TPGD2_POGCB	64149	163.8	30	23	22	Germacrene D synthase 2; AltName: Full=PatTpsBF2
465	gi|403399409|sp|E2E2P0.1|GTPS_ORIVU	69063	113.8	11	10	10	Gamma-terpinene synthase, chloroplastic; Short=Ovtps2; AltName: Full=Alpha-terpinene synthase; Flags: Precursor
466	gi|75315260|sp|Q9XHE7.1|C71DD_MENPI	56601	111.98	10	5	5	Cytochrome P450 71D13; AltName: Full=(-)-(4S)-Limonene-3-hydroxylase; AltName: Full=Cytochrome P450 isoform PM17
467	gi|29839420|sp|Q9XGV9.1|COMT2_OCIBA	39613	108.84	23	9	6	Caffeic acid 3-O-methyltransferase 2; Short=CAOMT-2; Short=COMT-2; AltName: Full=S-adenosysl-L-methionine:caffeic acid 3-O-methyltransferase 2
468	gi|29839421|sp|Q9XGW0.1|COMT1_OCIBA	39529	100.08	15	5	2	Caffeic acid 3-O-methyltransferase 1; Short=CAOMT-1; Short=COMT-1; AltName: Full=S-adenosysl-L-methionine:caffeic acid 3-O-methyltransferase 1
469	gi|8134569|sp|Q42662.2|METE_PLESU	84590	92.48	5	4	4	5-methyltetrahydropteroyltriglutamate--homocysteine methyltransferase; AltName: Full=Cobalamin-independent methionine synthase isozyme; AltName: Full=Vitamin-B12-independent methionine synthase isozyme
470	gi|75180331|sp|Q9LRC8.1|BAGLU_SCUBA	58772	74.70	6	3	3	Baicalin-beta-D-glucuronidase; AltName: Full=Baicalinase; Flags: Precursor
471	gi|75244696|sp|Q8H2B4.1|LLOS_MENAQ	70535	72.02	1	1	1	R-linalool synthase, chloroplastic; Flags: Precursor
472	gi|75192856|sp|Q9MBC1.1|3AT_PERFR	50675	71.34	5	2	2	Anthocyanidin 3-O-glucoside 6''-O-acyltransferase; Short=3AT
473	gi|122219295|sp|Q49SP7.1|TPSCS_POGCB	63586	49.89	2	1	1	Gamma-curcumene synthase; AltName: Full=PatTpsA
474	gi|75251484|sp|Q5SBP7.1|SELS_OCIBA	63125	47.56	2	1	1	Selinene synthase

No.	Accession Number	Average Mass	Max-10logP	Max Coverage (%)	Max# Peptides	Max# Unique	Description
475	gi|75293243|sp|Q6WKZ0.1|C7D94_MENGR	56308	40.29	2	1	1	Cytochrome P450 71D94
476	gi|75306222|sp|Q947B7.1|MFS_MENPI	55360	38.64	2	1	1	(+)-menthofuran synthase; AltName: Full=(+)-pulegone 9-hydroxylase
477	gi|75129878|sp|Q6WAU0.1|PULR_MENPI	37915	28.57	2	1	1	(+)-pulegone reductase
478	gi|75227033|sp|Q76MR7.1|UBGAT_SCUBA	48654	25.41	2	1	1	Baicalein 7-O-glucuronosyltransferase; AltName: Full=UDP-glucuronate:baicalein 7-O-glucuronosyltransferase
479	gi|395484483|gb|AFN66501.1|	77431	41.88	3	2	1	NADH dehydrogenase subunit F, partial (plastid) [Teucrium albicaule]
480	gi|401879790|gb|AFQ30977.1|	83587	37.73	3	2	1	NADH dehydrogenase subunit 5 (chloroplast) [Salvia miltiorrhiza]
481	gi|573462000|emb|CCQ71669.1|	83587	37.73	3	2	1	NADH dehydrogenase subunit 5 (chloroplast) [Salvia miltiorrhiza]
482	gi|395484473|gb|AFN66496.1|	78531	41.39	3	2	1	NADH dehydrogenase subunit F, partial (plastid) [Teucridium parvifolium]
483	gi|762060711|gb|AJQ20633.1|	73689	38.32	2	1	1	Long-Chain Acyl-CoA Synthetase [Salvia miltiorrhiza]
484	gi|913341331|gb|AKU77108.1|	104619	30.23	1	1	1	Structural maintenance of chromosomes protein 2, partial [Callicarpa bodinieri]
485	gi|827346602|gb|AKJ77788.1|	181164	29.32	1	1	1	Ycf1, partial (chloroplast) [Perilla frutescens]
486	gi|844572722|gb|AKN09568.1|	41656	28.76	2	1	1	Basic helix-loop-helix transcription factor [Salvia miltiorrhiza]
487	gi|691200620|gb|AIR77798.1|	26940	25.37	4	1	1	TCP transcription factor, partial [Perovskia atriplicifolia]
488	gi|691200582|gb|AIR77779.1|	29756	25.37	4	1	1	TCP transcription factor, partial [Tectona grandis]
489	gi|691200714|gb|AIR77845.1|	29953	25.37	4	1	1	TCP transcription factor, partial [Gmelina arborea]

No.	Accession Number	Average Mass	Max-10logP	Max Coverage (%)	Max# Peptides	Max# Unique	Description
490	gi|691200580|gb|AIR77778.1|	30438	25.37	4	1	1	TCP transcription factor, partial [Congea tomentosa]
491	gi|691200590|gb|AIR77783.1|	26615	32.97	4	1	1	TCP transcription factor, partial [Origanum vulgare]
492	gi|691200584|gb|AIR77780.1|	30608	25.37	4	1	1	TCP transcription factor, partial [Callicarpa cathayana]
493	gi|691200708|gb|AIR77842.1|	29615	25.37	4	1	1	TCP transcription factor, partial [Congea tomentosa]
494	gi|691200712|gb|AIR77844.1|	29757	25.37	4	1	1	TCP transcription factor, partial [Holmskioldia sanguinea]
495	gi|691200594|gb|AIR77785.1|	25444	25.37	4	1	1	TCP transcription factor, partial [Mentha longifolia]
496	gi|691200596|gb|AIR77786.1|	25755	25.37	4	1	1	TCP transcription factor, partial [Mentha longifolia]
497	gi|691200624|gb|AIR77800.1|	29841	25.37	4	1	1	TCP transcription factor, partial [Mentha longifolia]
498	gi|691200622|gb|AIR77799.1|	29930	25.37	4	1	1	TCP transcription factor, partial [Premna fulva]
499	gi|691200630|gb|AIR77803.1|	26302	25.37	4	1	1	TCP transcription factor, partial [Ocimum basilicum]
500	gi|75251484|sp|Q5SBP7.1|SELS_OCIBA	63125	24.81	2	1	1	Selinene synthase
501	gi|510794468|gb|AGN52200.1|	23112	24.18	3	1	1	MYB-related transcription factor [Salvia miltiorrhiza]
502	gi|662858700|gb|AIE77092.1|	90197	20.42	1	1	1	(+)-copalyl diphosphate synthase [Marrubium vulgare]
503	gi|837370038|gb|AKM94187.1|	20521	20.13	6	1	1	Maturase K, partial (chloroplast) [Leonotis nepetifolia]
504	gi|837370329|gb|AKM94283.1|	28841	20.13	4	1	1	Maturase K, partial (chloroplast) [Leonotis nepetifolia]
505	gi|837372516|gb|AKM95003.1|	28942	20.13	4	1	1	Maturase K, partial (chloroplast) [Leonotis nepetifolia]
506	gi|837371203|gb|AKM94570.1|	20329	20.13	6	1	1	Maturase K, partial (chloroplast) [Leonotis nepetifolia]
507	gi|122233627|sp|Q4JF75.1|RBR_SCUBA	111795	128.39	19	22	22	Retinoblastoma-related protein
508	gi|75244696|sp|Q8H2B4.1|LLOS_MENAQ	70535	102.21	17	14	13	R-linalool synthase, chloroplastic; Flags: Precursor

No.	Accession Number	Average Mass	Max-10logP	Max Coverage (%)	Max# Peptides	Max# Unique	Description
509	gi|5915815|sp|Q42716.1|C71A8_MENPI	57213	98.88	23	12	12	Cytochrome P450 71A8
510	gi|75251478|sp|Q5SBP1.1|MYRS_OCIBA	69964	98.67	16	12	8	Beta-myrcene synthase, chloroplastic; Flags: Precursor
511	gi|75251477|sp|Q5SBP0.1|TPSD_OCIBA	70000	75.90	7	6	2	Terpinolene synthase, chloroplastic; Flags: Precursor
512	gi|75251480|sp|Q5SBP3.1|LLOS_OCIBA	65822	66.64	6	3	3	R-linalool synthase, chloroplastic; Flags: Precursor
513	gi|62899675|sp|O81192.1|BPPS_SALOF	69292	55.98	4	3	3	(+)-bornyl diphosphate synthase, chloroplastic; Short=BPPS; AltName: Full=(+)-alpha-pinene synthase; AltName: Full=(+)-camphene synthase; AltName: Full=SBS; Flags: Precursor
514	gi|403399409|sp|E2E2P0.1|GTPS_ORIVU	69063	49.59	3	2	2	Gamma-terpinene synthase, chloroplastic; Short=Ovtps2; AltName: Full=Alpha-terpinene synthase; Flags: Precursor
515	gi|116256299|sp|Q9XES0.2|DXR_MENPI	51034	48.60	4	2	2	1-deoxy-D-xylulose 5-phosphate reductoisomerase, chloroplastic; Short=1-deoxyxylulose-5-phosphate reductoisomerase; Short=DXP reductoisomerase; AltName: Full=2-C-methyl-D-erythritol 4-phosphate synthase; F...
516	gi|75129878|sp|Q6WAU0.1|PULR_MENPI	37915	47.66	2	1	1	(+)-pulegone reductase
517	gi|122219294|sp|Q49SP6.1|TPGD2_POGCB	64149	45.43	2	1	1	Germacrene D synthase 2; AltName: Full=PatTpsBF2
518	gi|3914571|sp|Q33600.1|RBL_LAVLA	52404	43.88	4	2	2	Ribulose bisphosphate carboxylase large chain; Short=RuBisCO large subunit; Flags: Precursor
519	gi|3914545|sp|Q31655.1|RBL_AJUCH	52455	43.88	4	2	2	Ribulose bisphosphate carboxylase large chain; Short=RuBisCO large subunit; Flags: Precursor
520	gi|132044|sp|P28453.1|RBL_SCUBO	51794	43.88	4	2	2	Ribulose bisphosphate carboxylase large chain; Short=RuBisCO large subunit
521	gi|1352807|sp|P36485.2|RBL_SALDI	52005	38.06	4	2	2	Ribulose bisphosphate carboxylase large chain; Short=RuBisCO large subunit

No.	Accession Number	Average Mass	Max-10logP	Max Coverage (%)	Max# Peptides	Max# Unique	Description
522	gi|75288825|sp|Q65CJ7.2|HPPR_PLESU	34128	42.76	3	1	1	Hydroxyphenylpyruvate reductase; Short=HPPR
523	gi|122200955|sp|Q2KNL6.1|GEDH1_OCIBA	39044	41.75	4	1	1	Geraniol dehydrogenase 1; Short=ObaGEDH1
524	gi|122219293|sp|Q49SP5.1|TPGAS_POGCB	64232	41.24	3	2	2	Germacrene A synthase; AltName: Full=PatTpsCF2
525	gi|5915814|sp|O04164.1|C71A6_NEPRA	57955	40.18	3	1	1	Cytochrome P450 71A6
526	gi|122210943|sp|Q2XSC5.1|LALIN_LAVAN	65654	36.66	2	1	1	R-linalool synthase; Short=LaLINS
527	gi|403399735|sp|E2E2N7.1|BCGS_ORIVU	64443	34.90	1	1	1	Bicyclogermacrene synthase; Short=Ovtps4
528	gi|56749087|sp|Q85XY6.1|MATK_OCIBA	60282	34.86	2	1	1	Maturase K; AltName: Full=Intron maturase
529	gi|122219295|sp|Q49SP7.1|TPSCS_POGCB	63586	32.33	3	2	2	Gamma-curcumene synthase; AltName: Full=PatTpsA
530	gi|122210942|sp|Q2XSC4.1|LABER_LAVAN	62405	29.22	2	1	1	Exo-alpha-bergamotene synthase; Short=LaBERS; AltName: Full=Trans-alpha-bergamotene synthase
531	gi|75227033|sp|Q76MR7.1|UBGAT_SCUBA	48654	28.98	2	1	1	Baicalein 7-O-glucuronosyltransferase; AltName: Full=UDP-glucuronate:baicalein 7-O-glucuronosyltransferase
532	gi|75251484|sp|Q5SBP7.1|SELS_OCIBA	63125	23.05	2	1	1	Selinene synthase
533	gi|84029472|sp|Q93WU3.1|CVMT1_OCIBA	39916	22.03	3	1	1	Chavicol O-methyltransferase; AltName: Full=(Iso)eugenol O-methyltransferase CVOMT1; AltName: Full=S-adenosysl-L-methionine:(Iso)eugenol O-methyltransferase CVOMT1
534	gi|75338881|sp|Q9ZR26.1|5GT2_PERFR	49110	21.61	2	1	1	Anthocyanidin 3-O-glucoside 5-O-glucosyltransferase 2; AltName: Full=UDP-glucose:anthocyanin 5-O-glucosyltransferase 3R6; Short=p3R6; Flags: Precursor
535	gi|75306222|sp|Q947B7.1|MFS_MENPI	55360	20.53	1	1	1	(+)-menthofuran synthase; AltName: Full=(+)-pulegone 9-hydroxylase

No.	Accession Number	Average Mass	Max-10logP	Max Coverage (%)	Max# Peptides	Max# Unique	Description
536	gi|752789776|ref|YP_009117211.1|	158193	48.06	1	1	1	RNA polymerase beta' subunit (chloroplast) [Premna microphylla]
537	gi|748013894|gb|AJE28364.1|	158193	48.06	1	1	1	RNA polymerase beta' subunit (chloroplast) [Premna microphylla]
538	gi|827346527|gb|AKJ77713.1|	158467	47.83	1	1	1	RNA polymerase beta'' subunit (chloroplast) [Perilla frutescens]
539	gi|910312590|ref|YP_009162251.1|	158800	47.83	1	1	1	RNA polymerase beta subunit-2 (chloroplast) [Scutellaria baicalensis]
540	gi|827345829|gb|AKJ77130.1|	158800	47.83	1	1	1	RNA polymerase beta subunit-2 (chloroplast) [Scutellaria baicalensis]
541	gi|916442767|gb|AKZ23937.1|	155842	47.83	1	1	1	RNA polymerase beta'' subunit (plastid) [Teucrium canadense]
542	gi|844572722|gb|AKN09568.1|	41656	30.64	2	1	1	Basic helix-loop-helix transcription factor [Salvia miltiorrhiza]
543	gi|844572778|gb|AKN09586.1|	68167	22.47	1	1	1	Basic helix-loop-helix transcription factor [Salvia miltiorrhiza]
544	gi|844572715|gb|AKN09566.1|	67854	22.47	1	1	1	Basic helix-loop-helix transcription factor [Salvia miltiorrhiza]
545	gi|748013960|gb|AJE28430.1|	19533	20.98	6	1	1	NADH-plastoquinone oxidoreductase subunit I (chloroplast) [Premna microphylla]
546	gi|752789842|ref|YP_009117276.1|	19533	20.98	6	1	1	NADH-plastoquinone oxidoreductase subunit I (chloroplast) [Premna microphylla]
547	gi|75251484|sp|Q5SBP7.1|SELS_OCIBA	63125	20.86	2	1	1	Selinene synthase
548	gi|766946303|gb|AJT36912.1|	32806	20.06	3	1	1	Ycf1, partial (chloroplast) [Vitex negundo var. negundo]
549	gi|122233627|sp|Q4JF75.1|RBR_SCUBA	111795	120.77	8	9	9	Retinoblastoma-related protein

No.	Accession Number	Average Mass	Max-10logP	Max Coverage (%)	Max# Peptides	Max# Unique	Description
550	gi|122219294|sp|Q49SP6.1|TPGD2_POGCB	64149	100.26	6	4	1	Germacrene D synthase 2; AltName: Full=PatTpsBF2
551	gi|122219292|sp|Q49SP4.1|TPGD1_POGCB	64197	73.10	6	4	1	Germacrene D synthase 1; AltName: Full=PatTpsB15
552	gi|75251482|sp|Q5SBP5.1|GCS1_OCIBA	63566	72.41	3	2	1	Gamma-cadinene synthase; AltName: Full=(+)-gamma-cadinene synthase
553	gi|62899675|sp|O81192.1|BPPS_SALOF	69292	70.24	3	2	1	(+)-bornyl diphosphate synthase, chloroplastic; Short=BPPS; AltName: Full=(+)-alpha-pinene synthase; AltName: Full=(+)-camphene synthase; AltName: Full=SBS; Flags: Precursor
554	gi|75180331|sp|Q9LRC8.1|BAGLU_SCUBA	58772	62.83	2	1	1	Baicalin-beta-D-glucuronidase; AltName: Full=Baicalinase; Flags: Precursor
555	gi|75251484|sp|Q5SBP7.1|SELS_OCIBA	63125	61.96	5	3	2	Selinene synthase
556	gi|122219293|sp|Q49SP5.1|TPGAS_POGCB	64232	51.02	3	2	2	Germacrene A synthase; AltName: Full=PatTpsCF2
557	gi|704000326|sp|S4UX02.1|CYPH1_SALMI	55520	48.40	3	1	1	Ferruginol synthase; AltName: Full=Cytochrome P450 76AH1
558	gi|75293242|sp|Q6WKY9.1|C7D95_MENGR	56365	42.87	2	1	1	Cytochrome P450 71D95; AltName: Full=(-)-(4S)-Limonene-3-hydroxylase
559	gi|75290511|sp|Q6IV13.1|C7D95_MENSP	56322	42.58	2	1	1	Cytochrome P450 71D95; AltName: Full=Limonene-3-hydroxylase
560	gi|75315260|sp|Q9XHE7.1|C71DD_MENPI	56601	42.87	2	1	1	Cytochrome P450 71D13; AltName: Full=(-)-(4S)-Limonene-3-hydroxylase; AltName: Full=Cytochrome P450 isoform PM17
561	gi|56749087|sp|Q85XY6.1|MATK_OCIBA	60282	35.91	2	1	1	Maturase K; AltName: Full=Intron maturase
562	gi|75129878|sp|Q6WAU0.1|PULR_MENPI	37915	30.97	2	1	1	(+)-pulegone reductase

No.	Accession Number	Average Mass	Max-10logP	Max Coverage (%)	Max# Peptides	Max# Unique	Description
563	gi|75338882|sp|Q9ZR27.1|5GT1_PERFR	50974	27.37	2	1	1	Anthocyanidin 3-O-glucoside 5-O-glucosyltransferase 1; AltName: Full=UDP-glucose:anthocyanin 5-O-glucosyltransferase 3R4; Short=p3R4; Flags: Precursor
564	gi|510785777|sp|G0LD36.1|RAS_MELOI	47161	25.33	2	1	1	Rosmarinate synthase; Short=MoRAS; AltName: Full=Hydroxycinnamoyl-CoA:hydroxyphenyllactate hydroxycinnamoyltransferase
565	gi|748013964|gb|AJE28434.1|	225117	85.49	3	7	7	Hypothetical chloroplast RF19 (chloroplast) [Premna microphylla]
566	gi|752789846|ref|YP_009117280.1|	225117	85.49	3	7	7	Hypothetical chloroplast RF19 (chloroplast) [Premna microphylla]
567	gi|751663101|gb|AJF98632.1	51505	48.13	4	2	2	Ribulose-1,5-bisphosphate carboxylase/oxygenase large subunit, partial (chloroplast) [Nepeta cataria]
568	gi|916441445|gb|AKZ23427.1|	52869	48.13	4	2	2	Ribulose-1,5-bisphosphate carboxylase/oxygenase large subunit, partial (chloroplast) [Nepeta cataria]
569	gi|751663177|gb|AJF98669.1|	51810	48.13	4	2	2	Ribulose-1,5-bisphosphate carboxylase/oxygenase large subunit, partial (chloroplast) [Stachys byzantina]
570	gi|751663109|gb|AJF98636.1|	51713	45.5	4	2	2	Ribulose-1,5-bisphosphate carboxylase/oxygenase large subunit, partial (chloroplast) [Mentha x piperita]
571	gi|817992263|gb|AKG25296.1|	52111	45.5	4	2	2	Ribulose-1,5-bisphosphate carboxylase/oxygenase large subunit, partial (plastid) [Prunella vulgaris]
572	gi|817992309|gb|AKG25319.1|	51956	45.5	4	2	2	Ribulose-1,5-bisphosphate carboxylase/oxygenase large subunit, partial (plastid) [Salvia pratensis]
573	gi|817991831|gb|AKG25080.1|	51983	45.5	4	2	2	Ribulose-1,5-bisphosphate carboxylase/oxygenase large subunit, partial (plastid) [Clinopodium vulgare]
574	gi|751663103|gb|AJF98633.1|	51988	45.5	4	2	2	Ribulose-1,5-bisphosphate carboxylase/oxygenase large subunit, partial (chloroplast) [Mentha spicata]

No.	Accession Number	Average Mass	Max-10logP	Max Coverage (%)	Max# Peptides	Max# Unique	Description
575	gi|751663105|gb|AJF98634.1|	52345	45.5	4	2	2	Ribulose-1,5-bisphosphate carboxylase/oxygenase large subunit, partial (chloroplast) [Mentha suaveolens]
576	gi|827345151|gb|AKJ76735.1|	52873	45.5	4	2	2	Ribulose-1,5-bisphosphate carboxylase/oxygenase large subunit (chloroplast) [Rosmarinus officinalis]
577	gi|836643391|ref|YP_009144523.1|	52873	45.5	4	2	2	Ribulose-1,5-bisphosphate carboxylase/oxygenase large subunit (chloroplast) [Rosmarinus officinalis]
578	gi|916441443|gb|AKZ23426.1|	52850	45.5	4	2	2	Ribulose-1,5-bisphosphate carboxylase/oxygenase large subunit (plastid) [Salvia nemorosa]
579	gi|410176163|gb|AFV61822.1|	52889	45.5	4	2	2	Ribulose-1,5-bisphosphate carboxylase/oxygenase large subunit (chloroplast) [Origanum vulgare subsp. vulgare]
580	gi|916441441|gb|AKZ23425.1|	52888	45.5	4	2	2	Ribulose-1,5-bisphosphate carboxylase/oxygenase large subunit (plastid) [Monarda fistulosa var. mollis]
581	gi|817991973|gb|AKG25151.1|	52020	45.5	4	2	2	Ribulose-1,5-bisphosphate carboxylase/oxygenase large subunit, partial (plastid) [Galeopsis bifida]
582	gi|751663168|gb|AJF98665.1|	51966	45.5	4	2	2	Ribulose-1,5-bisphosphate carboxylase/oxygenase large subunit, partial (chloroplast) [Ocimum tenuiflorum]
583	gi|817992375|gb|AKG25352.1|	52022	45.5	4	2	2	Ribulose-1,5-bisphosphate carboxylase/oxygenase large subunit, partial (plastid) [Stachys sylvatica]
584	gi|817992237|gb|AKG25283.1|	52058	45.5	4	2	2	Ribulose-1,5-bisphosphate carboxylase/oxygenase large subunit, partial (plastid) [Phlomis fruticosa]
585	gi|751663097|gb|AJF98630.1|	52095	45.5	4	2	2	Ribulose-1,5-bisphosphate carboxylase/oxygenase large subunit, partial (chloroplast) [Ocimum basilicum]
586	gi|817992335|gb|AKG25332.1|	51941	45.5	4	2	2	Ribulose-1,5-bisphosphate carboxylase/oxygenase large subunit, partial (plastid) [Scutellaria galericulata]

No.	Accession Number	Average Mass	Max-10logP	Max Coverage (%)	Max# Peptides	Max# Unique	Description
587	gi|817992071|gb|AKG25200.1|	51959	45.5	4	2	2	Ribulose-1,5-bisphosphate carboxylase/oxygenase large subunit, partial (plastid) [Lavandula x intermedia]
588	gi|817992059|gb|AKG25194.1|	51961	45.5	4	2	2	Ribulose-1,5-bisphosphate carboxylase/oxygenase large subunit, partial (plastid) [Lamium galeobdolon]
589	gi|3914545|sp|Q31655.1|RBL_AJUCH	52455	45.5	4	2	2	Ribulose bisphosphate carboxylase large chain; Short=RuBisCO large subunit; Flags: Precursor
590	gi|751663113|gb|AJF98638.1|	52702	45.5	4	2	2	Ribulose-1,5-bisphosphate carboxylase/oxygenase large subunit, partial (chloroplast) [Ajuga bracteosa]
591	gi|401879750|gb|AFQ30937.1|	52887	45.5	4	2	2	Ribulose-1,5-bisphosphate carboxylase/oxygenase large subunit (chloroplast) [Salvia miltiorrhiza]
592	gi|916441453|gb|AKZ23431.1|	53169	45.5	4	2	2	Ribulose-1,5-bisphosphate carboxylase/oxygenase large subunit (plastid) [Teucrium canadense]
593	gi|910312608|ref|YP_009162269.1|	53663	45.5	4	2	2	Ribulose-1,5-bisphosphate carboxylase/oxygenase large subunit (chloroplast) [Scutellaria baicalensis]
594	gi|827345848|gb|AKJ77149.1|	53663	45.5	4	2	2	Ribulose-1,5-bisphosphate carboxylase/oxygenase large subunit (chloroplast) [Scutellaria baicalensis]
595	gi|827346558|gb|AKJ77744.1|	53955	45.5	4	2	2	Ribulose-1 (chloroplast) [Perilla frutescens]
596	gi|573461959|emb|CCQ71628.1|	53698	45.5	4	2	2	Ribulose-1 (chloroplast) [Salvia miltiorrhiza]
597	gi|1352807|sp|P36485.2|RBL_SALDI	52005	42.25	4	2	2	Ribulose bisphosphate carboxylase large chain; Short=RuBisCO large subunit
598	gi|3914571|sp|Q33600.1|RBL_LAVLA	52404	42.25	4	2	2	Ribulose bisphosphate carboxylase large chain; Short=RuBisCO large subunit
599	gi|132044|sp|P28453.1|RBL_SCUBO	51794	42.25	4	2	2	Ribulose bisphosphate carboxylase large chain; Short=RuBisCO large subunit

No.	Accession Number	Average Mass	Max-10logP	Max Coverage (%)	Max# Peptides	Max# Unique	Description
600	gi|599079565|dbj|BAO57028.1|	49007	44.57	4	2	2	Ribulose-1,5-bisphosphate carboxylase/oxygenase large subunit, partial (chloroplast) [Sideritis cretica subsp. spicata]
601	gi|817992077|gb|AKG25203.1|	49757	44.57	4	2	2	Ribulose-1,5-bisphosphate carboxylase/oxygenase large subunit, partial (plastid) [Leonurus cardiaca]
602	gi|817992301|gb|AKG25315.1|	49431	41.41	4	2	2	Ribulose-1,5-bisphosphate carboxylase/oxygenase large subunit, partial (plastid) [Rosmarinus officinalis]
603	gi|602690611|gb|AHN96261.1|	27410	41.41	7	2	2	Ribulose-1,5-bisphosphatecarboxylase/oxygenase large subunit, partial (chloroplast) [Zhumeria majdae]
604	gi|602690609|gb|AHN96260.1|	27410	41.41	7	2	2	Ribulose-1,5-bisphosphatecarboxylase/oxygenase large subunit, partial (chloroplast) [Zhumeria majdae]
605	gi|602690607|gb|AHN96259.1|	27410	41.41	7	2	2	Ribulose-1,5-bisphosphatecarboxylase/oxygenase large subunit, partial (chloroplast) [Zhumeria majdae]
606	gi|602690629|gb|AHN96270.1|	27410	41.41	7	2	2	Ribulose-1,5-bisphosphatecarboxylase/oxygenase large subunit, partial (chloroplast) [Zhumeria majdae]
607	gi|602690623|gb|AHN96267.1|	27409	41.41	7	2	2	Ribulose-1,5-bisphosphatecarboxylase/oxygenase large subunit, partial (chloroplast) [Zhumeria majdae]
608	gi|602690619|gb|AHN96265.1|	27409	41.41	7	2	2	Ribulose-1,5-bisphosphatecarboxylase/oxygenase large subunit, partial (chloroplast) [Zhumeria majdae]
609	gi|602690617|gb|AHN96264.1|	27409	41.41	7	2	2	Ribulose-1,5-bisphosphatecarboxylase/oxygenase large subunit, partial (chloroplast) [Zhumeria majdae]
610	gi|602690627|gb|AHN96269.1|	27428	41.41	7	2	2	Ribulose-1,5-bisphosphatecarboxylase/oxygenase large subunit, partial (chloroplast) [Zhumeria majdae]
611	gi|602690625|gb|AHN96268.1|	27428	41.41	7	2	2	Ribulose-1,5-bisphosphatecarboxylase/oxygenase large subunit, partial (chloroplast) [Zhumeria majdae]

No.	Accession Number	Average Mass	Max-10logP	Max Coverage (%)	Max# Peptides	Max# Unique	Description
612	gi|602690615|gb|AHN96263.1|	27394	41.41	7	2	2	Ribulose-1,5-bisphosphatecarboxylase/oxygenase large subunit, partial (chloroplast) [Zhumeria majdae]
613	gi|602690613|gb|AHN96262.1|	27394	41.41	7	2	2	Ribulose-1,5-bisphosphatecarboxylase/oxygenase large subunit, partial (chloroplast) [Zhumeria majdae]
614	gi|602690621|gb|AHN96266.1|	27394	41.41	7	2	2	Ribulose-1,5-bisphosphatecarboxylase/oxygenase large subunit, partial (chloroplast) [Zhumeria majdae]
615	gi|751663115|gb|AJF98639.1|	27926	41.41	7	2	2	Ribulose-1,5-bisphosphatecarboxylase/oxygenase large subunit, partial (chloroplast) [Zhumeria majdae]
616	gi|602690633|gb|AHN96272.1|	27525	41.41	7	2	2	Ribulose-1,5-bisphosphatecarboxylase/oxygenase large subunit, partial (chloroplast) [Zhumeria majdae]
617	gi|602690631|gb|AHN96271.1|	27525	41.41	7	2	2	Ribulose-1,5-bisphosphatecarboxylase/oxygenase large subunit, partial (chloroplast) [Zhumeria majdae]
618	gi|602690635|gb|AHN96273.1|	27525	41.41	7	2	2	Ribulose-1,5-bisphosphatecarboxylase/oxygenase large subunit, partial (chloroplast) [Zhumeria majdae]
619	gi|752789794|ref|YP_009117229.1|	53224	41.41	4	2	2	Ribulose-1,5-bisphosphate carboxylase/oxygenase large subunit (chloroplast) [Premna microphylla]
620	gi|748013912|gb|AJE28382.1|	53224	41.41	4	2	2	Ribulose-1,5-bisphosphate carboxylase/oxygenase large subunit (chloroplast) [Premna microphylla]
621	gi|599079563|dbj|BAO57027.1|	48957	41.41	4	2	2	Ribulose-1,5-bisphosphate carboxylase/oxygenase large subunit, partial (chloroplast) [Teucrium heterophyllum]
622	gi|910312590|ref|YP_009162251.1|	158800	40.49	1	2	2	RNA polymerase beta subunit-2 (chloroplast) [Scutellaria baicalensis]
623	gi|827345829|gb|AKJ77130.1|	158800	40.49	1	2	2	RNA polymerase beta subunit-2 (chloroplast) [Scutellaria baicalensis]

No.	Accession Number	Average Mass	Max-10logP	Max Coverage (%)	Max# Peptides	Max# Unique	Description
624	gi|827346527|gb|AKJ77713.1|	158467	35.47	2	2	2	RNA polymerase beta'' subunit (chloroplast) [Perilla frutescens]
625	gi|916442767|gb|AKZ23937.1|	155842	35.47	2	2	2	RNA polymerase beta'' subunit (plastid) [Teucrium canadense]
626	gi|669254293|gb|AII20586.1|	79081	31.94	1	1	1	NADH dehydrogenase subunit F, partial (chloroplast) [Hymenopyramis cana]
627	gi|521953395|gb|AGQ04156.1|	82464	28.81	2	1	1	4-hydroxy-3-methylbut-2-enyl diphosphate synthase [Lavandula angustifolia]
628	gi|735679295|gb|AJA39985.1|	82378	28.81	2	1	1	(E)-4-hydroxy-3-methylbut-2-enyl diphosphate synthase [Salvia miltiorrhiza f. alba]
629	gi|796406070|gb|AKA59790.1|	28575	24.94	4	1	1	MYB19 [Scutellaria playfairii]
630	gi|661525316|gb|AIE15765.1|	184302	21.75	1	1	1	Dicer-like protein 3 [Salvia miltiorrhiza]
631	gi|910312663|ref|YP_009162324.1|	268069	21.56	0	1	1	Hypothetical chloroplast RF2 (chloroplast) [Scutellaria baicalensis]
632	gi|827345826|gb|AKJ77127.1|	268069	21.56	0	1	1	Hypothetical chloroplast RF2 (chloroplast) [Scutellaria baicalensis]
633	gi|827345827|gb|AKJ77128.1|	268069	21.56	0	1	1	Hypothetical chloroplast RF2 (chloroplast) [Scutellaria baicalensis]
634	gi|910312644|ref|YP_009162305.1|	268069	21.56	0	1	1	Hypothetical chloroplast RF2 (chloroplast) [Scutellaria baicalensis]
635	gi|670606706|gb|AII31147.1|	9275	20.48	11	1	1	Adenosine kinase, partial [Micromeria hyssopifolia var. hyssopifolia]
636	gi|670606710|gb|AII31149.1|	9520	20.48	10	1	1	Adenosine kinase, partial [Micromeria hyssopifolia var. kuegleri]

No.	Accession Number	Average Mass	Max-10logP	Max Coverage (%)	Max# Peptides	Max# Unique	Description
637	gi|670606712|gb|AII31150.1|	10786	20.48	9	1	1	Adenosine kinase, partial [Micromeria teneriffae var. cordifolia]
638	gi|670606714|gb|AII31151.1|	11074	20.48	9	1	1	Adenosine kinase, partial [Micromeria teneriffae var. cordifolia]
639	gi|670606704|gb|AII31146.1|	10951	20.48	9	1	1	Adenosine kinase, partial [Micromeria lepida subsp. lepida]
640	gi|670606708|gb|AII31148.1|	11164	20.48	9	1	1	Adenosine kinase, partial [Micromeria hyssopifolia var. kuegleri]
641	gi|670606716|gb|AII31152.1|	11349	20.48	9	1	1	Adenosine kinase, partial [Micromeria hyssopifolia var. glabrescens]
642	gi|670606702|gb|AII31145.1|	11506	20.48	9	1	1	Adenosine kinase, partial [Micromeria hyssopifolia var. glabrescens]
643	gi|75129878|sp|Q6WAU0.1|PULR_MENPI	37915	20.03	2	1	1	(+)-pulegone reductase
644	gi|75293243|sp|Q6WKZ0.1|C7D94_MENGR	56308	192.9	51	41	39	Cytochrome P450 71D94
645	gi|122233627|sp|Q4JF75.1|RBR_SCUBA	111795	173.76	17	23	23	Retinoblastoma-related protein
646	gi|122249145|sp|Q49SP3.1|TPSPS_POGCB	64199	115.16	7	6	6	Patchoulol synthase; Short=PatTps177; AltName: Full=Alpha-guaiene synthase; AltName: Full=Delta-guaiene synthase
647	gi|75251477|sp|Q5SBP0.1|TPSD_OCIBA	70000	111	6	6	6	Terpinolene synthase, chloroplastic; Flags: Precursor
648	gi|75129878|sp|Q6WAU0.1|PULR_MENPI	37915	107.88	8	4	4	(+)-pulegone reductase
649	gi|75252096|sp|Q5W283.1|TPSCM_MENPI	63839	88.26	6	4	4	Cis-muuroladiene synthase; Short=MxpSS1
650	gi|75290511|sp|Q6IV13.1|C7D95_MENSP	56322	81.07	4	2	1	Cytochrome P450 71D95; AltName: Full=Limonene-3-hydroxylase

No.	Accession Number	Average Mass	Max-10logP	Max Coverage (%)	Max# Peptides	Max# Unique	Description
651	gi|75293242|sp|Q6WKY9.1|C7D95_MENGR	56365	81.07	4	2	1	Cytochrome P450 71D95; AltName: Full=(-)-(4S)-Limonene-3-hydroxylase
652	gi|75161989|sp|Q8W1W9.1|5MAT1_SALSN	50724	46.62	2	1	1	Malonyl-coenzyme:anthocyanin 5-O-glucoside-6'''-O-malonyltransferase; Short=Malonyl CoA:anthocyanin 5-O-glucoside-6'''-O-malonyltransferase; Short=Ss5MaT1
653	gi|62899675|sp|O81192.1|BPPS_SALOF	69292	41.02	3	1	1	(+)-bornyl diphosphate synthase, chloroplastic; Short=BPPS; AltName: Full=(+)-alpha-pinene synthase; AltName: Full=(+)-camphene synthase; AltName: Full=SBS; Flags: Precursor
654	gi|75251483|sp|Q5SBP6.1|GDS_OCIBA	63395	40.58	2	1	1	Germacrene-D synthase; AltName: Full=(-)-germacrene D synthase
655	gi|748013968|gb|AJE28438.1|	268103	46.32	1	1	1	Hypothetical chloroplast RF21 (chloroplast) [Premna microphylla]
656	gi|752789850|ref|YP_009117284.1|	268103	46.32	1	1	1	Hypothetical chloroplast RF21 (chloroplast) [Premna microphylla]
657	gi|752789831|ref|YP_009117265.1|	268103	46.32	1	1	1	Hypothetical chloroplast RF21 (chloroplast) [Premna microphylla]
658	gi|748013949|gb|AJE28419.1|	268103	46.32	1	1	1	Hypothetical chloroplast RF21 (chloroplast) [Premna microphylla]
659	gi|573462016|emb|CCQ71685.1|	267080	43.64	1	1	1	Ycf2 (chloroplast) [Salvia miltiorrhiza]
660	gi|573461995|emb|CCQ71664.1|	267080	43.64	1	1	1	Ycf2 (chloroplast) [Salvia miltiorrhiza]
661	gi|401879785|gb|AFQ30972.1|	267080	43.64	1	1	1	Hypothetical chloroplast RF2 (chloroplast) [Salvia miltiorrhiza]
662	gi|401879806|gb|AFQ30993.1|	267080	43.64	1	1	1	Hypothetical chloroplast RF2 (chloroplast) [Salvia miltiorrhiza]
663	gi|827346579|gb|AKJ77765.1|	266476	43.64	1	1	1	Ycf2 (chloroplast) [Perilla frutescens]

No.	Accession Number	Average Mass	Max-10logP	Max Coverage (%)	Max# Peptides	Max# Unique	Description
664	gi|827346597|gb|AKJ77783.1|	266476	43.64	1	1	1	Ycf2 (chloroplast) [Perilla frutescens]
665	gi|836643370|ref|YP_009144558.1|	266537	40.53	1	1	1	Ycf2 (chloroplast) [Rosmarinus officinalis]
666	gi|827345131|gb|AKJ76715.1|	266537	40.53	1	1	1	Ycf2 (chloroplast) [Rosmarinus officinalis]
667	gi|827345130|gb|AKJ76714.1|	266537	40.53	1	1	1	Ycf2 (chloroplast) [Rosmarinus officinalis]
668	gi|836643371|ref|YP_009144577.1|	266537	40.53	1	1	1	Ycf2 (chloroplast) [Rosmarinus officinalis]
669	gi|410176216|gb|AFV61875.1|	264781	40.53	1	1	1	Ycf2 (chloroplast) [Origanum vulgare subsp. vulgare]
670	gi|410176197|gb|AFV61856.1|	264781	40.53	1	1	1	Ycf2 (chloroplast) [Origanum vulgare subsp. vulgare]
671	gi|827345827|gb|AKJ77128.1|	268069	43.09	1	1	1	Hypothetical chloroplast RF2 (chloroplast) [Scutellaria baicalensis]
672	gi|827345826|gb|AKJ77127.1|	268069	43.09	1	1	1	Hypothetical chloroplast RF2 (chloroplast) [Scutellaria baicalensis]
673	gi|910312644|ref|YP_009162305.1|	268069	43.09	1	1	1	Hypothetical chloroplast RF2 (chloroplast) [Scutellaria baicalensis]
674	gi|661525316|gb|AIE15765.1|	184302	31.49	1	1	1	Dicer-like protein 3 [Salvia miltiorrhiza]
675	gi|75129878|sp|Q6WAU0.1|PULR_MENPI	37915	31.47	2	1	1	(+)-pulegone reductase
676	gi|844572722|gb|AKN09568.1|	41656	24.97	2	1	1	Basic helix-loop-helix transcription factor [Salvia miltiorrhiza]
677	gi|395484497|gb|AFN66507.1|	77873	24.31	2	1	1	NADH dehydrogenase subunit F, partial (plastid) [Teucrium fruticans]
678	gi|395484493|gb|AFN66505.1|	77203	24.31	2	1	1	NADH dehydrogenase subunit F, partial (plastid) [Teucrium pseudochamaepitys]
679	gi|395484487|gb|AFN66503.1|	77172	24.31	2	1	1	NADH dehydrogenase subunit F, partial (plastid) [Teucrium aroanium]

No.	Accession Number	Average Mass	Max-10logP	Max Coverage (%)	Max# Peptides	Max# Unique	Description
680	gi|395484509|gb|AFN66512.1|	77251	24.31	2	1	1	NADH dehydrogenase subunit F, partial (plastid) [Teucrium orientale subsp. gloeotrichum]
681	gi|395484485|gb|AFN66502.1|	77595	24.31	2	1	1	NADH dehydrogenase subunit F, partial (plastid) [Teucrium aristatum]
682	gi|836643377|ref|YP_009144562.1|	84218	24.31	1	1	1	NADH dehydrogenase subunit 5 (chloroplast) [Rosmarinus officinalis]
683	gi|827345137|gb|AKJ76721.1|	84218	24.31	1	1	1	NADH dehydrogenase subunit 5 (chloroplast) [Rosmarinus officinalis]
684	gi|916438885|gb|AKZ22147.1|	84443	24.31	1	1	1	NADH dehydrogenase subunit 5 (plastid) [Salvia nemorosa]
685	gi|395484473|gb|AFN66496.1|	78531	24.31	2	1	1	NADH dehydrogenase subunit F, partial (plastid) [Teucridium parvifolium]
686	gi|827346594|gb|AKJ77780.1|	84248	24.31	1	1	1	NADH dehydrogenase subunit 5 (chloroplast) [Perilla frutescens]
687	gi|395484489|gb|AFN66504.1|	76910	24.31	2	1	1	NADH dehydrogenase subunit F, partial (plastid) [Teucrium betonicum]
688	gi|395484515|gb|AFN66515.1|	76501	24.31	2	1	1	NADH dehydrogenase subunit F, partial (plastid) [Teucrium racemosum]
689	gi|395484524|gb|AFN66519.1|	75723	24.31	2	1	1	NADH dehydrogenase subunit F, partial (plastid) [Teucrium kotschyanum]
690	gi|395484517|gb|AFN66516.1|	76688	24.31	2	1	1	NADH dehydrogenase subunit F, partial (plastid) [Teucrium salviastrum]
691	gi|395484511|gb|AFN66513.1|	76860	24.31	2	1	1	NADH dehydrogenase subunit F, partial (plastid) [Teucrium oxylepis]
692	gi|401879790|gb|AFQ30977.1|	83587	24.31	1	1	1	NADH dehydrogenase subunit 5 (chloroplast) [Salvia miltiorrhiza]

No.	Accession Number	Average Mass	Max-10logP	Max Coverage (%)	Max# Peptides	Max# Unique	Description
693	gi|573462000|emb|CCQ71669.1|	83587	24.31	1	1	1	NADH dehydrogenase subunit 5 (chloroplast) [Salvia miltiorrhiza]
694	gi|395484522|gb|AFN66518.1|	76105	24.31	2	1	1	NADH dehydrogenase subunit F, partial (plastid) [Teucrium subspinosum]
695	gi|752789835|ref|YP_009117269.1|	85385	24.31	1	1	1	NADH-plastoquinone oxidoreductase subunit 5 (chloroplast) [Premna microphylla]
696	gi|748013953|gb|AJE28423.1|	85385	24.31	1	1	1	NADH-plastoquinone oxidoreductase subunit 5 (chloroplast) [Premna microphylla]
697	gi|395484475|gb|AFN66497.1|	78510	24.31	2	1	1	NADH dehydrogenase subunit F, partial (plastid) [Rubiteucris palmata]
698	gi|916438887|gb|AKZ22148.1|	83254	24.31	1	1	1	NADH dehydrogenase subunit 5 (plastid) [Teucrium canadense]
699	gi|395484502|gb|AFN66509.1|	75956	24.31	2	1	1	NADH dehydrogenase subunit F, partial (plastid) [Teucrium divaricatum]
700	gi|395484481|gb|AFN66500.1|	76061	24.31	2	1	1	NADH dehydrogenase subunit F, partial (plastid) [Teucrium flavum subsp. glaucum]
701	gi|395484520|gb|AFN66517.1|	76892	24.31	2	1	1	NADH dehydrogenase subunit F, partial (plastid) [Teucrium spinosum]
702	gi|395484477|gb|AFN66498.1|	76293	24.31	2	1	1	NADH dehydrogenase subunit F, partial (plastid) [Spartothamnella puberula]
703	gi|395484483|gb|AFN66501.1|	77431	24.31	2	1	1	NADH dehydrogenase subunit F, partial (plastid) [Teucrium albicaule]
704	gi|669254293|gb|AII20586.1|	79081	24.31	2	1	1	NADH dehydrogenase subunit F, partial (chloroplast) [Hymenopyramis cana]
705	gi|669254291|gb|AII20585.1|	79070	24.31	2	1	1	NADH dehydrogenase subunit F, partial (chloroplast) [Petraeovitex multiflora]

No.	Accession Number	Average Mass	Max-10logP	Max Coverage (%)	Max# Peptides	Max# Unique	Description
706	gi|916438881|gb|AKZ22145.1|	84354	24.31	1	1	1	NADH dehydrogenase subunit 5 (plastid) [Monarda fistulosa var. mollis]
707	gi|410176201|gb|AFV61860.1|	84295	24.31	1	1	1	NADH dehydrogenase subunit 5 (chloroplast) [Origanum vulgare subsp. vulgare]
708	gi|395484537|gb|AFN66525.1|	78210	24.31	2	1	1	NADH dehydrogenase subunit F, partial (plastid) [Teucrium decipiens]
709	gi|395484530|gb|AFN66522.1|	76725	24.31	2	1	1	NADH dehydrogenase subunit F, partial (plastid) [Teucrium viscidum var. miquelianum]
710	gi|395484528|gb|AFN66521.1|	76705	24.31	2	1	1	NADH dehydrogenase subunit F, partial (plastid) [Teucrium japonicum]
711	gi|395484507|gb|AFN66511.1|	77434	24.31	2	1	1	NADH dehydrogenase subunit F, partial (plastid) [Teucrium nudicaule]
712	gi|395484504|gb|AFN66510.1|	77454	24.31	2	1	1	NADH dehydrogenase subunit F, partial (plastid) [Teucrium laciniatum]
713	gi|395484526|gb|AFN66520.1|	76596	24.31	2	1	1	NADH dehydrogenase subunit F, partial (plastid) [Teucrium bicolor]
714	gi|827345833|gb|AKJ77134.1|	85675	24.31	1	1	1	NADH dehydrogenase subunit 5 (chloroplast) [Scutellaria baicalensis]
715	gi|910312648|ref|YP_009162309.1|	85675	24.31	1	1	1	NADH dehydrogenase subunit 5 (chloroplast) [Scutellaria baicalensis]
716	gi|669254285|gb|AII20582.1|	74815	24.31	2	1	1	NADH dehydrogenase subunit F, partial (chloroplast) [Holocheila longipedunculata]
717	gi|669254287|gb|AII20583.1|	77022	24.31	2	1	1	NADH dehydrogenase subunit F, partial (chloroplast) [Holocheila longipedunculata]
718	gi|669254283|gb|AII20581.1|	77991	24.31	2	1	1	NADH dehydrogenase subunit F, partial (chloroplast) [Holocheila longipedunculata]

No.	Accession Number	Average Mass	Max-10logP	Max Coverage (%)	Max# Peptides	Max# Unique	Description
719	gi|395484513|gb|AFN66514.1|	76814	24.31	2	1	1	NADH dehydrogenase subunit F, partial (plastid) [Teucrium pyrenaicum]
720	gi|395484495|gb|AFN66506.1|	76993	24.31	2	1	1	NADH dehydrogenase subunit F, partial (plastid) [Teucrium eriocephalum subsp. almeriense]
721	gi|395484500|gb|AFN66508.1|	77065	24.31	2	1	1	NADH dehydrogenase subunit F, partial (plastid) [Teucrium stocksianum subsp. incanum]
722	gi|395484479|gb|AFN66499.1|	34714	24.31	4	1	1	NADH dehydrogenase subunit F, partial (plastid) [Oncinocalyx betchei]
723	gi|395484533|gb|AFN66523.1|	75763	24.31	2	1	1	NADH dehydrogenase subunit F, partial (plastid) [Teucrium montbretii subsp. heliotropiifolium]
724	gi|395484535|gb|AFN66524.1|	76413	24.31	2	1	1	NADH dehydrogenase subunit F, partial (plastid) [Teucrium antitauricum]
725	gi|510794472|gb|AGN52202.1|	40938	24.14	2	1	1	MYB-related transcription factor [Salvia miltiorrhiza]
726	gi|573462009|emb|CCQ71678.1|	45534	23.18	4	1	1	NADH dehydrogenase subunit 7 (chloroplast) [Salvia miltiorrhiza]
727	gi|401879799|gb|AFQ30986.1|	45534	23.18	4	1	1	NADH dehydrogenase subunit 7 (chloroplast) [Salvia miltiorrhiza]
728	gi|836643397|ref|YP_009144571.1|	45642	23.18	4	1	1	NADH dehydrogenase subunit 7 (chloroplast) [Rosmarinus officinalis]
729	gi|827345157|gb|AKJ76741.1|	45642	23.18	4	1	1	NADH dehydrogenase subunit 7 (chloroplast) [Rosmarinus officinalis]
730	gi|410176210|gb|AFV61869.1|	45519	23.18	4	1	1	NADH dehydrogenase subunit 7 (chloroplast) [Origanum vulgare subsp. vulgare]
731	gi|916439041|gb|AKZ22225.1|	45538	23.18	4	1	1	NADH dehydrogenase subunit 7 (plastid) [Monarda fistulosa var. mollis]

No.	Accession Number	Average Mass	Max-10logP	Max Coverage (%)	Max# Peptides	Max# Unique	Description
732	gi|916439045|gb|AKZ22227.1|	45533	23.18	4	1	1	NADH dehydrogenase subunit 7 (plastid) [Salvia nemorosa]
733	gi|916439043|gb|AKZ22226.1|	45734	23.18	4	1	1	NADH dehydrogenase subunit 7 (plastid) [Nepeta cataria]
734	gi|916439047|gb|AKZ22228.1|	45476	23.18	4	1	1	NADH dehydrogenase subunit 7 (plastid) [Teucrium canadense]
735	gi|827346580|gb|AKJ77766.1|	45539	23.18	4	1	1	NADH dehydrogenase subunit 7 (chloroplast) [Perilla frutescens]
736	gi|827345853|gb|AKJ77154.1|	45491	23.18	4	1	1	NADH dehydrogenase subunit 7 (chloroplast) [Scutellaria baicalensis]
737	gi|910312657|ref|YP_009162318.1|	45491	23.18	4	1	1	NADH dehydrogenase subunit 7 (chloroplast) [Scutellaria baicalensis]
738	gi|752789844|ref|YP_009117278.1|	45401	23.18	4	1	1	NADH-plastoquinone oxidoreductase subunit 7 (chloroplast) [Premna microphylla]
739	gi|748013962|gb|AJE28432.1|	45401	23.18	4	1	1	NADH-plastoquinone oxidoreductase subunit 7 (chloroplast) [Premna microphylla]
740	gi|748013960|gb|AJE28430.1|	19533	21.73	6	1	1	NADH-plastoquinone oxidoreductase subunit I (chloroplast) [Premna microphylla]
741	gi|752789842|ref|YP_009117276.1|	19533	21.73	6	1	1	NADH-plastoquinone oxidoreductase subunit I (chloroplast) [Premna microphylla]
742	gi|916438099|gb|AKZ21755.1|	37677	21.34	2	1	1	cytochrome c biogenesis protein (plastid) [Nepeta cataria]
743	gi|916442749|gb|AKZ23931.1|	157974	21.28	1	1	1	RNA polymerase beta'' subunit (plastid) [Salvia nemorosa]
744	gi|836643373|ref|YP_009144505.1|	158291	21.28	1	1	1	RNA polymerase beta'' subunit (chloroplast) [Rosmarinus officinalis]

No.	Accession Number	Average Mass	Max-10logP	Max Coverage (%)	Max# Peptides	Max# Unique	Description
745	gi|827345133|gb|AKJ76717.1|	158291	21.28	1	1	1	RNA polymerase beta'' subunit (chloroplast) [Rosmarinus officinalis]
746	gi|410176145|gb|AFV61804.1|	158559	21.28	1	1	1	RNA polymerase beta'' subunit (chloroplast) [Origanum vulgare subsp. vulgare]
747	gi|916442746|gb|AKZ23930.1|	158642	21.28	1	1	1	RNA polymerase beta'' subunit (plastid) [Monarda fistulosa var. mollis]
748	gi|75244696|sp|Q8H2B4.1|LLOS_MENAQ	267080	137.47	5	14	14	R-linalool synthase, chloroplastic; Flags: Precursor
749	gi|75251477|sp|Q5SBP0.1|TPSD_OCIBA	267080	137.47	5	14	14	Terpinolene synthase, chloroplastic; Flags: Precursor
750	gi|8134569|sp|Q42662.2|METE_PLESU	267080	137.19	5	14	14	5-methyltetrahydropteroyltriglutamate--homocysteine methyltransferase; AltName: Full=Cobalamin-independent methionine synthase isozyme; AltName: Full=Vitamin-B12-independent methionine synthase isozyme
751	gi|75251482|sp|Q5SBP5.1|GCS1_OCIBA	267080	137.47	5	14	14	Gamma-cadinene synthase; AltName: Full=(+)-gamma-cadinene synthase
752	gi|122249145|sp|Q49SP3.1|TPSPS_POGCB	78531	34.94	2	1	1	Patchoulol synthase; Short=PatTps177; AltName: Full=Alpha-guaiene synthase; AltName: Full=Delta-guaiene synthase
753	gi|75251481|sp|Q5SBP4.1|AZIS_OCIBA	76501	34.94	2	1	1	Alpha-zingiberene synthase
754	gi|75129878|sp|Q6WAU0.1|PULR_MENPI	84218	34.94	1	1	1	(+)-pulegone reductase
755	gi|122210942|sp|Q2XSC4.1|LABER_LAVAN	84218	34.94	1	1	1	Exo-alpha-bergamotene synthase; Short=LaBERS; AltName: Full=Trans-alpha-bergamotene synthase
756	gi|122200954|sp|Q2KNL5.1|CADH1_OCIBA	84443	34.94	1	1	1	Cinnamyl alcohol dehydrogenase 1; Short=CAD 1; Short=ObaCAD1
757	gi|122219295|sp|Q49SP7.1|TPSCS_POGCB	77203	34.94	2	1	1	Gamma-curcumene synthase; AltName: Full=PatTpsA

No.	Accession Number	Average Mass	Max-10logP	Max Coverage (%)	Max# Peptides	Max# Unique	Description
758	gi|122233627|sp|Q4JF75.1|RBR_SCUBA	84354	34.94	1	1	1	Retinoblastoma-related protein
759	gi|75161989|sp|Q8W1W9.1|5MAT1_SALSN	79081	34.94	2	1	1	Malonyl-coenzyme:anthocyanin 5-O-glucoside-6'''-O-malonyltransferase; Short=Malonyl CoA:anthocyanin 5-O-glucoside-6'''-O-malonyltransferase; Short=Ss5MaT1
760	gi|510785777|sp|G0LD36.1|RAS_MELOI	84295	34.94	1	1	1	Rosmarinate synthase; Short=MoRAS; AltName: Full=Hydroxycinnamoyl-CoA:hydroxyphenyllactate hydroxycinnamoyltransferase
761	gi|573461995|emb|CCQ71664.1|	267080	137.47	5	14	14	Ycf2 (chloroplast) [Salvia miltiorrhiza]
762	gi|573462016|emb|CCQ71685.1|	267080	137.47	5	14	14	Ycf2 (chloroplast) [Salvia miltiorrhiza]
763	gi|401879785|gb|AFQ30972.1|	267080	137.47	5	14	14	Hypothetical chloroplast RF2 (chloroplast) [Salvia miltiorrhiza]
764	gi|401879806|gb|AFQ30993.1|	267080	137.47	5	14	14	Hypothetical chloroplast RF2 (chloroplast) [Salvia miltiorrhiza]
765	gi|395484473|gb|AFN66496.1|	78531	34.94	2	1	1	NADH dehydrogenase subunit F, partial (plastid) [Teucridium parvifolium]
766	gi|395484515|gb|AFN66515.1|	76501	34.94	2	1	1	NADH dehydrogenase subunit F, partial (plastid) [Teucrium racemosum]
767	gi|836643377|ref|YP_009144562.1|	84218	34.94	1	1	1	NADH dehydrogenase subunit 5 (chloroplast) [Rosmarinus officinalis]
768	gi|827345137|gb|AKJ76721.1|	84218	34.94	1	1	1	NADH dehydrogenase subunit 5 (chloroplast) [Rosmarinus officinalis]
769	gi|916438885|gb|AKZ22147.1|	84443	34.94	1	1	1	NADH dehydrogenase subunit 5 (plastid) [Salvia nemorosa]
770	gi|395484493|gb|AFN66505.1|	77203	34.94	2	1	1	NADH dehydrogenase subunit F, partial (plastid) [Teucrium pseudochamaepitys]

No.	Accession Number	Average Mass	Max-10logP	Max Coverage (%)	Max# Peptides	Max# Unique	Description
771	gi|916438881|gb|AKZ22145.1|	84354	34.94	1	1	1	NADH dehydrogenase subunit 5 (plastid) [Monarda fistulosa var. mollis]
772	gi|669254293|gb|AII20586.1|	79081	34.94	2	1	1	NADH dehydrogenase subunit F, partial (chloroplast) [Hymenopyramis cana]
773	gi|410176201|gb|AFV61860.1|	84295	34.94	1	1	1	NADH dehydrogenase subunit 5 (chloroplast) [Origanum vulgare subsp. vulgare]
774	gi|395484517|gb|AFN66516.1|	76688	34.94	2	1	1	NADH dehydrogenase subunit F, partial (plastid) [Teucrium salviastrum]
775	gi|395484511|gb|AFN66513.1|	76860	34.94	2	1	1	NADH dehydrogenase subunit F, partial (plastid) [Teucrium oxylepis]
776	gi|395484489|gb|AFN66504.1|	76910	34.94	2	1	1	NADH dehydrogenase subunit F, partial (plastid) [Teucrium betonicum]
777	gi|669254285|gb|AII20582.1|	74815	34.94	2	1	1	NADH dehydrogenase subunit F, partial (chloroplast) [Holocheila longipedunculata]
778	gi|669254287|gb|AII20583.1|	77022	34.94	2	1	1	NADH dehydrogenase subunit F, partial (chloroplast) [Holocheila longipedunculata]
779	gi|669254283|gb|AII20581.1|	77991	34.94	2	1	1	NADH dehydrogenase subunit F, partial (chloroplast) [Holocheila longipedunculata]
780	gi|395484483|gb|AFN66501.1|	77431	34.94	2	1	1	NADH dehydrogenase subunit F, partial (plastid) [Teucrium albicaule]
781	gi|395484520|gb|AFN66517.1|	76892	34.94	2	1	1	NADH dehydrogenase subunit F, partial (plastid) [Teucrium spinosum]
782	gi|395484495|gb|AFN66506.1|	76993	34.94	2	1	1	NADH dehydrogenase subunit F, partial (plastid) [Teucrium eriocephalum subsp. almeriense]
783	gi|395484500|gb|AFN66508.1|	77065	34.94	2	1	1	NADH dehydrogenase subunit F, partial (plastid) [Teucrium stocksianum subsp. incanum]

No.	Accession Number	Average Mass	Max-10logP	Max Coverage (%)	Max# Peptides	Max# Unique	Description
784	gi|395484526|gb|AFN66520.1|	76596	34.94	2	1	1	NADH dehydrogenase subunit F, partial (plastid) [Teucrium bicolor]
785	gi|395484507|gb|AFN66511.1|	77434	34.94	2	1	1	NADH dehydrogenase subunit F, partial (plastid) [Teucrium nudicaule]
786	gi|395484504|gb|AFN66510.1|	77454	34.94	2	1	1	NADH dehydrogenase subunit F, partial (plastid) [Teucrium laciniatum]
787	gi|395484477|gb|AFN66498.1|	76293	34.94	2	1	1	NADH dehydrogenase subunit F, partial (plastid) [Spartothamnella puberula]
788	gi|395484513|gb|AFN66514.1|	76814	34.94	2	1	1	NADH dehydrogenase subunit F, partial (plastid) [Teucrium pyrenaicum]
789	gi|395484502|gb|AFN66509.1|	75956	34.94	2	1	1	NADH dehydrogenase subunit F, partial (plastid) [Teucrium divaricatum]
790	gi|395484522|gb|AFN66518.1|	76105	34.94	2	1	1	NADH dehydrogenase subunit F, partial (plastid) [Teucrium subspinosum]
791	gi|669254291|gb|AII20585.1|	79070	34.94	2	1	1	NADH dehydrogenase subunit F, partial (chloroplast) [Petraeovitex multiflora]
792	gi|395484475|gb|AFN66497.1|	78510	34.94	2	1	1	NADH dehydrogenase subunit F, partial (plastid) [Rubiteucris palmata]
793	gi|395484481|gb|AFN66500.1|	76061	34.94	2	1	1	NADH dehydrogenase subunit F, partial (plastid) [Teucrium flavum subsp. glaucum]
794	gi|916438887|gb|AKZ22148.1|	83254	34.94	1	1	1	NADH dehydrogenase subunit 5 (plastid) [Teucrium canadense]
795	gi|748013953|gb|AJE28423.1|	85385	34.94	1	1	1	NADH-plastoquinone oxidoreductase subunit 5 (chloroplast) [Premna microphylla]
796	gi|752789835|ref|YP_009117269.1|	85385	34.94	1	1	1	NADH-plastoquinone oxidoreductase subunit 5 (chloroplast) [Premna microphylla]

No.	Accession Number	Average Mass	Max-10logP	Max Coverage (%)	Max# Peptides	Max# Unique	Description
797	gi|395484530|gb|AFN66522.1|	76725	34.94	2	1	1	NADH dehydrogenase subunit F, partial (plastid) [Teucrium viscidum var. miquelianum]
798	gi|395484528|gb|AFN66521.1|	76705	34.94	2	1	1	NADH dehydrogenase subunit F, partial (plastid) [Teucrium japonicum]
799	gi|827346594|gb|AKJ77780.1|	84248	34.94	1	1	1	NADH dehydrogenase subunit 5 (chloroplast) [Perilla frutescens]
800	gi|395484497|gb|AFN66507.1|	77873	34.94	2	1	1	NADH dehydrogenase subunit F, partial (plastid) [Teucrium fruticans]
801	gi|395484509|gb|AFN66512.1|	77251	34.94	2	1	1	NADH dehydrogenase subunit F, partial (plastid) [Teucrium orientale subsp. gloeotrichum]
802	gi|395484485|gb|AFN66502.1|	77595	34.94	2	1	1	NADH dehydrogenase subunit F, partial (plastid) [Teucrium aristatum]
803	gi|395484487|gb|AFN66503.1|	77172	34.94	2	1	1	NADH dehydrogenase subunit F, partial (plastid) [Teucrium aroanium]
804	gi|395484537|gb|AFN66525.1|	78210	34.94	2	1	1	NADH dehydrogenase subunit F, partial (plastid) [Teucrium decipiens]
805	gi|573462000|emb|CCQ71669.1|	83587	26.78	1	1	1	NADH dehydrogenase subunit 5 (chloroplast) [Salvia miltiorrhiza]
806	gi|401879790|gb|AFQ30977.1|	83587	26.78	1	1	1	NADH dehydrogenase subunit 5 (chloroplast) [Salvia miltiorrhiza]
807	gi|395484533|gb|AFN66523.1|	75763	26.78	2	1	1	NADH dehydrogenase subunit F, partial (plastid) [Teucrium montbretii subsp. heliotropiifolium]
808	gi|395484535|gb|AFN66524.1|	76413	26.78	2	1	1	NADH dehydrogenase subunit F, partial (plastid) [Teucrium antitauricum]
809	gi|827345833|gb|AKJ77134.1|	85675	26.78	1	1	1	NADH dehydrogenase subunit 5 (chloroplast) [Scutellaria baicalensis]

No.	Accession Number	Average Mass	Max-10logP	Max Coverage (%)	Max# Peptides	Max# Unique	Description
810	gi|910312648|ref|YP_009162309.1|	85675	26.78	1	1	1	NADH dehydrogenase subunit 5 (chloroplast) [Scutellaria baicalensis]
811	gi|395484479|gb|AFN66499.1|	34714	26.78	4	1	1	NADH dehydrogenase subunit F, partial (plastid) [Oncinocalyx betchei]
812	gi|75129878|sp|Q6WAU0.1|PULR_MENPI	37915	34.40	2	1	1	(+)-pulegone reductase
813	gi|5915814|sp|O04164.1|C71A6_NEPRA	57955	124.01	14	7	7	Cytochrome P450 71A6
814	gi|75161989|sp|Q8W1W9.1|5MAT1_SALSN	50724	103.98	3	2	2	Malonyl-coenzyme:anthocyanin 5-O-glucoside-6'''-O-malonyltransferase; Short=Malonyl CoA:anthocyanin 5-O-glucoside-6'''-O-malonyltransferase; Short=Ss5MaT1
815	gi|75244696|sp|Q8H2B4.1|LLOS_MENAQ	70535	100.72	7	5	4	R-linalool synthase, chloroplastic; Flags: Precursor
816	gi|75129878|sp|Q6WAU0.1|PULR_MENPI	37915	86.57	6	3	3	(+)-pulegone reductase
817	gi|56749087|sp|Q85XY6.1|MATK_OCIBA	60282	75.48	6	3	3	Maturase K; AltName: Full=Intron maturase
818	gi|75251478|sp|Q5SBP1.1|MYRS_OCIBA	69964	74.79	3	2	1	Beta-myrcene synthase, chloroplastic; Flags: Precursor
819	gi|75251477|sp|Q5SBP0.1|TPSD_OCIBA	70000	65.09	5	3	2	Terpinolene synthase, chloroplastic; Flags: Precursor
820	gi|75315259|sp|Q9XHE6.1|C71DF_MENPI	56532	62.77	2	1	1	Cytochrome P450 71D15; AltName: Full=(-)-(4S)-Limonene-3-hydroxylase; AltName: Full=Cytochrome P450 isoform PM2
821	gi|510785777|sp|G0LD36.1|RAS_MELOI	47161	60.94	5	2	2	Rosmarinate synthase; Short=MoRAS; AltName: Full=Hydroxycinnamoyl-CoA:hydroxyphenyllactate hydroxycinnamoyltransferase
822	gi|29839421|sp|Q9XGW0.1|COMT1_OCIBA	39529	59.08	3	1	1	Caffeic acid 3-O-methyltransferase 1; Short=CAOMT-1; Short=COMT-1; AltName: Full=S-adenosysl-L-methionine:caffeic acid 3-O-methyltransferase 1

No.	Accession Number	Average Mass	Max-10logP	Max Coverage (%)	Max# Peptides	Max# Unique	Description
823	gi|29839420|sp|Q9XGV9.1|COMT2_OCIBA	39613	52.73	3	1	1	Caffeic acid 3-O-methyltransferase 2; Short=CAOMT-2; Short=COMT-2; AltName: Full=S-adenosysl-L-methionine:caffeic acid 3-O-methyltransferase 2
824	gi|75251482|sp|Q5SBP5.1|GCS1_OCIBA	63566	46.66	4	1	1	Gamma-cadinene synthase; AltName: Full=(+)-gamma-cadinene synthase
825	gi|75227033|sp|Q76MR7.1|UBGAT_SCUBA	48654	44.81	2	1	1	Baicalein 7-O-glucuronosyltransferase; AltName: Full=UDP-glucuronate:baicalein 7-O-glucuronosyltransferase
826	gi|401879801|gb|AFQ30988.1|	218699	34.68	1	1	1	Photosystem I assembly protein Ycf1 (chloroplast) [Salvia miltiorrhiza]
827	gi|573462011|emb|CCQ71680.1|	218685	32.29	1	1	1	Ycf1 (chloroplast) [Salvia miltiorrhiza]
828	gi|661525316|gb|AIE15765.1|	184302	32.29	1	1	1	Dicer-like protein 3 [Salvia miltiorrhiza]
829	gi|844572794|gb|AKN09591.1|	22976	26.64	4	1	1	Basic helix-loop-helix transcription factor [Salvia miltiorrhiza]
830	gi|661525312|gb|AIE15763.1|	216665	24.47	1	1	1	Dicer-like protein 1 [Salvia miltiorrhiza]
831	gi|916441767|gb|AKZ23588.1|	15470	24.02	6	1	1	Ribosomal protein L20 (plastid) [Teucrium canadense]
832	gi|827345878|gb|AKJ77179.1|	15363	24.02	6	1	1	Ribosomal protein L20 (chloroplast) [Scutellaria baicalensis]
833	gi|910312623|ref|YP_009162284.1|	15363	24.02	6	1	1	Ribosomal protein L20 (chloroplast) [Scutellaria baicalensis]
834	gi|916441755|gb|AKZ23582.1|	15554	24.02	6	1	1	Ribosomal protein L20 (plastid) [Nepeta cataria]
835	gi|916441757|gb|AKZ23583.1|	15566	24.02	6	1	1	Ribosomal protein L20 (plastid) [Salvia nemorosa]
836	gi|410176177|gb|AFV61836.1|	15446	24.02	6	1	1	Ribosomal protein L20 (chloroplast) [Origanum vulgare subsp. vulgare]

No.	Accession Number	Average Mass	Max-10logP	Max Coverage (%)	Max# Peptides	Max# Unique	Description
837	gi|916441753|gb|AKZ23581.1|	15522	24.02	6	1	1	Ribosomalprotein L20 (plastid) [Monarda fistulosa var. mollis]
838	gi|827345181|gb|AKJ76765.1|	15446	24.02	6	1	1	Ribosomal protein L20 (chloroplast) [Rosmarinus officinalis]
839	gi|836643421|ref|YP_009144537.1|	15446	24.02	6	1	1	Ribosomal protein L20 (chloroplast) [Rosmarinus officinalis]
840	gi|827346543|gb|AKJ77729.1|	15537	24.02	6	1	1	Ribosomal protein L20 (chloroplast) [Perilla frutescens]
841	gi|748013927|gb|AJE28397.1|	15724	24.02	6	1	1	Ribosomal protein L20 (chloroplast) [Premna microphylla]
842	gi|752789809|ref|YP_009117244.1|	15724	24.02	6	1	1	Ribosomal protein L20 (chloroplast) [Premna microphylla]
843	gi|573461974|emb|CCQ71643.1|	15541	24.02	6	1	1	Ribosomal protein L20 (chloroplast) [Salvia miltiorrhiza]
844	gi|401879765|gb|AFQ30952.1|	15541	24.02	6	1	1	Ribosomal protein L20 (chloroplast) [Salvia miltiorrhiza]
845	gi|75129878|sp|Q6WAU0.1|PULR_MENPI	37915	23.86	2	1	1	(+)-pulegone reductase
846	gi|745791013|gb|AJD25215.1|	56512	21.31	2	1	1	Cytochrome P450 CYP92B28 [Salvia miltiorrhiza]
847	gi|75161989|sp|Q8W1W9.1|5MAT1_SALSN	101.20	10	5	5	50724	Malonyl-coenzyme:anthocyanin 5-O-glucoside-6'''-O-malonyltransferase; Short=Malonyl CoA:anthocyanin 5-O-glucoside-6'''-O-malonyltransferase; Short=Ss5MaT1
848	gi|75129878|sp|Q6WAU0.1|PULR_MENPI	96.90	6	3	3	37915	(+)-pulegone reductase
849	gi|8134569|sp|Q42662.2|METE_PLESU	92.21	4	4	4	84590	5-methyltetrahydropteroyltriglutamate--homocysteine methyltransferase; AltName: Full=Cobalamin-independent methionine synthase isozyme; AltName: Full=Vitamin-B12-independent methionine synthase isozyme

No.	Accession Number	Average Mass	Max-10logP	Max Coverage (%)	Max# Peptides	Max# Unique	Description
850	gi|5921781|sp|O04111.1|CHSY_PERFR	88.85	2	1	1	42686	Chalcone synthase; AltName: Full=Naringenin-chalcone synthase
851	gi|75288825|sp|Q65CJ7.2|HPPR_PLESU	79.89	3	1	1	34128	Hydroxyphenylpyruvate reductase; Short=HPPR
852	gi|75180331|sp|Q9LRC8.1|BAGLU_SCUBA	77.84	4	2	2	58772	Baicalin-beta-D-glucuronidase; AltName: Full=Baicalinase; Flags: Precursor
853	gi|75219538|sp|O48935.1|TPSBF_MENPI	73.42	4	2	2	63830	Beta-farnesene synthase
854	gi|122219294|sp|Q49SP6.1|TPGD2_POGCB	72.68	2	1	1	64149	Germacrene D synthase 2; AltName: Full=PatTpsBF2
855	gi|75244696|sp|Q8H2B4.1|LLOS_MENAQ	71.83	2	1	1	70535	R-linalool synthase, chloroplastic; Flags: Precursor
856	gi|62899675|sp|O81192.1|BPPS_SALOF	70.92	2	1	1	69292	(+)-bornyl diphosphate synthase, chloroplastic; Short=BPPS; AltName: Full=(+)-alpha-pinene synthase; AltName: Full=(+)-camphene synthase; AltName: Full=SBS; Flags: Precursor
857	gi|14423898|sp|Q9M573.1|RL31_PERFR	64.2	7	1	1	13894	60S ribosomal protein L31
858	gi|75251477|sp|Q5SBP0.1|TPSD_OCIBA	59.95	2	1	1	70000	Terpinolene synthase, chloroplastic; Flags: Precursor
859	gi|75251482|sp|Q5SBP5.1|GCS1_OCIBA	41.49	4	1	1	63566	Gamma-cadinene synthase; AltName: Full=(+)-gamma-cadinene synthase
860	gi|56749087|sp|Q85XY6.1|MATK_OCIBA	40.87	2	1	1	60282	Maturase K; AltName: Full=Intron maturase
861	gi|122210943|sp|Q2XSC5.1|LALIN_LAVAN	39.51	1	1	1	65654	R-linalool synthase; Short=LaLINS
862	gi|510785777|sp|G0LD36.1|RAS_MELOI	38.69	2	1	1	47161	Rosmarinate synthase; Short=MoRAS; AltName: Full=Hydroxycinnamoyl-CoA:hydroxyphenyllactate hydroxycinnamoyltransferase
863	gi|75306222|sp|Q947B7.1|MFS_MENPI	34.67	5	1	1	55360	(+)-menthofuran synthase; AltName: Full=(+)-pulegone 9-hydroxylase

No.	Accession Number	Average Mass	Max-10logP	Max Coverage (%)	Max# Peptides	Max# Unique	Description
864	gi|410176212|gb|AFV61871.1|	217739	129.41	11	24	23	Protein Ycf1 (chloroplast) [Origanum vulgare subsp. vulgare]; Protein TIC 214
865	gi|401879801|gb|AFQ30988.1|	218699	48.04	1	2	1	Photosystem I assembly protein Ycf1 (chloroplast) [Salvia miltiorrhiza]; Protein TIC 214
866	gi|573462011|emb|CCQ71680.1|	218685	48.04	1	2	1	Protein Ycf1 (chloroplast) [Salvia miltiorrhiza]; Protein TIC 214
867	gi|661525318|gb|AIE15766.1|	183633	46.23	0	1	1	Dicer-like protein 4a [Salvia miltiorrhiza]
868	gi|75129878|sp|Q6WAU0.1|PULR_MENPI	37915	41.91	2	1	1	(+)-pulegone reductase
869	gi|410176197|gb|AFV61856.1|	264781	40.31	1	2	2	Protein Ycf2 (chloroplast) [Origanum vulgare subsp. vulgare]
870	gi|410176216|gb|AFV61875.1|	264781	40.31	1	2	2	Protein Ycf2 (chloroplast) [Origanum vulgare subsp. vulgare]
871	gi|836643370|ref|YP_009144558.1|	266537	40.31	1	2	2	Protein Ycf2 (chloroplast) [Rosmarinus officinalis]
872	gi|827345131|gb|AKJ76715.1|	266537	40.31	1	2	2	Protein Ycf2 (chloroplast) [Rosmarinus officinalis]
873	gi|827345130|gb|AKJ76714.1|	266537	40.31	1	2	2	Protein Ycf2 (chloroplast) [Rosmarinus officinalis]
874	gi|836643371|ref|YP_009144577.1|	266537	40.31	1	2	2	Protein Ycf2 (chloroplast) [Rosmarinus officinalis]
875	gi|844572640|gb|AKN09541.1|	34841	39.74	3	1	1	Basic helix-loop-helix transcription factor [Salvia miltiorrhiza]
876	gi|602690681|gb|AHN96278.1|	37139	37.81	3	1	1	Marurase K, partial (chloroplast) [Zhumeria majdae]
877	gi|602690679|gb|AHN96277.1|	37139	37.81	3	1	1	Marurase K, partial (chloroplast) [Zhumeria majdae]
878	gi|602690689|gb|AHN96282.1|	37139	37.81	3	1	1	Marurase K, partial (chloroplast) [Zhumeria majdae]
879	gi|602690701|gb|AHN96288.1|	37139	37.81	3	1	1	Marurase K, partial (chloroplast) [Zhumeria majdae]

No.	Accession Number	Average Mass	Max-10logP	Max Coverage (%)	Max# Peptides	Max# Unique	Description
880	gi|602690697|gb|AHN96286.1|	37139	37.81	3	1	1	Marurase K, partial (chloroplast) [Zhumeria majdae]
881	gi|602690673|gb|AHN96274.1|	37139	37.81	3	1	1	Marurase K, partial (chloroplast) [Zhumeria majdae]
882	gi|602690675|gb|AHN96275.1|	37139	37.81	3	1	1	Marurase K, partial (chloroplast) [Zhumeria majdae]
883	gi|602690683|gb|AHN96279.1|	37138	37.81	3	1	1	Marurase K, partial (chloroplast) [Zhumeria majdae]
884	gi|602690699|gb|AHN96287.1|	37138	37.81	3	1	1	Marurase K, partial (chloroplast) [Zhumeria majdae]
885	gi|602690687|gb|AHN96281.1|	37105	37.81	3	1	1	Marurase K, partial (chloroplast) [Zhumeria majdae]
886	gi|602690685|gb|AHN96280.1|	37105	37.81	3	1	1	Marurase K, partial (chloroplast) [Zhumeria majdae]
887	gi|602690677|gb|AHN96276.1|	37105	37.81	3	1	1	Marurase K, partial (chloroplast) [Zhumeria majdae]
888	gi|661525312|gb|AIE15763.1|	216665	33.15	1	1	1	Dicer-like protein 1 [Salvia miltiorrhiza]
889	gi|745790967|gb|AJD25192.1|	58706	32.46	2	1	1	Cytochrome P450 CYP79D40 [Salvia miltiorrhiza]
890	gi|844572703|gb|AKN09562.1|	36568	32.19	2	1	1	Basic helix-loop-helix transcription factor [Salvia miltiorrhiza]
891	gi|29839420|sp|Q9XGV9.1|COMT2_OCIBA	39613	31.81	3	1	1	Caffeic acid 3-O-methyltransferase 2; Short=CAOMT-2; Short=COMT-2; AltName: Full=S-adenosysl-L-methionine:caffeic acid 3-O-methyltransferase 2
892	gi|29839421|sp|Q9XGW0.1|COMT1_OCIBA	39529	24.12	3	1	1	Caffeic acid 3-O-methyltransferase 1; Short=CAOMT-1; Short=COMT-1; AltName: Full=S-adenosysl-L-methionine:caffeic acid 3-O-methyltransferase 1
893	gi|844572794|gb|AKN09591.1|	22976	27.18	4	1	1	Basic helix-loop-helix transcription factor [Salvia miltiorrhiza]
894	gi|910312590|ref|YP_009162251.1|	158800	27.01	1	1	1	DNA-directed RNA polymerase subunit beta''UniRule annotation (EC:2.7.7.6UniRule annotation)

No.	Accession Number	Average Mass	Max-10logP	Max Coverage (%)	Max# Peptides	Max# Unique	Description
895	gi|827345829|gb|AKJ77130.1|	158800	27.01	1	1	1	DNA-directed RNA polymerase subunit beta''UniRule annotation (EC:2.7.7.6UniRule annotation)
896	gi|661525316|gb|AIE15765.1|	184302	25.36	1	1	1	Dicer-like protein 3 [Salvia miltiorrhiza]
897	gi|745791003|gb|AJD25210.1|	57675	23.31	2	1	1	Cytochrome P450 CYP89A115 [Salvia miltiorrhiza]
898	gi|521953403|gb|AGQ04160.1|	40132	21.67	3	1	1	Farnesyl diphosphate synthase [Lavandula angustifolia]
899	gi|630058023|gb|AHY94894.1|	51993	21.30	2	1	1	4-coumarate:CoA ligase, partial [Prunella vulgaris]
900	gi|745790929|gb|AJD25173.1|	57680	21.12	1	1	1	Cytochrome P450 CYP73A120 [Salvia miltiorrhiza]
901	gi|725812545|gb|AIY32618.1|	57979	21.12	1	1	1	Cinnamate-4-hydroxylase [Perilla frutescens]
902	gi|762060297|gb|AJQ20620.1|	48394	21.05	2	1	1	Dihydrolipoamide Acetyltransferase [Salvia miltiorrhiza]
903	gi|548918017|gb|AGX15389.1|	56042	20.86	2	1	1	Flavonoid 3' monooxygenase [Plectranthus barbatus]
904	gi|661525320|gb|AIE15767.1|	174990	20.37	1	1	1	Dicer-like protein 4b [Salvia miltiorrhiza]
